# Constructions of Beyond-Birthday Secure PRFs from Random Permutations, Revisited

**DOI:** 10.3390/e23101296

**Published:** 2021-09-30

**Authors:** Jiehui Nan, Ping Zhang, Honggang Hu

**Affiliations:** 1 Key Laboratory of Electromagnetic Space Information, Chinese Academy of Sciences, School of Information Science and Technology, University of Science and Technology of China, Hefei 230027, China; hghu2005@ustc.edu.cn; 2 School of Computer Science, Nanjing University of Posts and Telecommunications, Nanjing 210023, China; zhgp@njupt.edu.cn

**Keywords:** beyond birthday bound, multi-key security, H-Coefficient technique, nonce based MACs

## Abstract

In CRYPTO 2019, Chen et al. showed how to construct pseudorandom functions (PRFs) from random permutations (RPs), and they gave one beyond-birthday secure construction from sum of Even-Mansour, namely SoEM22 in the single-key setting. In this paper, we improve their work by proving the multi-key security of SoEM22, and further tweaking SoEM22 but still preserving beyond birthday bound (BBB) security. Furthermore, we use only one random permutation to construct parallelizable and succinct beyond-birthday secure PRFs in the multi-key setting, and then tweak this new construction. Moreover, with a slight modification of our constructions of tweakable PRFs, two parallelizable nonce based MACs for variable length messages are obtained.

## 1. Introduction

Random numbers are widely used in engineering practice. In particular, randomization is central to cryptography. One can generate random numbers by using physical random sources such as chaos-based [1] and quantum-based [2] random number generator. However, obtaining random numbers from physical phenomena requires high quality of the entropy source, and is also device-dependent so that the corresponding cost is not cheap. Besides, in some cryptographic applications, the way of generating random numbers above is not friendly due to its uncontrollability. Motivated by cryptographic applications, Blum and Micali [3] and Yao [4] formalized the modern notation of pseudorandom generators from the perspectives in computational complexity. Later, Goldreich et al. [5] proposed the concept of pseudorandom functions (PRFs). Informally, F(K,·) is said to be a PRF where *K* is a uniformly random string with enough entropy, if for any input *x*, F(K,x) can be computed efficiently and can not be distinguished from a truly random value. PRFs are important in cryptography with fruitful applications in encryption, identification, and authentication.

In theory, PRFs can be obtained from one-way functions [5,6], but this general transformation is not practical. Some other algebraic constructions, such as number theory-based [7,8] or lattice-based PRFs [9,10,11], are still inefficient. Therefore, it is significant to construct PRFs from symmetric primitives both in theory and practice. There are a series of works to build the PRFs from pseudorandom permutations (PRPs)/block ciphers [12,13,14]. Recently, Chen et al. [15] proposed a method to construct PRFs from random permutations (RPs). In [15], the construction SoEM22 (which means sum of one-round Even-Mansour based on two independent permutations) was proved beyond-birthday secure in the single-key setting.

About SoEM22, there are three questions we may ask: (i) Is SoEM22 beyond-birthday secure in the multi-key setting? (ii) Can SoEM22 be tweaked while preserving BBB security? (iii) If the underlying random permutations can be computed efficiently in both forward and inverse directions, can we construct beyond-birthday secure PRFs by using only one permutation in both multi-key and tweakable cases?

Fortunately, we can give positive answers to these questions. First, we prove that SoEM22 is beyond-birthday secure in the multi-key setting. Informally, it means that for any distinguisher who distinguishes *m* independent *n*-to-*n*-bit keyed functions from *m* independent ideal random functions, its advantage does not depend on *m*. However, in this case the distinguisher still needs to make at least $O(2^{2n/3})$ queries to achieve a noticeable advantage.

Second, we tweak the construction SoEM22, inspired by the work [16]. A tweakable PRF, $F:K\times T\times {\left\{0,1\right\}}^{n}\to {\left\{0,1\right\}}^{n}$, means that one can associate a tweak space T to the key space K. For any key *k* randomly sampled from K, one can choose different tweaks t∈T to compute y=F(k,t,x) even on the same input *x*.

Following the idea in [17], we solve the third question, and construct beyond-birthday secure PRFs in the multi-key setting from one bidirectionally efficient random permutation. Then this new construction from a single permutation can also be tweaked while preserving BBB security.

### 1.1. Our Contributions

In this paper, we enhance the security of SoEM22 [15] by showing that

(1) $$\begin{array}{c} F_{K_{1},K_{2}}^{P_{1},P_{2}}\left(x\right)=P_{1}\left(x⊕K_{1}\right)⊕P_{2}\left(x⊕K_{2}\right)⊕K_{1}⊕K_{2} \end{array}$$

is beyond-birthday secure in the multi-key setting, where ⊕ denotes the bitwise XOR operator, $P_{1}$ and $P_{2}$ are two independent random permutations, *x* is an *n*-bit input, and $K_{1}$ and $K_{2}$ are two *n*-bit uniformly random strings. Furthermore, we can tweak the construction SoEM22, while preserving BBB security, as

(2) $$\begin{array}{c} {\mathrm{TPRF}}_{H_{K_{h}^{1}},H_{K_{h}^{2}}}^{P_{1},P_{2}}\left(t,x\right)=P_{1}\left(x⊕H_{K_{h}^{1}}\left(t\right)\right)⊕P_{2}\left(x⊕H_{K_{h}^{2}}\left(t\right)\right)⊕H_{K_{h}^{1}}\left(t\right)⊕H_{K_{h}^{2}}\left(t\right), \end{array}$$

where $H_{K_{h}^{1}}$ and $H_{K_{h}^{2}}$ are uniformly and independently sampled from the regular and almost-XOR universal (AXU) keyed hash family, *t* is a tweak, and *x* is an *n*-bit input.

Chen et al. [15] first constructed beyond-birthday secure PRFs from random permutations. Later, Chakraborti et al. [18] suggested and designed minimally structured beyond-birthday secure RPFs (i.e., by using only one random permutation). Following this line of study, we design a parallelizable beyond-birthday secure PRF in the multi-key setting from one bidirectionally efficient random permutation *P* as

(3) $$\begin{array}{c} F_{K_{1},K_{2}}^{P}\left(x\right)=P\left(x⊕K_{1}\right)⊕P^{-1}\left(x⊕K_{2}\right)⊕K_{1}⊕K_{2}, \end{array}$$

where $K_{1}$, $K_{2}$, and *x* are the same as those in Equation (1). We tweak this new construction as

(4) $$\begin{array}{c} {\mathrm{TPRF}}_{H_{K_{h}^{1}},H_{K_{h}^{2}}}^{P}\left(t,x\right)=P\left(x⊕H_{K_{h}^{1}}\left(t\right)\right)⊕P^{-1}\left(x⊕H_{K_{h}^{2}}\left(t\right)\right)⊕H_{K_{h}^{1}}\left(t\right)⊕H_{K_{h}^{2}}\left(t\right), \end{array}$$

where $H_{K_{h}^{1}}$, $H_{K_{h}^{2}}$, *x*, and *t* are the same as those in Equation (2).

Moreover, from our two constructions of tweakble PRFs, we can give two nonce based MACs for variable length messages. In particular, when one replaces the input *x* (resp. the tweak *t*) in Equations (2) and (4) by an *n*-bit nonce *N* (resp. a message *M*), one can obtain two parallelizable beyond-birthday secure nonce based MACs as

(5) $$\begin{array}{c} T=P_{1}\left(N⊕H_{K_{h}^{1}}\left(M\right)\right)⊕P_{2}\left(N⊕H_{K_{h}^{2}}\left(M\right)\right)⊕H_{K_{h}^{1}}\left(M\right)⊕H_{K_{h}^{2}}\left(M\right) \end{array}$$

and

(6) $$\begin{array}{c} T=P\left(N⊕H_{K_{h}^{1}}\left(M\right)\right)⊕P^{-1}\left(N⊕H_{K_{h}^{2}}\left(M\right)\right)⊕H_{K_{h}^{1}}\left(M\right)⊕H_{K_{h}^{2}}\left(M\right). \end{array}$$

### 1.2. Related Works

Based on two random permutations $P_{1}$ and $P_{2}$, Cogliati et al. [16] constructed a beyond-birthday secure tweakable Even-Mansour (TEM) as

(7) $$\begin{array}{c} {\mathrm{TEM}}_{H_{K_{h}^{1}},H_{K_{h}^{2}}}^{P_{1},P_{2}}\left(t,x\right)=P_{2}\left(P_{1}\left(x⊕H_{K_{h}^{1}}\left(t\right)\right)⊕H_{K_{h}^{1}}\left(t\right)⊕H_{K_{h}^{2}}\left(t\right)\right)⊕H_{K_{h}^{2}}\left(t\right), \end{array}$$

where $H_{K_{h}^{1}}$ and $H_{K_{h}^{2}}$ are uniformly and independently sampled from the uniform and AXU keyed hash family, *t* is a tweak, and *x* is an *n*-bit input. Later, Dutta [17] gave a beyond-birthday secure TEM from one permutation as

(8) $$\begin{array}{c} {\mathrm{TEM}}_{H_{K_{h}^{1}},H_{K_{h}^{2}}}^{P}\left(t,x\right)=P\left(P\left(x⊕H_{K_{h}^{1}}\left(t\right)\right)⊕H_{K_{h}^{1}}\left(t\right)⊕H_{K_{h}^{2}}\left(t\right)\right)⊕H_{K_{h}^{2}}\left(t\right), \end{array}$$

where *P* is a random permutation, and $H_{K_{h}^{1}}$, $H_{K_{h}^{1}}$, *t*, and *x* are the same as those in (7). Compared with Equations (7) and (8), our constructions in Equations (2) and (4) are parallelizable.

Chakraborti et al. [18] constructed beyond-birthday secure PRFs from random permutations with minimal structure (i.e., from one random permutation *P*) as

$$P^{-1}\left(P\left(K⊕x\right)⊕3K⊕x\right)⊕2K,$$

where *K* is an *n*-bit key, *x* is an *n*-bit input, and 2 is a primitive element in the finite field $F_{2^{n}}$ so that 2K denotes the multiplication of 2 and *K* over $F_{2^{n}}$. Recently, Dutta et al. [19] proved that the construction

$$P\left(P\left(K_{1}⊕x\right)⊕K_{2}⊕K_{1}⊕x\right)⊕K_{1}$$

is also a beyond-birthday secure PRF, where $K_{1}$ and $K_{2}$ are two *n*-bit uniformly random strings. However, all these two constructions were proved beyond-birthday secure only in the single-key setting. Compared with them, Equation (3) is parallelizable and can be proved beyond-birthday secure in the multi-key setting.

Besides, Chakraborti et al. [18] also gave a nonce based MAC for variable length messages as

$$T=P^{-1}\left(P\left(K⊕N\right)⊕3K⊕N⊕H_{K_{h}}\left(M\right)\right)⊕2K,$$

where *K* is an *n*-bit key, *N* is an *n*-bit nonce, *M* is a variable length message, and $H_{K_{h}}$ is uniformly sampled from the keyed hash family with three properties: regular, AXU, and 3-way regular.

### 1.3. Technical Overview

The basic technique to prove the BBB security of our constructions is the H-Coefficient technique [20,21]. As an example, we intuitively introduce the core idea of the security proof for the construction ${\mathrm{TPRF}}_{H_{K_{h}^{1}},H_{K_{h}^{2}}}^{P}$ in Equation (4). Let Φ be a random function from $T\times {\left\{0,1\right\}}^{n}$ to ${\left\{0,1\right\}}^{n}$, where T is the tweakable space. Denote ${\mathrm{TPRF}}_{H_{K_{h}^{1}},H_{K_{h}^{2}}}^{P}:T\times {\left\{0,1\right\}}^{n}\mapsto {\left\{0,1\right\}}^{n}$ as in Equation (4). Given a deterministic distinguisher *D* who has access query to the primitive oracle *P* and to the construction oracle ${\mathrm{TPRF}}_{H_{K_{h}^{1}},H_{K_{h}^{2}}}^{P}$ or Φ, the goal of *D* is to distinguish which construction oracle it interacts with. Set ${\bar{Q}}_{P}=\left\{\left(u_{1},v_{1}\right),\ldots ,\left(u_{p},v_{p}\right)\right\}$ as all *p* query-response tuples for the primitive oracle, and ${\bar{Q}}_{F}=\left\{\left(t_{1},x_{1},y_{1}\right),\ldots ,\left(t_{q},x_{q},y_{q}\right)\right\}$ as all *q* query-response tuples for the construction oracle. Then, ${\bar{Q}}_{F}$ and ${\bar{Q}}_{P}$ along with $H_{K_{h}^{1}}$ and $H_{K_{h}^{2}}$ are called a transcript, denoted by $\bar{\tau }=\left\{{\bar{Q}}_{F},{\bar{Q}}_{P},\left(H_{K_{h}^{1}},H_{K_{h}^{2}}\right)\right\}$. When *D* interacts with Φ, the transcript $\bar{\tau }$ is said in the ideal world; otherwise, $\bar{\tau }$ is said in the real world.

In general, all possible transcripts are divided into bad transcripts and good transcripts. The key to use the H-Coefficient technique is to define bad transcripts in the ideal world with a low proportion. Furthermore, one also needs to show that the probability of any good transcript in the ideal world is close to its probability in the real world. After observing the transcript, the distinguisher will use this information to test whether it is compatible with ${\mathrm{TPRF}}_{H_{K_{h}^{1}},H_{K_{h}^{2}}}^{P}$. Based on this fact, one can briefly interpret how to define bad transcripts by the following example. Assume that there exist $\left(t,x,y\right)\in {\bar{Q}}_{F}$ and $\left(u_{1},v_{1}\right),\left(u_{2},v_{2}\right)\in {\bar{Q}}_{P}$ such that $H_{K_{h}^{1}}\left(t\right)⊕x=u_{1}$ and $H_{K_{h}^{2}}\left(t\right)⊕x=v_{2}$ (this event is denoted by ${\mathrm{Bad}}_{1}$). Then in the real world, one must have $y=v_{1}⊕u_{2}⊕H_{K_{h}^{1}}\left(t\right)⊕H_{K_{h}^{2}}\left(t\right)$. However, in the ideal world, the probability that this equation holds is at most $1/2^{n}$. In this case, the distinguisher has a significant advantage. If $H_{K_{h}^{1}}$ and $H_{K_{h}^{2}}$ are independently chosen from the uniform keyed hash family, then one has

$$\mathrm{Pr}\left[\left(H_{K_{h}^{1}}\left(t\right)=x⊕u_{1}\right)\wedge \left(H_{K_{h}^{2}}\left(t\right)=x⊕v_{2}\right)\right]\leq \frac{1}{2^{2n}}.$$

**Figure 1:**
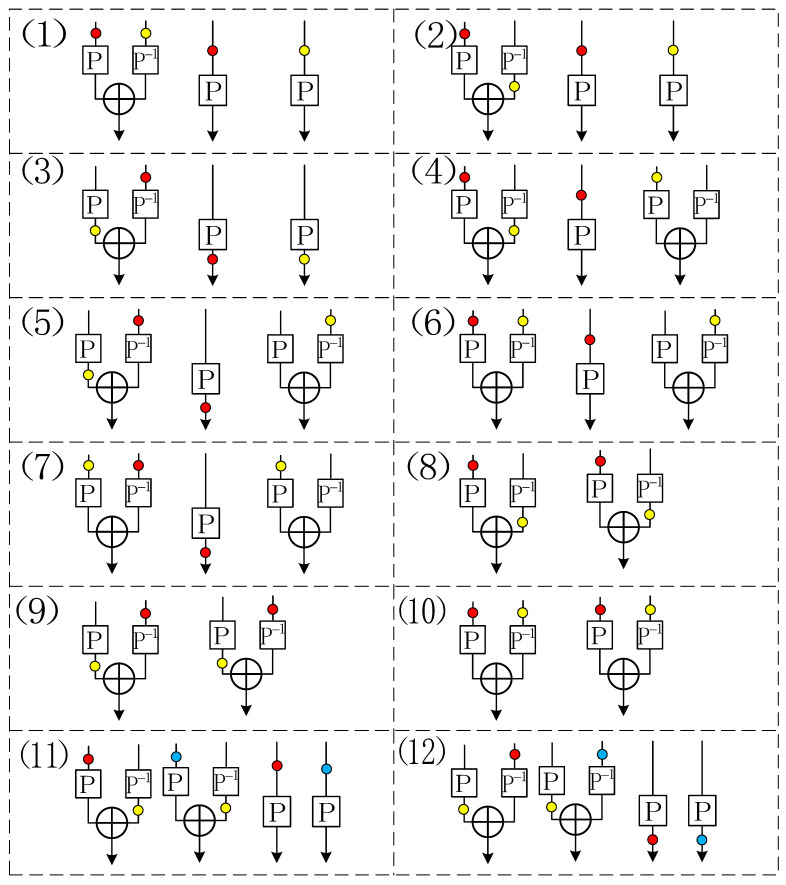
Graphical representation of the motivation to define bad cases for the transcript in the ideal world, which corresponds to the bad conditions from (C-1) to (C-12) in Section 4. In this graph, the same color in different lines means that there exists a collision between these places.

By union bound, the probability of ${\mathrm{Bad}}_{1}$ in the ideal world can be upper bounded by $qp^{2}/2^{2n}$. This advantage is secure roughly up to $p=q=O(2^{2n/3})$ adversarial queries. We illustrate some other bad cases for transcript $\bar{\tau }$ in Figure 1, where (1) in Figure 1 is for the above example.

For any good transcript, to prove that its probability in the real world is almost close to the one in the ideal world, it needs to show that the number of choices for unfixed maps of *P* is large enough. Let $U=\{u_{1}\in {\left\{0,1\right\}}^{n}:\left(u_{1},v_{1}\right)\in {\bar{Q}}_{P}\}$, $V=\{v_{1}\in {\left\{0,1\right\}}^{n}:\left(u_{1},v_{1}\right)\in {\bar{Q}}_{P}\}$, $U_{F}=\left\{H_{K_{h}^{1}}\left(t\right)⊕x:\left(t,x,y\right)\in {\bar{Q}}_{F}\right\}$, and $V_{F}=\left\{H_{K_{h}^{2}}\left(t\right)⊕x:\left(t,x,y\right)\in {\bar{Q}}_{F}\right\}$. Then the good transcript ensures that $U\cap U_{F}=\emptyset$ (resp. $V\cap V_{F}=\emptyset$) and all items in $U_{F}$ (resp. $V_{F}$) are distinct. The next goal is to choose distinct values for $\left\{P\left(H_{K_{h}^{1}}\left(t\right)⊕x\right):\left(t,x,y\right)\in {\bar{Q}}_{F}\right\}$ (resp. $\left\{P^{-1}\left(H_{K_{h}^{2}}\left(t\right)⊕x\right):\left(t,x,y\right)\in {\bar{Q}}_{F}\right\}$) such that $\left\{P\left(H_{K_{h}^{1}}\left(t\right)⊕x\right):\left(t,x,y\right)\in {\bar{Q}}_{F}\right\}\cap \left(V\cup V_{F}\right)=\emptyset$, $\left\{P\left(H_{K_{h}^{1}}\left(t\right)⊕x\right)⊕H_{K_{h}^{1}}\left(t\right)⊕H_{K_{h}^{2}}\left(t\right)⊕y:\left(t,x,y\right)\in {\bar{Q}}_{F}\right\}\cap \left(U\cup U_{F}\right)=\emptyset$, and all items in $\left\{P\left(H_{K_{h}^{1}}\left(t\right)⊕x\right)⊕H_{K_{h}^{1}}\left(t\right)⊕H_{K_{h}^{2}}\left(t\right)⊕y:\left(t,x,y\right)\in {\bar{Q}}_{F}\right\}$ are distinct (resp. $\left\{P^{-1}\left(H_{K_{h}^{2}}\left(t\right)⊕x\right):\left(t,x,y\right)\in {\bar{Q}}_{F}\right\}\cap \left(U\cup U_{F}\right)=\emptyset$, $\left\{P^{-1}\left(H_{K_{h}^{2}}\left(t\right)⊕x\right)⊕H_{K_{h}^{1}}\left(t\right)⊕H_{K_{h}^{2}}\left(t\right)⊕y:\left(t,x,y\right)\in {\bar{Q}}_{F}\right\}\cap \left(V\cup V_{F}\right)=\emptyset$, and all items in $\left\{P^{-1}\left(H_{K_{h}^{2}}\left(t\right)⊕x\right)⊕H_{K_{h}^{1}}\left(t\right)⊕H_{K_{h}^{2}}\left(t\right)⊕y:\left(t,x,y\right)\in {\bar{Q}}_{F}\right\}$ are distinct). However, this strategy is not enough to achieve the BBB security. To deal with this problem, we adopt the main idea in [17,22] to count more possible choices for unfixed maps of *P*, and this idea allows that $\left\{P\left(H_{K_{h}^{1}}\left(t\right)⊕x\right):\left(t,x,y\right)\in {\bar{Q}}_{F}\right\}\cap V_{F}\neq \emptyset$. Informally, it means that there exist some pairs $\left(\left(t,x,y\right),\left(t^{\prime },x^{\prime },y^{\prime }\right)\right)\in {\bar{Q}}_{F}\times {\bar{Q}}_{F}$ such that $P\left(x⊕H_{K_{h}^{1}}\left(t\right)\right)⊕H_{K_{h}^{1}}\left(t\right)⊕H_{K_{h}^{2}}\left(t\right)⊕y=x^{\prime }⊕H_{K_{h}^{1}}\left(t^{\prime }\right)$ or $P^{-1}\left(x⊕H_{K_{h}^{2}}\left(t\right)\right)⊕H_{K_{h}^{1}}\left(t\right)⊕H_{K_{h}^{2}}\left(t\right)⊕y=x^{\prime }⊕H_{K_{h}^{2}}\left(t^{\prime }\right)$. Take the first case for example, one has

(9) $$\left\{ \begin{array}{c} x⊕H_{K_{h}^{1}}\left(t\right)\overset{P}{⟼}x^{\prime }⊕H_{K_{h}^{1}}\left(t^{\prime }\right)⊕H_{K_{h}^{1}}\left(t\right)⊕H_{K_{h}^{2}}\left(t\right)⊕y,\\ x^{\prime }⊕H_{K_{h}^{1}}\left(t^{\prime }\right)\overset{P}{⟼}x⊕H_{K_{h}^{2}}\left(t\right),\\ x⊕H_{K_{h}^{2}}\left(t\right)⊕H_{K_{h}^{1}}\left(t^{\prime }\right)⊕H_{K_{h}^{2}}\left(t^{\prime }\right)⊕y^{\prime }\overset{P}{⟼}x^{\prime }⊕H_{K_{h}^{2}}\left(t^{\prime }\right). \end{array}\right.$$

To ensure that the maps in (9) are valid, $x^{\prime }⊕H_{K_{h}^{1}}\left(t^{\prime }\right)⊕H_{K_{h}^{1}}\left(t\right)⊕H_{K_{h}^{2}}\left(t\right)⊕y$ can not be equal to previous fixed inputs of *P*, and $x⊕H_{K_{h}^{2}}\left(t\right)⊕H_{K_{h}^{1}}\left(t^{\prime }\right)⊕H_{K_{h}^{2}}\left(t^{\prime }\right)⊕y^{\prime }$ can not be equal to previous fixed outputs of *P*. Since Φ is a random function from $T\times {\left\{0,1\right\}}^{n}$ to ${\left\{0,1\right\}}^{n}$, then y=Φ(t,x) is uniformly and independently distributed for each distinct query (t,x) in the ideal world. Due to this property, one can define the good transcripts to ensure that the number of rational maps in (9) is large enough. At the same time, it guarantees that the proportion of the corresponding bad transcripts in the ideal world can also achieve a beyond birthday bound. For more details, please refer to Section 4.

### 1.4. Organization

The rest of this paper is organized as follows. In Section 2, we introduce some necessary notations and basic tools. In Section 3, we prove the multi-key security of SoEM22, further tweak the construction SoEM22, and finally construct parallelizable nonce based MACs from two permutations. The constructions of beyond-birthday secure PRFs from one permutation in both multi-key and tweakable settings are given in Section 4, and we also design parallelizable nonce based MACs from one permutation in this section. Finally, Section 5 concludes this paper.

## 2. Preliminaries

### 2.1. Notations

For any n∈Z, we simplify the set {1,…,n} as [n], and denote the set of all *n*-bit strings by ${\left\{0,1\right\}}^{n}$. For any finite set S, $s\overset{\$}{\leftarrow }S$ means that *s* is sampled uniformly from S. Besides, |S| denotes the size of *S*. For any sets X and Y, Func(X,Y) includes all functions from X to Y, and we simply write Func(n) for $\mathrm{Func}({\left\{0,1\right\}}^{n},{\left\{0,1\right\}}^{n})$. Furthermore, Perm(n) denotes the set of all permutations on ${\left\{0,1\right\}}^{n}$. For any two integers *q* and *N* such that 1≤q≤N, define ${\left(N\right)}_{q}=N\left(N-1\right)\ldots \left(N-q+1\right)$. In particular, ${\left(N\right)}_{0}=1$.

$Q=\{\left(x_{1},y_{1}\right),\ldots ,\left(x_{p},y_{p}\right)\}$ is said a well-defined *n*-bit permutation-compatible set if $x_{1},\ldots ,x_{p}\in {\left\{0,1\right\}}^{n}$ (resp. $y_{1},\ldots ,y_{p}\in {\left\{0,1\right\}}^{n}$) are all distinct. Given a well-defined permutation-compatible set Q, we say that the permutation P∈Perm(n) extends Q, denoted by P⊢Q, if $P\left(x_{i}\right)=y_{i}$ for all i∈[p]. For another well-defined *n*-bit permutation-compatible set $Q^{\prime }=\left\{\left(x_{1}^{\prime },y_{1}^{\prime }\right),\ldots ,\left(x_{p^{\prime }}^{\prime },y_{p^{\prime }}^{\prime }\right)\right\}$, $Q^{\prime }$ and Q are called disjoint if $x_{i}\neq x_{j}^{\prime }$ and $y_{i}\neq y_{j}^{\prime }$ for any i∈[p] and $j\in [p^{\prime }]$. Given the disjoint *n*-bit permutation-compatible set Q and $Q^{\prime }$, for any random permutation $P\overset{\$}{\leftarrow }\mathrm{Perm}\left(n\right)$ satisfying P⊢Q, the probability of $P⊢Q^{\prime }$ is $1/{\left(2^{n}-p\right)}_{p^{\prime }}$, which is denoted by

$$\mathrm{Pr}\left[P\overset{\$}{\leftarrow }\mathrm{Perm}\left(n\right):P⊢Q^{\prime }|P⊢Q\right]=\frac{1}{{\left(2^{n}-p\right)}_{p^{\prime }}}.$$

For any function F:D→V, given the set $S=\{\left(x_{1},y_{1}\right),\ldots ,\left(x_{q},y_{q}\right):\left(x_{i},y_{i}\right)\in D\times V\}$, F⊢S means that $F\left(x_{i}\right)=y_{i}$ for any $(x_{i},y_{i})\in S$.

Given two sets *U* and $U^{\prime }$, we say that *U* is disjoint with $U^{\prime }$ if $U\cap U^{\prime }=\emptyset$. Let $U=\{U_{1},\ldots ,U_{m}\}$ be a collection of finite sets. Then U is called a disjoint collection if for any i≠j∈[m], $U_{i}$ is disjoint with $U_{j}$. In this case, the size of U is defined as $\left|U|=\right|U_{1}\left|+\ldots +\right|U_{m}|$. Two disjoint collections $U=\{U_{1},\ldots ,U_{m}\}$ and $U^{\prime }=\left\{U_{1}^{\prime },\ldots ,U_{n}^{\prime }\right\}$ are called inner disjoint if $U_{i}\cap U_{i^{\prime }}^{\prime }=\emptyset$ for any $i\in \left[m\right],i^{\prime }\in \left[n\right]$. Let $S_{\mathrm{mul}}$ be a multi-set, and let $\delta_{S_{\mathrm{mul}}}\left(x\right)$ denote the multiplicity of *x* in $S_{\mathrm{mul}}$. When $S_{\mathrm{mul}}$ is called a set, it means that all the repeated items in it are viewed as a unique item. Throughout this paper, when we discuss the size of $S_{\mathrm{mul}}$, which is denoted by $|S_{\mathrm{mul}}|$, the items in $S_{\mathrm{mul}}$ are counted without considering the multiplicity.

**Definition** **1** (Universal Hash Functions). *Let n be a positive integer. Assume that $K_{H}$ and X are two finite sets. Let $H={\left(H_{K_{h}}\right)}_{K_{h}\in K_{H}}$ be a keyed hash family from X to ${\left\{0,1\right\}}^{n}$, where $K_{H}$ is the hash key space. H is called $\epsilon_{1}$-regular if for any t∈X and any $y\in {\left\{0,1\right\}}^{n}$, it holds that*

$$\mathrm{Pr}\left[K_{h}\overset{\$}{\leftarrow }K_{H}:H_{K_{h}}\left(t\right)=y\right]\leq \epsilon_{1}.$$

*H is called $\epsilon_{2}$-almost XOR-universal ($\epsilon_{2}$-AXU) if for any distinct $t,t^{\prime }\in X$ and any $y\in {\left\{0,1\right\}}^{n}$, it holds that*

$$\mathrm{Pr}\left[K_{h}\overset{\$}{\leftarrow }K_{H}:H_{K_{h}}\left(t\right)⊕H_{K_{h}}\left(t^{\prime }\right)=y\right]\leq \epsilon_{2}.$$

*H is said XOR-universal (resp. uniform) if it is $2^{-n}$-AXU (resp. $2^{-n}$-regular).*

Next, we briefly describe an example of $\frac{l}{2^{n}}$-regular and $\frac{l}{2^{n}}$-AXU keyed hash family [18,23] for some constant l∈N. Let *M* be any binary string with |M|<l·n, and set $K_{H}={\left\{0,1\right\}}^{n}$. Then we pad *M* as $M||10^{s}=M_{1}\left||\ldots |\right|M_{l}$, where s=l·n−|M|−1, $0^{s}$ denotes the all zero *s* bits, and $M_{i}\in {\left\{0,1\right\}}^{n}$ for each i∈[l]. For any $K_{h}\in K_{H}$, the keyed hash is defined as:

(10) $$\begin{array}{c} {\mathrm{Poly}}_{H_{K_{h}}}\left(M\right)=M_{l}\cdot K_{h}⊕M_{l-1}\cdot K_{h}^{2}⊕\ldots ⊕M_{1}\cdot K_{h}^{l}, \end{array}$$

where $K_{h}$ and $M_{i}$ (i∈[l]) are viewed as the elements in $F_{2^{n}}$, and · denotes the multiplication in $F_{2^{n}}$.

**Remark** **1.** *The keyed hash family H is said to be ϵ-3-way regular, if for any $y\in {\left\{0,1\right\}}^{n}$ and any three distinct inputs t, $t^{\prime }$, and $t^{\prime \prime }\in X$, it holds that*

$$\mathrm{Pr}[K_{h}\overset{\$}{\leftarrow }K_{H}:H_{K_{h}}\left(t\right)⊕H_{K_{h}}\left(t^{\prime }\right)⊕H_{K_{h}}\left(t^{\prime \prime }\right)=y]\leq \epsilon .$$

### 2.2. The H-Coefficient Technique

One important tool used in our proofs is the H-Coefficient technique [21], which can be used to upper bound the statistical distance between the query-answers from two interactive systems. For convenience, we focus on the modernization version of Chen and Steinberger [20].

Let $P_{1},\ldots ,P_{r}\overset{\$}{\leftarrow }\mathrm{Perm}\left(n\right)$ be *r* independent random permutations, and K be the key space. In this paper, we only consider the case r∈{1,2} and $K={\left\{0,1\right\}}^{2n}$. The randomly sampled 2n-bit key can be parsed as $\left(K_{1},K_{2}\right)\overset{\$}{\leftarrow }{\left\{0,1\right\}}^{2n}$, where $K_{1}$ and $K_{2}$ are two independent *n*-bit uniformly random strings. Then based on *r* public permutations $P_{1},\ldots ,P_{r}$, $F_{K_{1},K_{2}}^{P_{1},\ldots ,P_{r}}:{\left\{0,1\right\}}^{n}\to {\left\{0,1\right\}}^{n}$ denotes the keyed function indexed by $\left(K_{1},K_{2}\right)\in {\left\{0,1\right\}}^{2n}$. Besides, let $\varphi \overset{\$}{\leftarrow }\mathrm{Func}\left(n\right)$ be an ideal random function. Then for any deterministic distinguisher D who has query access to the oracle $O_{\mathrm{re}}=\left(F_{K_{1},K_{2}}^{P_{1},\ldots ,P_{r}};P_{1}^{\pm },\ldots ,P_{r}^{\pm }\right)$ in the real world, or the oracle $O_{\mathrm{id}}=\left(\varphi ;P_{1}^{\pm },\ldots ,P_{r}^{\pm }\right)$ in the ideal world, the advantage of D to distinguish which oracle it has access to is defined by

(11) $$\begin{array}{c} {\mathrm{Adv}}_{F}\left(D\right)=\left|\mathrm{Pr}\left[D^{O_{\mathrm{re}}}=1\right]-\mathrm{Pr}\left[D^{O_{\mathrm{id}}}=1\right]\right|. \end{array}$$

**Figure 2:**
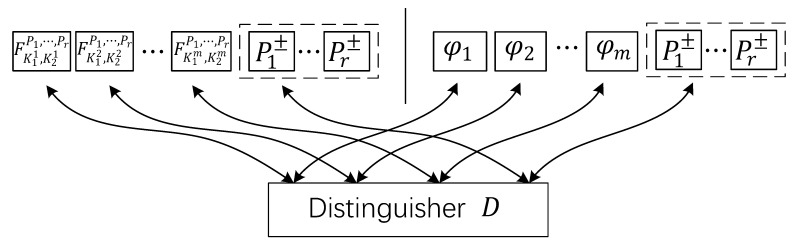
The illustration of the RP-based keyed function $F_{K_{1},K_{2}}^{P_{1},\ldots ,P_{r}}$ in the multi-key setting, where the distinguisher *D* interacts with the real oracle at left, and with the ideal oracle at right.

As shown in Figure 2, in the multi-key setting, the goal of distinguisher D is to distinguish *m* keyed functions $\left(F_{K_{1}^{1},K_{2}^{1}}^{P_{1},\ldots ,P_{r}},\ldots ,F_{K_{1}^{m},K_{2}^{m}}^{P_{1},\ldots ,P_{r}}\right)$ from *m* independent ideal random functions $\varphi_{1},\ldots ,\varphi_{m}\overset{\$}{\leftarrow }\mathrm{Func}\left(n\right)$, where $\left(K_{1}^{1},K_{2}^{1}\right),\ldots ,\left(K_{1}^{m},K_{2}^{m}\right)\overset{\$}{\leftarrow }{\left\{0,1\right\}}^{2n}$ are *m* independent keys. In this case, let $O_{\mathrm{id}}=(\varphi_{1},\ldots ,\varphi_{m},\;P_{1}^{\pm },\ldots ,P_{r}^{\pm })$ be the oracle in the ideal world, and $O_{\mathrm{re}}=(F_{K_{1}^{1},K_{2}^{1}}^{P_{1},\ldots ,P_{r}},\ldots ,F_{K_{1}^{m},K_{2}^{m}}^{P_{1},\ldots ,P_{r}},\;P_{1}^{\pm },\ldots ,P_{r}^{\pm })$ be the oracle in the real world. The advantage of the distinguisher D to distinguish these two oracles can be defined as the same in (11), but here we use ${\mathrm{Adv}}_{F_{K_{1},K_{2}}^{P_{1},\ldots ,P_{r}}}^{mk}\left(D\right)$ to identify the multi-key case.

Let H be an $\epsilon_{1}$-regular and $\epsilon_{2}$-AXU keyed hash family from T to ${\left\{0,1\right\}}^{n}$. Then we use two independent keyed hash functions $\left(H_{K_{h}^{1}},H_{K_{h}^{2}}\right)\overset{\$}{\leftarrow }H^{2}$ to tweak the keyed function $F_{K_{1},K_{2}}^{P_{1},\ldots ,P_{r}}$ as ${\mathrm{TPRF}}_{H_{K_{h}^{1}},H_{K_{h}^{2}}}^{P_{1},\ldots ,P_{r}}:T\times {\left\{0,1\right\}}^{n}\to {\left\{0,1\right\}}^{n}$ such that ${\mathrm{TPRF}}_{H_{K_{h}^{1}},H_{K_{h}^{2}}}^{P_{1},\ldots ,P_{r}}\left(t,x\right)=F_{\left(H_{K_{h}^{1}}\left(t\right),H_{K_{h}^{2}}\left(t\right)\right)}^{P_{1},\ldots ,P_{r}}\left(x\right)$. In addition, the ideal tweakable random function can be denoted as $\Phi :T\times {\left\{0,1\right\}}^{n}\to {\left\{0,1\right\}}^{n}$, i.e., $\Phi \overset{\$}{\leftarrow }\mathrm{Func}\left(T\times {\left\{0,1\right\}}^{n},{\left\{0,1\right\}}^{n}\right)$. In this case, let $O_{\mathrm{re}}=({\mathrm{TPRF}}_{H_{K_{1}},H_{K_{2}}}^{P_{1},\ldots ,P_{r}},\;P_{1}^{\pm },\ldots ,P_{r}^{\pm })$ be the oracle in the real world, and $O_{\mathrm{id}}=(\Phi ,\;P_{1}^{\pm },\ldots ,P_{r}^{\pm })$ be the oracle in the ideal world. For any distinguisher D, its advantage can be defined as the same in (11), but here we use ${\mathrm{Adv}}_{{\mathrm{TPRF}}_{H_{K_{h}^{1}},H_{K_{h}^{2}}}^{P_{1},\ldots ,P_{r}}}^{tweak}\left(D\right)$ to identify the tweakable case.

The security proofs in both multi-key and tweakable settings are similar. Therefore, we prove these two cases in a unified approach. For two independently and randomly sampled functions $f_{1}$ and $f_{2}$ from $\mathrm{Func}(T,{\left\{0,1\right\}}^{n})$, $\left(f_{1},f_{2}\right)$ is said a good $\left(\epsilon_{1},\epsilon_{2}\right)$-key-derivation pair if it satisfies two properties in the following:

(i) $\epsilon_{1}$-Regular. For any t∈T and any $y\in {\left\{0,1\right\}}^{n}$, it holds that
  $$\mathrm{Pr}\left[f_{i}\left(t\right)=y\right]\leq \epsilon_{1},\;\mathrm{for}\;i\in \left\{1,2\right\}.$$
(ii) $\epsilon_{2}$-AXU. For any distinct $t,t^{\prime }\in T$ and any $y\in {\left\{0,1\right\}}^{n}$, it holds that
  $$\mathrm{Pr}\left[f_{i}\left(t\right)⊕f_{i}\left(t^{\prime }\right)=y\right]\leq \epsilon_{2},\;\mathrm{for}\;i\in \left\{1,2\right\}.$$

The above two properties are enough for the security proofs in both tweakable and multi-key settings. In the tweakable setting, $\left(H_{K_{h}^{1}},H_{K_{h}^{2}}\right)$ is a good $\left(\epsilon_{1},\epsilon_{2}\right)$-key-derivation pair, where $(H_{K_{h}^{1}},\;H_{K_{h}^{2}})\overset{\$}{\leftarrow }H^{2}$. In the multi-key setting, set T=[m], and uniformly and randomly sample two independent random functions $f_{1},f_{2}\overset{\$}{\leftarrow }\mathrm{Func}\left(T,{\left\{0,1\right\}}^{n}\right)$. Then $\left(f_{1},f_{2}\right)$ is a good $\left(2^{-n},2^{-n}\right)$-key-derivation pair. To show the security of the constructions in both tweakable and multi-key settings, we only need to prove the BBB security of the following “unified” function

$$\begin{array}{c} F_{f_{1},f_{2}}^{P_{1},\ldots ,P_{r}}:T\times {\left\{0,1\right\}}^{n}\to {\left\{0,1\right\}}^{n}, \end{array}$$

where $\left(f_{1},f_{2}\right)$ is a good $\left(\epsilon_{1},\epsilon_{2}\right)$-key-derivation pair and $P_{1},\ldots ,P_{r}$ (r∈{1,2}) are *r* independent random permutations. In this case, let $O_{\mathrm{re}}=(F_{f_{1},f_{2}}^{P_{1},\ldots ,P_{r}},\;P_{1}^{\pm },\ldots ,P_{r}^{\pm })$ be the oracle in the real world, and $O_{\mathrm{id}}=\left(\Phi ,P_{1}^{\pm },\ldots ,P_{r}^{\pm }\right)$ be the oracle in the ideal world, where $\Phi \overset{\$}{\leftarrow }\mathrm{Func}\left(T\times {\left\{0,1\right\}}^{n},{\left\{0,1\right\}}^{n}\right)$. When the distinguisher D interactes with $O_{\mathrm{re}}$ or $O_{\mathrm{id}}$, any query-responses along with the good $\left(\epsilon_{1},\epsilon_{2}\right)$-key-derivation pair $\left(f_{1},f_{2}\right)\in \mathrm{Func}{\left(T,{\left\{0,1\right\}}^{n}\right)}^{2}$ are called a transcript, denoted by $\tau =(Q_{F},Q_{P_{1}},\ldots ,Q_{P_{r}},\left(f_{1},f_{2}\right))$. In addition, $Q_{F}$ (resp. $Q_{P_{i}}$, for 1≤i≤r) records query-responses when the distinguisher *D* interacts with the construction oracle (resp. the primitive oracle $P_{i}$ for 1≤i≤r). Furthermore, $T_{\mathrm{re}}$ (resp. $T_{\mathrm{id}}$) denotes the probability distribution of the interacting transcripts between D and $O_{\mathrm{re}}$ (resp. $O_{\mathrm{id}}$). A transcript τ is said attainable if $\mathrm{Pr}[T_{\mathrm{id}}=\tau ]>0$. Finally, the advantage of the distinguisher D, to distinguish which oracle it has access to, can be defined as the same in (11), but here we use ${\mathrm{Adv}}_{F_{f_{1},f_{2}}^{P_{1},\ldots ,P_{r}}}^{unify}\left(D\right)$ to identify this unified description.

Let $\Gamma =\Gamma_{\mathrm{good}}\cup \Gamma_{\mathrm{bad}}$ be a partition for the set Γ consisting of all attainable transcripts, where $\Gamma_{\mathrm{good}}$ (resp. $\Gamma_{\mathrm{bad}}$) contains all “good” (resp. “bad”) transcripts. Then the main result of the H-Coefficient technique can be described as the following lemma.

**Lemma** **1** (H-Coefficient Technique [20,21]). *Let D be a deterministic distinguisher, and $T_{\mathrm{re}}$ (resp. $T_{\mathrm{id}}$) be the probability distribution of transcripts in the real world (resp. in the ideal world). Let $\Gamma_{\mathrm{good}}$ and $\Gamma_{\mathrm{bad}}$ be defined above. Assume that there exists $0\leq \epsilon_{\mathrm{ratio}}\leq 1$ such that for any $\tau \in \Gamma_{\mathrm{good}}$, it holds that*

$$\begin{array}{c} \frac{\mathrm{Pr}[T_{\mathrm{re}}=\tau ]}{\mathrm{Pr}[T_{\mathrm{id}}=\tau ]}\geq 1-\epsilon_{\mathrm{ratio}}. \end{array}$$

*Then, ${\mathrm{Adv}}_{F_{f_{1},f_{2}}^{P_{1},\ldots ,P_{r}}}^{unify}\left(D\right)\leq \epsilon_{\mathrm{ratio}}+\mathrm{Pr}\left[T_{\mathrm{id}}\in \Gamma_{\mathrm{bad}}\right]$.*

### 2.3. Useful Tools

Assume that there are g “rational” items in an *N*-size set *S*. When one samples s items from *S* without replacement, H denotes the random variable which counts the number of “rational” items among these s items. Then we say that H follows the hypergeometric distribution with parameters *N*, s, and g, denoted by $H∼{\mathrm{Hyp}}_{N,s,g}$. For 0≤α≤g, one has

Pr[H=α]=gα·N−gs−αNs.

In addition, the expectation value of H is sg/N, i.e., E(H)=sg/N.

The following lemma is useful in our proofs.

**Lemma** **2.** *Let A, B, C, and N be positive integers satisfying A+B≤N/2 and A+C≤N/2. Then we have*

$$\prod_{j=0}^{A-1}\frac{N(N-B-C-2j)}{\left(N-B-j)(N-C-j\right)}\geq 1-\frac{4A(A+B)(A+C)}{N^{2}}.$$

**Proof.**  $$\begin{array}{cc} \prod_{j=0}^{A-1}\frac{N(N-B-C-2j)}{\left(N-B-j)(N-C-j\right)} & =\prod_{j=0}^{A-1}\frac{\left(N-B-j)(N-C-j)-(B+j)(C+j\right)}{\left(N-B-j)(N-C-j\right)}\\ \; & =\prod_{j=0}^{A-1}\left(1-\frac{\left(B+j)(C+j\right)}{\left(N-B-j)(N-C-j\right)}\right)\\ \; & \geq \prod_{j=0}^{A-1}\left(1-\frac{\left(B+A)(C+A\right)}{\left(N-B-A)(N-C-A\right)}\right)\\ \; & \overset{\left(*\right)}{\geq }\prod_{j=0}^{A-1}\left(1-\frac{4(B+A)(C+A)}{N^{2}}\right)\\ \; & \geq \left(1-\frac{4A(B+A)(C+A)}{N^{2}}\right), \end{array}$$

where (*) holds since A+B≤N/2 and A+C≤N/2.  □

## 3. Multi-Key and Tweakable Secure PRFs from Two Random Permutations

In this section, we prove that the construction SoEM22 from two random permutations $P_{1},P_{2}\overset{\$}{\leftarrow }\mathrm{Perm}\left(n\right)$ in [15], namely

(12) $$\begin{array}{c} F_{K_{1},K_{2}}^{P_{1},P_{2}}\left(x\right)=P_{1}\left(x⊕K_{1}\right)⊕P_{2}\left(x⊕K_{2}\right)⊕K_{1}⊕K_{2}, \end{array}$$

is beyond-birthday secure in the multi-key setting, where $\left(K_{1},K_{2}\right)\overset{\$}{\leftarrow }{\left\{0,1\right\}}^{2n}$ and $x\in {\left\{0,1\right\}}^{n}$.

Let H be an $\epsilon_{1}$-regular and $\epsilon_{2}$-AXU keyed hash family from T to ${\left\{0,1\right\}}^{n}$. Then we can tweak SoEM22 as

(13) $$\begin{array}{c} {\mathrm{TPRF}}_{H_{K_{h}^{1}},H_{K_{h}^{2}}}^{P_{1},P_{2}}\left(t,x\right)=P_{1}\left(x⊕H_{K_{h}^{1}}\left(t\right)\right)⊕P_{2}\left(x⊕H_{K_{h}^{2}}\left(t\right)\right)⊕H_{K_{h}^{1}}\left(t\right)⊕H_{K_{h}^{2}}\left(t\right), \end{array}$$

where t∈T, $x\in {\left\{0,1\right\}}^{n}$, and $\left(H_{K_{h}^{1}},H_{K_{h}^{2}}\right)\overset{\$}{\leftarrow }H^{2}$.

To show the security of SoEM22 in both multi-key and tweakable settings above, we only need to prove the BBB security of the following “unified” function

(14) $$\begin{array}{c} F_{f_{1},f_{2}}^{P_{1},P_{2}}\left(t,x\right)=P_{1}\left(x⊕f_{1}\left(t\right)\right)⊕P_{2}\left(x⊕f_{2}\left(t\right)\right)⊕f_{1}\left(t\right)⊕f_{2}\left(t\right), \end{array}$$

where $P_{1},P_{2}\overset{\$}{\leftarrow }\mathrm{Perm}\left(n\right)$, $\left(f_{1},f_{2}\right)\in \mathrm{Func}{\left(T,{\left\{0,1\right\}}^{n}\right)}^{2}$ is a good $\left(\epsilon_{1},\epsilon_{2}\right)$-key-derivation pair, t∈T, and $x\in {\left\{0,1\right\}}^{n}$.

**Theorem** **1.** *Let n∈N, and $\left(f_{1},f_{2}\right)\in \mathrm{Func}{\left(T,{\left\{0,1\right\}}^{n}\right)}^{2}$ be a good $\left(\epsilon_{1},\epsilon_{2}\right)$-key-derivation pair. Consider the function $F_{f_{1},f_{2}}^{P_{1},P_{2}}:T\times {\left\{0,1\right\}}^{n}\to {\left\{0,1\right\}}^{n}$ defined in (14) based on two random permutations $P_{1},P_{2}\overset{\$}{\leftarrow }\mathrm{Perm}\left(n\right)$. For any deterministic distinguisher D making at most $p_{1}$ queries to $P_{1}$, $p_{2}$ queries to $P_{2}$, and q queries to construction oracle $F_{f_{1},f_{2}}^{P_{1},P_{2}}$ or Φ such that $p_{1}+p_{2}+3q\leq 2^{n-1}$, we have*

(15) $$\begin{array}{cc} {\mathrm{Adv}}_{F_{f_{1},f_{2}}^{P_{1},P_{2}}}^{unify}\left(D\right)\leq & 3qp_{1}p_{2}\epsilon_{1}^{2}+\frac{\epsilon_{1}\left(\epsilon_{2}q^{2}+2\sqrt{q}\right)\left(p_{1}+p_{2}\right)}{2}+2\epsilon_{2}^{2}q^{3}+\epsilon_{2}q^{3/2}\\ \; & +\frac{4q{\left(p_{1}+p_{2}+2q\right)}^{2}}{2^{2n}}+\frac{2\sqrt{q}\left(p_{1}+p_{2}\right)}{2^{n}}+\frac{11q}{2^{n}}. \end{array}$$

In the multi-key setting, one sets T=[m] corresponding to *m* independent random keys, and randomly samples two independent random functions $f_{1},f_{2}\overset{\$}{\leftarrow }\mathrm{Func}\left(\left[m\right],{\left\{0,1\right\}}^{n}\right)$. Then we can easily conclude that $\left(f_{1},f_{2}\right)$ is a good $\left(2^{-n},2^{-n}\right)$-key-derivation pair. By this fact, one can obtain the following corollary.

**Corollary** **1.** *Let n,m∈N. Consider the keyed function $F_{K_{1},K_{2}}^{P_{1},P_{2}}:{\left\{0,1\right\}}^{n}\to {\left\{0,1\right\}}^{n}$ defined in (12) based on two random permutations $P_{1},P_{2}\overset{\$}{\leftarrow }\mathrm{Perm}\left(n\right)$. For any deterministic distinguisher D making at most $p_{1}$ queries to $P_{1}$, $p_{2}$ queries to $P_{2}$, and totally q queries to $F_{K_{1}^{1},K_{2}^{1}}^{P_{1},P_{2}},\ldots ,F_{K_{1}^{m},K_{2}^{m}}^{P_{1},P_{2}}$ (resp. m independent ideal random functions $\varphi_{1},\ldots ,\varphi_{m}$) such that $p_{1}+p_{2}+3q\leq 2^{n-1}$, we have*

(16) $$\begin{array}{cc} {\mathrm{Adv}}_{F_{K_{1},K_{2}}^{P_{1},P_{2}}}^{mk}\left(D\right)\leq & \frac{3p_{1}p_{2}q}{2^{2n}}+\frac{q^{2}\left(p_{1}+p_{2}\right)}{2^{2n+1}}+\frac{2q^{3}}{2^{2n}}+\frac{q^{3/2}}{2^{n}}\\ \; & +\frac{4q{\left(p_{1}+p_{2}+2q\right)}^{2}}{2^{2n}}+\frac{3\sqrt{q}\left(p_{1}+p_{2}\right)}{2^{n}}+\frac{11q}{2^{n}}. \end{array}$$

Corollary 1 shows that the construction SoEM22 in (12) is secure roughly up to $p_{1}=p_{2}=q=O\left(2^{2n/3}\right)$ adversarial queries in the multi-key setting.

Similarly, given an $\epsilon_{1}$-regular and $\epsilon_{2}$-AXU keyed hash family H from T to ${\left\{0,1\right\}}^{n}$, one can obtain a good $\left(\epsilon_{1},\epsilon_{2}\right)$-key-derivation pair $\left(H_{K_{h}^{1}},H_{K_{h}^{2}}\right)$ for $\left(H_{K_{h}^{1}},H_{K_{h}^{2}}\right)\overset{\$}{\leftarrow }H^{2}$, and finally conclude the following corollary.

**Corollary** **2.** *Let n∈N, and H be an $\epsilon_{1}$-regular and $\epsilon_{2}$-AXU keyed hash family from T to ${\left\{0,1\right\}}^{n}$. Consider the tweakable function ${\mathrm{TPRF}}_{H_{K_{h}^{1}},H_{K_{h}^{2}}}^{P_{1},P_{2}}:T\times {\left\{0,1\right\}}^{n}\to {\left\{0,1\right\}}^{n}$ defined in (13) from two random permutations $P_{1},P_{2}\overset{\$}{\leftarrow }\mathrm{Perm}\left(n\right)$. For any deterministic distinguisher D making at most $p_{1}$ queries to $P_{1}$, $p_{2}$ queries to $P_{2}$, and q queries to ${\mathrm{TPRF}}_{H_{K_{h}^{1}},H_{K_{h}^{2}}}^{P_{1},P_{2}}$ or Φ such that $p_{1}+p_{2}+3q\leq 2^{n-1}$, we have*

(17) $$\begin{array}{cc} {\mathrm{Adv}}_{{\mathrm{TPRF}}_{H_{K_{h}^{1}},H_{K_{h}^{2}}}^{P_{1},P_{2}}}^{tweak}\left(D\right)\leq & 3qp_{1}p_{2}\epsilon_{1}^{2}+\frac{\epsilon_{1}\left(\epsilon_{2}q^{2}+2\sqrt{q}\right)\left(p_{1}+p_{2}\right)}{2}+2\epsilon_{2}^{2}q^{3}+\epsilon_{2}q^{3/2}\\ \; & +\frac{4q{\left(p_{1}+p_{2}+2q\right)}^{2}}{2^{2n}}+\frac{2\sqrt{q}\left(p_{1}+p_{2}\right)}{2^{n}}+\frac{11q}{2^{n}}. \end{array}$$

Assume that H is uniform (i.e., $2^{-n}$-regular) and XOR-universal (i.e., $2^{-n}$-AXU). Then Corollary 2 shows that ${\mathrm{TPRF}}_{H_{K_{h}^{1}},H_{K_{h}^{2}}}^{P_{1},P_{2}}$ in Equation (13) is secure roughly up to $p_{1}=p_{2}=q=O\left(2^{2n/3}\right)$ adversarial queries. This means that ${\mathrm{TPRF}}_{H_{K_{h}^{1}},H_{K_{h}^{2}}}^{P_{1},P_{2}}$ is a beyond-birthday secure tweakable PRF.

Finally, let M denote a message space. Given an $\epsilon_{1}$-regular and $\epsilon_{2}$-AXU keyed hash family H from M to ${\left\{0,1\right\}}^{n}$, we can construct a nonce based MAC (denoted by Sum2PMAC), from two random permutations $P_{1},P_{2}\overset{\$}{\leftarrow }\mathrm{Perm}\left(n\right)$ and H, as

(18) $$\begin{array}{c} T=P_{1}\left(N⊕H_{K_{h}^{1}}\left(M\right)\right)⊕P_{2}\left(N⊕H_{K_{h}^{2}}\left(M\right)\right)⊕H_{K_{h}^{1}}\left(M\right)⊕H_{K_{h}^{2}}\left(M\right), \end{array}$$

where $\left(H_{K_{h}^{1}},H_{K_{h}^{2}}\right)\overset{\$}{\leftarrow }H^{2}$, M∈M is message, and $N\in {\left\{0,1\right\}}^{n}$ is a nonce. Due to assumption of H, when we set T=M, then $\left(H_{K_{h}^{1}},H_{K_{h}^{2}}\right)$ is a good $\left(\epsilon_{1},\epsilon_{2}\right)$-key-derivation pair. Therefore, the following corollary holds.

**Corollary** **3.** *Let n∈N, and M be a message space. Let H be an $\epsilon_{1}$-regular and $\epsilon_{2}$-AXU keyed hash family from M to ${\left\{0,1\right\}}^{n}$. Consider the nonce based MAC Sum2PMAC defined in (18) from two random permutations $P_{1},P_{2}\overset{\$}{\leftarrow }\mathrm{Perm}\left(n\right)$. For any deterministic distinguisher D making at most $p_{1}$ queries to $P_{1}$, $p_{2}$ queries to $P_{2}$, and q evaluation queries, we have*

(19) $$\begin{array}{cc} {\mathrm{Adv}}_{\mathrm{Sum}2\mathrm{PMAC}}^{\mathrm{prf}}\left(D\right)\leq & 3qp_{1}p_{2}\epsilon_{1}^{2}+\frac{\epsilon_{1}\left(\epsilon_{2}q^{2}+2\sqrt{q}\right)\left(p_{1}+p_{2}\right)}{2}+2\epsilon_{2}^{2}q^{3}+\epsilon_{2}q^{3/2}\\ \; & +\frac{4q{\left(p_{1}+p_{2}+2q\right)}^{2}}{2^{2n}}+\frac{2\sqrt{q}\left(p_{1}+p_{2}\right)}{2^{n}}+\frac{11q}{2^{n}}. \end{array}$$

Assume that for any message M∈M, one has |M|<n·l for some integer l∈N. Then the keyed hash family from M to ${\left\{0,1\right\}}^{n}$ can be instantiated by the ${\mathrm{Poly}}_{H_{K_{h}}}$ defined in (10), which is $\frac{l}{2^{n}}$-regular and $\frac{l}{2^{n}}$-AXU. In this case, when one sets $p_{1}=p_{2}=q$, then ${\mathrm{Adv}}_{\mathrm{Sum}2\mathrm{PMAC}}^{\mathrm{prf}}\left(D\right)$ in (19) can be bounded as

$$\frac{\left(6l^{2}+64\right)q^{3}}{2^{2n}}+\frac{\left(3l+4\right)q^{3/2}}{2^{n}}+\frac{11q}{2^{n}}.$$

If *l* is a constant, then Sum2PMAC is a beyond-birthday secure MAC.

**Proof of Theorem** **1.** For convenience, we follow some notations in [16,17] in this proof. Let $\tau =(Q_{F},Q_{P_{1}},Q_{P_{2}},\left(f_{1},f_{2}\right))$ be an attainable transcript, where $|Q_{F}|=q$, $|Q_{P_{1}}|=p_{1}$, and $|Q_{P_{2}}|=p_{2}$. In addition, we write these sets more clearly as:

$$\begin{array}{cc} \; & Q_{F}=\left\{\left(t_{1},x_{1},y_{1}\right),\ldots ,\left(t_{q},x_{q},y_{q}\right)\right\},\\ \; & Q_{P_{1}}=\left\{\left(u_{1,1},v_{1,1}\right),\ldots ,\left(u_{1,p_{1}},v_{1,p_{1}}\right)\right\},\\ \; & Q_{P_{2}}=\left\{\left(u_{2,1},v_{2,1}\right),\ldots ,\left(u_{2,p_{2}},v_{2,p_{2}}\right)\right\}. \end{array}$$

We denote

$$\begin{array}{c} U_{1}=\left\{u_{1}\in {\left\{0,1\right\}}^{n}:\left(u_{1},v_{1}\right)\in Q_{P_{1}}\right\},\;\;V_{1}=\left\{v_{1}\in {\left\{0,1\right\}}^{n}:\left(u_{1},v_{1}\right)\in Q_{P_{1}}\right\}, \end{array}$$

and

$$\begin{array}{c} U_{2}=\left\{u_{2}\in {\left\{0,1\right\}}^{n}:\left(u_{2},v_{2}\right)\in Q_{P_{2}}\right\},\;\;\;V_{2}=\left\{v_{2}\in {\left\{0,1\right\}}^{n}:\left(u_{2},v_{2}\right)\in Q_{P_{2}}\right\}. \end{array}$$

For each $u\in {\left\{0,1\right\}}^{n}$, two associated sets can be defined as:

$$\begin{array}{cc} \; & X_{u}^{1}=\left\{\left(t,x,y\right)\in Q_{F}:x⊕f_{1}\left(t\right)=u\right\},\;\;X_{u}^{2}=\left\{\left(t,x,y\right)\in Q_{F}:x⊕f_{2}\left(t\right)=u\right\}. \end{array}$$

Now we define four parameters for transcript $\tau =(Q_{F},Q_{P_{1}},Q_{P_{2}},\left(f_{1},f_{2}\right))$ as

$$\begin{array}{cc} \; & \alpha_{1}\overset{def}{=}\left|\left\{\left(t,x,y\right)\in Q_{F}:x⊕f_{1}\left(t\right)\in U_{1}\right\}\right|,\\ \; & \alpha_{2}\overset{def}{=}\left|\left\{\left(t,x,y\right)\in Q_{F}:x⊕f_{2}\left(t\right)\in U_{2}\right\}\right|,\\ \; & \beta_{1}\overset{def}{=}\left|\left\{\left(t,x,y\right)\in Q_{F}:\exists \left(t,x,y\right)\neq \left(t^{\prime },x^{\prime },y^{\prime }\right),x⊕f_{1}\left(t\right)=x^{\prime }⊕f_{1}\left(t^{\prime }\right)\right\}\right|,\\ \; & \beta_{2}\overset{def}{=}\left|\left\{\left(t,x,y\right)\in Q_{F}:\exists \left(t,x,y\right)\neq \left(t^{\prime },x^{\prime },y^{\prime }\right),x⊕f_{2}\left(t\right)=x^{\prime }⊕f_{2}\left(t^{\prime }\right)\right\}\right|. \end{array}$$

$\beta_{1}$ and $\beta_{2}$ can be also expressed as

β1=∑x∈{0,1}n:δD1(x)>1δD1(x),β2=∑x∈{0,1}n:δD2(x)>1δD2(x),

where $D_{1}=\left\{x⊕f_{1}\left(t\right):\left(t,x,y\right)\in Q_{F}\right\}$ and $D_{2}=\left\{x⊕f_{2}\left(t\right):\left(t,x,y\right)\in Q_{F}\right\}$.

An attainable transcript $\tau =(Q_{F},Q_{P_{1}},Q_{P_{2}},\left(f_{1},f_{2}\right))$ is said bad if any one of the following conditions is satisfied:

- (B-1): $\exists i\in \left[q\right],j\in \left[p_{1}\right],j^{\prime }\in \left[p_{2}\right]$ for $\left(t_{i},x_{i},y_{i}\right)\in Q_{F}$, $u_{1,j}\in U_{1}$, and $u_{2,j^{\prime }}\in U_{2}$ such that $x_{i}⊕f_{1}\left(t_{i}\right)=u_{1,j}$ and $x_{i}⊕f_{2}\left(t_{i}\right)=u_{2,j^{\prime }}$.
- (B-2): $\exists i\in \left[q\right],j\in \left[p_{1}\right],j^{\prime }\in \left[p_{2}\right]$ for $\left(t_{i},x_{i},y_{i}\right)\in Q_{F}$, $\left(u_{1,j},v_{1,j}\right)\in Q_{P_{1}}$, and $v_{2,j^{\prime }}\in V_{2}$ such that $x_{i}⊕f_{1}\left(t_{i}\right)=u_{1,j}$ and $v_{1,j}⊕f_{1}\left(t_{i}\right)⊕f_{2}\left(t_{i}\right)⊕y_{i}=v_{2,j^{\prime }}$.
- (B-3): $\exists i\in \left[q\right],j\in \left[p_{1}\right],j^{\prime }\in \left[p_{2}\right]$ for $\left(t_{i},x_{i},y_{i}\right)\in Q_{F}$, $v_{1,j}\in V_{1}$, and $\left(u_{2,j^{\prime }},v_{2,j^{\prime }}\right)\in Q_{P_{2}}$ such that $x_{i}⊕f_{2}\left(t_{i}\right)=u_{2,j^{\prime }}$ and $v_{2,j^{\prime }}⊕f_{1}\left(t_{i}\right)⊕f_{2}\left(t_{i}\right)⊕y_{i}=v_{1,j}$.
- (B-4): $\exists i,i^{\prime }\in \left[q\right]$ for $\left(t_{i},x_{i},y_{i}\right)\neq \left(t_{i^{\prime }},x_{i^{\prime }},y_{i^{\prime }}\right)\in Q_{F}$ such that $x_{i}⊕f_{1}\left(t_{i}\right)=x_{i^{\prime }}⊕f_{1}\left(t_{i^{\prime }}\right)$ and $y_{i}⊕f_{1}\left(t_{i}\right)⊕f_{2}\left(t_{i}\right)=y_{i^{\prime }}⊕f_{1}\left(t_{i^{\prime }}\right)⊕f_{2}\left(t_{i^{\prime }}\right)$.
- (B-5): $\exists i,i^{\prime }\in \left[q\right]$ for $\left(t_{i},x_{i},y_{i}\right)\neq \left(t_{i^{\prime }},x_{i^{\prime }},y_{i^{\prime }}\right)\in Q_{F}$ such that $x_{i}⊕f_{2}\left(t_{i}\right)=x_{i^{\prime }}⊕f_{2}\left(t_{i^{\prime }}\right)$ and $y_{i}⊕f_{1}\left(t_{i}\right)⊕f_{2}\left(t_{i}\right)=y_{i^{\prime }}⊕f_{1}\left(t_{i^{\prime }}\right)⊕f_{2}\left(t_{i^{\prime }}\right)$.
- (B-6): $\exists i,i^{\prime }\in \left[q\right],j\in \left[p_{1}\right]$ for $\left(t_{i},x_{i},y_{i}\right)\neq \left(t_{i^{\prime }},x_{i^{\prime }},y_{i^{\prime }}\right)\in Q_{F}$, and $u_{1,j}\in U_{1}$ such that $x_{i}⊕f_{1}\left(t_{i}\right)=u_{1,j}$ and $x_{i}⊕f_{2}\left(t_{i}\right)=x_{i^{\prime }}⊕f_{2}\left(t_{i^{\prime }}\right)$.
- (B-7): $\exists i,i^{\prime }\in \left[q\right],j\in \left[p_{2}\right]$ for $\left(t_{i},x_{i},y_{i}\right)\neq \left(t_{i^{\prime }},x_{i^{\prime }},y_{i^{\prime }}\right)\in Q_{F}$, and $u_{2,j}\in U_{2}$ such that $x_{i}⊕f_{2}\left(t_{i}\right)=u_{2,j}$ and $x_{i}⊕f_{1}\left(t_{i}\right)=x_{i^{\prime }}⊕f_{1}\left(t_{i^{\prime }}\right)$.
- (B-8): $\exists i,i^{\prime },i^{\prime \prime }\in \left[q\right]$ for distinct tuples $\left(t_{i},x_{i},y_{i}\right),\left(t_{i^{\prime }},x_{i^{\prime }},y_{i^{\prime }}\right),\left(t_{i^{\prime \prime }},x_{i^{\prime \prime }},y_{i^{\prime \prime }}\right)\in Q_{F}$, such that $x_{i}⊕f_{1}\left(t_{i}\right)=x_{i^{\prime }}⊕f_{1}\left(t_{i^{\prime }}\right)$ and $x_{i}⊕f_{2}\left(t_{i}\right)=x_{i^{\prime \prime }}⊕f_{2}\left(t_{i^{\prime \prime }}\right)$.
- (B-9): $\exists i,i^{\prime }\in \left[q\right],j,j^{\prime }\in \left[p_{1}\right]$ for $\left(t_{i},x_{i},y_{i}\right),\left(t_{i^{\prime }},x_{i^{\prime }},y_{i^{\prime }}\right)\in Q_{F}$ and $\left(u_{1,j},v_{1,j}\right)$, $\left(u_{1,j^{\prime }},v_{1,j^{\prime }}\right)\in Q_{P_{1}}$ such that $x_{i}⊕f_{1}\left(t_{i}\right)=u_{1,j}$, $x_{i^{\prime }}⊕f_{1}\left(t_{i^{\prime }}\right)=u_{1,j^{\prime }}$, and $f_{1}\left(t_{i}\right)⊕f_{2}\left(t_{i}\right)⊕v_{1,j}⊕y_{i}=f_{1}\left(t_{i^{\prime }}\right)⊕f_{2}\left(t_{i^{\prime }}\right)⊕v_{1,j^{\prime }}⊕y_{i^{\prime }}$.
- (B-10): $\exists i,i^{\prime }\in \left[q\right],j,j^{\prime }\in \left[p_{2}\right]$ for $\left(t_{i},x_{i},y_{i}\right),\left(t_{i^{\prime }},x_{i^{\prime }},y_{i^{\prime }}\right)\in Q_{F}$ and $\left(u_{2,j},v_{2,j}\right)$, $\left(u_{2,j^{\prime }},v_{2,j^{\prime }}\right)\in Q_{P_{2}}$ such that $x_{i}⊕f_{2}\left(t_{i}\right)=u_{2,j}$, $x_{i^{\prime }}⊕f_{2}\left(t_{i^{\prime }}\right)=u_{2,j^{\prime }}$, and $f_{1}\left(t_{i}\right)⊕f_{2}\left(t_{i}\right)⊕v_{2,j}⊕y_{i}=f_{1}\left(t_{i^{\prime }}\right)⊕f_{2}\left(t_{i^{\prime }}\right)⊕v_{2,j^{\prime }}⊕y_{i^{\prime }}$.
- (B-11): $\alpha_{1}\geq \sqrt{q}$.
- (B-12): $\alpha_{2}\geq \sqrt{q}$.
- (B-13): $\beta_{1}\geq \sqrt{q}$ or $\beta_{2}\geq \sqrt{q}$.

Otherwise, we call τ a good transcript.

### 3.1. Analysis of Bad Transcripts

The proportion of all bad transcripts in the ideal world is upper bounded by the following lemma.

**Lemma** **3.** *Let $T_{\mathrm{id}}$ be the probability distribution of transcript $\tau =(Q_{F},Q_{P_{1}},Q_{P_{2}},\;(f_{1},f_{2}))$ in the ideal world, where $|Q_{P_{1}}|=p_{1}$, $|Q_{P_{2}}|=p_{2}$, $|Q_{F}|=q$, and $\left(f_{1},f_{2}\right)$ is a good $\left(\epsilon_{1},\epsilon_{2}\right)$-key-derivation pair. Then we have*

$$\begin{array}{c} \mathrm{Pr}\left[T_{\mathrm{id}}\in \Gamma_{\mathrm{bad}}\right]\leq 3qp_{1}p_{2}\epsilon_{1}^{2}+\frac{\epsilon_{1}\left(\epsilon_{2}q^{2}+2\sqrt{q}\right)\left(p_{1}+p_{2}\right)}{2}+2\epsilon_{2}^{2}q^{3}+\frac{q}{2^{n}}+\epsilon_{2}q^{3/2}. \end{array}$$

**Proof.**  Here we assume that there exists no repeated items in $Q_{P_{1}}$, $Q_{P_{2}}$, and $Q_{F}$ w.l.o.g. Then for each distinct construction query $\left(t,x,y\right)\in Q_{F}$, *y* is sampled uniformly and independently from ${\left\{0,1\right\}}^{n}$ in the ideal world. For each i∈[13], the set of all transcripts satisfying (B-*i*) is denoted by $\Gamma_{i}$. By union bound, one has

(20) $$\begin{array}{c} \mathrm{Pr}\left[T_{\mathrm{id}}\in \Gamma_{\mathrm{bad}}\right]\leq \sum_{i=1}^{13}\mathrm{Pr}\left[T_{\mathrm{id}}\in \Gamma_{i}\right]. \end{array}$$

For each i∈[13], the way to upper bound $\mathrm{Pr}[T_{\mathrm{id}}\in \Gamma_{i}]$ is similar to that in [16,17,22]. Hence, we give the details in Appendix A. By combining these upper bounds together, the proof of Lemma 3 is finished.  □

### 3.2. Analysis of Good Transcripts

In Lemma 4, we show that the probability of any good transcript τ in the real world is close to its probability in the ideal world.

**Lemma** **4.** *Let $T_{\mathrm{id}}$ be the probability distribution of transcripts in the ideal world, and $T_{\mathrm{re}}$ be the probability distribution in the real world. Then for any good transcript $\tau =(Q_{F},Q_{P_{1}},Q_{P_{2}}$, $\left(f_{1},f_{2})\right)$ with parameters $p_{1}$, $p_{2}$, and q satisfying $p_{1}+p_{2}+3q\leq 2^{n-1}$, one has*

$$\begin{array}{c} \frac{\mathrm{Pr}[T_{\mathrm{re}}=\tau ]}{\mathrm{Pr}[T_{\mathrm{id}}=\tau ]}\geq 1-\frac{4q{\left(p_{1}+p_{2}+2q\right)}^{2}}{2^{2n}}-\frac{2\sqrt{q}\left(p_{1}+p_{2}\right)}{2^{n}}-\frac{10q}{2^{n}}. \end{array}$$

**Proof.**  Given a good transcript τ, we define the following probability

$$p\left(\tau \right)\overset{\mathrm{def}}{=}\mathrm{Pr}\left[P_{1},P_{2}\overset{\$}{\leftarrow }\mathrm{Perm}\left(n\right):F_{f_{1},f_{2}}^{P_{1},P_{2}}⊢Q_{F}\;|\;P_{1}⊢Q_{P_{1}}\wedge P_{2}⊢Q_{P_{2}}\right].$$

By a simple combinatorial argument, we have

(21) $$\begin{array}{c} \frac{\mathrm{Pr}[T_{\mathrm{re}}=\tau ]}{\mathrm{Pr}[T_{\mathrm{id}}=\tau ]}=2^{nq}p\left(\tau \right). \end{array}$$

The next goal is to lower bound p(τ). For convenience, define five subsets of $Q_{F}$ as follows:

$$\begin{array}{c} Q_{U_{1}}=\left\{\left(t,x,y\right)\in Q_{F}:x⊕f_{1}\left(t\right)\in U_{1}\right\},\;\;Q_{U_{2}}=\left\{\left(t,x,y\right)\in Q_{F}:x⊕f_{2}\left(t\right)\in U_{2}\right\},\\ Q_{X_{1}}=\left\{\left(t,x,y\right)\in Q_{F}:\delta_{D_{1}}\left(x⊕f_{1}\left(t\right)\right)>1\;\mathrm{and}\;x⊕f_{1}\left(t\right)\notin U_{1}\right\},\\ Q_{X_{2}}=\left\{\left(t,x,y\right)\in Q_{F}:\delta_{D_{2}}\left(x⊕f_{2}\left(t\right)\right)>1\;\mathrm{and}\;x⊕f_{2}\left(t\right)\notin U_{2}\right\},\\ Q_{0}=\{\left(t,x,y\right)\in Q_{F}:\delta_{D_{1}}\left(x⊕h_{1}\left(t\right)\right)=\delta_{D_{2}}\left(x⊕f_{2}\left(t\right)\right)=1,\;x⊕f_{1}\left(t\right)\notin U_{1},\\ \;\;\;\;\;\;\;\mathrm{and}\;x⊕f_{2}\left(t\right)\notin U_{2}\}. \end{array}$$

Note that $|Q_{U_{1}}|=\alpha_{1}$ and $|Q_{U_{2}}|=\alpha_{2}$. The following proposition tells us that these sets form a partition of $Q_{F}$.

**Proposition** **1.** *Let $\tau \in \Gamma_{\mathrm{good}}$ be a good transcript. Then the sets $(Q_{U_{1}},Q_{U_{2}},Q_{X_{1}},$$Q_{X_{2}},Q_{0})$ defined above are pairwise disjoint.*

**Proof.**  By definition, we have $Q_{U_{1}}\cap Q_{X_{1}}=\emptyset$, $Q_{U_{2}}\cap Q_{X_{2}}=\emptyset$, and $Q_{U_{1}}\cap Q_{0}=Q_{U_{2}}\cap Q_{0}=Q_{X_{1}}\cap Q_{0}=Q_{X_{2}}\cap Q_{0}=\emptyset$. Since τ does not satisfy (B-1), we have $Q_{U_{1}}\cap Q_{U_{2}}=\emptyset$. Moreover, $Q_{U_{1}}\cap Q_{X_{2}}=\emptyset$ (resp. $Q_{U_{2}}\cap Q_{X_{1}}=\emptyset$) since τ does not satisfy (B-6) (resp. (B-7)). Finally, $Q_{X_{1}}\cap Q_{X_{2}}=\emptyset$ holds due to the fact $\tau \notin \Gamma_{8}$.  □

We use $E_{U_{1}}$, $E_{U_{2}}$, $E_{X_{1}}$, $E_{X_{2}}$, and $E_{0}$ to denote the events that $F_{f_{1},f_{2}}^{P_{1},P_{2}}⊢Q_{U_{1}},Q_{U_{2}},Q_{X_{1}},Q_{X_{2}}$, and $Q_{0}$, respectively. Then $F_{f_{1},f_{2}}^{P_{1},P_{2}}⊢Q_{F}$ is equivalent to $E_{U_{1}}\wedge E_{U_{2}}\wedge E_{X_{1}}\wedge E_{X_{2}}\wedge E_{0}$. Hence, it holds that

$$\begin{array}{cc} p(\tau ) & =\mathrm{Pr}[F_{f_{1},f_{2}}^{P_{1},P_{2}}⊢Q_{F}\;|\;P_{i}⊢Q_{P_{i}},i=1,2]\\ \; & =\mathrm{Pr}[E_{U_{1}}\wedge E_{U_{2}}\wedge E_{X_{1}}\wedge E_{X_{2}}\wedge E_{0}\;|\;P_{i}⊢Q_{P_{i}},i=1,2]\\ \; & =p^{\prime }\left(\tau \right)p^{\prime \prime }\left(\tau \right), \end{array}$$

where

$$p^{\prime }\left(\tau \right)=\mathrm{Pr}\left[E_{U_{1}}\wedge E_{U_{2}}\;|\;P_{i}⊢Q_{P_{i}},i=1,2\right],$$

and

$$p^{\prime \prime }\left(\tau \right)=\mathrm{Pr}\left[E_{X_{1}}\wedge E_{X_{2}}\wedge E_{0}\;|\;E_{U_{1}}\wedge E_{U_{2}}\wedge \left(P_{i}⊢Q_{P_{i}},i=1,2\right)\right].$$

The way to compute $p^{\prime }\left(\tau \right)$ and $p^{\prime \prime }\left(\tau \right)$, and the way to lower bound $\frac{\mathrm{Pr}[T_{\mathrm{re}}=\tau ]}{\mathrm{Pr}[T_{\mathrm{id}}=\tau ]}$ are similar to those in [16] so that we show the details in Appendix B.  □

Finally, by Lemmas 1, 3, and 4, Theorem 1 can be proved.  □

## 4. Multi-Key and Tweakable Secure PRFs from One Random Permutation

In this section, we first use one bidirectionally efficient random permutation $P\overset{\$}{\leftarrow }\mathrm{Perm}\left(n\right)$ to construct beyond-birthday and multi-key secure PRFs with a parallelizable structure as

(22) $$\begin{array}{c} F_{K_{1},K_{2}}^{P}\left(x\right)=P\left(x⊕K_{1}\right)⊕P^{-1}\left(x⊕K_{2}\right)⊕K_{1}⊕K_{2} \end{array}$$

where $\left(K_{1},K_{2}\right)\overset{\$}{\leftarrow }{\left\{0,1\right\}}^{2n}$ is the key and $x\in {\left\{0,1\right\}}^{n}$ is the input.

Let H be an $\epsilon_{1}$-regular and $\epsilon_{2}$-AXU keyed hash family from T to ${\left\{0,1\right\}}^{n}$. Then we can tweak the construction $F_{K_{1},K_{2}}^{P}$ in Equation (22) as

(23) $$\begin{array}{c} {\mathrm{TPRF}}_{H_{K_{h}^{1}},H_{K_{h}^{2}}}^{P}\left(t,x\right)=P\left(x⊕H_{K_{h}^{1}}\left(t\right)\right)⊕P^{-1}\left(x⊕H_{K_{h}^{2}}\left(t\right)\right)⊕H_{K_{h}^{1}}\left(t\right)⊕H_{K_{h}^{2}}\left(t\right), \end{array}$$

where $\left(H_{K_{h}^{1}},H_{K_{h}^{2}}\right)\overset{\$}{\leftarrow }H^{2}$, t∈T, and $x\in {\left\{0,1\right\}}^{n}$.

As mentioned before, one can simultaneously show that the above two constructions are beyond-birthday secure in the multi-key and the tweakable settings by proving the BBB security of the “unified”function,

(24) $$\begin{array}{c} F_{f_{1},f_{2}}^{P}\left(t,x\right)=P\left(x⊕f_{1}\left(t\right)\right)⊕P^{-1}\left(x⊕f_{2}\left(t\right)\right)⊕f_{1}\left(t\right)⊕f_{2}\left(t\right), \end{array}$$

where $\left(f_{1},f_{2}\right)\in \mathrm{Func}{\left(T,{\left\{0,1\right\}}^{n}\right)}^{2}$ is a good $\left(\epsilon_{1},\epsilon_{2}\right)$-key-derivation pair, $P\overset{\$}{\leftarrow }\mathrm{Perm}\left(n\right)$, t∈T, and $x\in {\left\{0,1\right\}}^{n}$.

**Theorem** **2.** *Assume that n≥6 and q≥64 are two positive integers. Let $\left(f_{1},f_{2}\right)\in \mathrm{Func}{\left(T,{\left\{0,1\right\}}^{n}\right)}^{2}$ be a good $\left(\epsilon_{1},\epsilon_{2}\right)$-key-derivation pair, and $P\overset{\$}{\leftarrow }\mathrm{Perm}\left(n\right)$ be a random permutation. Consider the function $F_{f_{1},f_{2}}^{P}:T\times {\left\{0,1\right\}}^{n}\to {\left\{0,1\right\}}^{n}$ defined in Equation (24). For any deterministic distinguisher D making at most p queries to P and q queries to the construction oracle $F_{f_{1},f_{2}}^{P}$ or Φ such that $p+2q+6\sqrt{q}\leq 2^{n-1}$, one has*

(25) $$\begin{array}{cc} {\mathrm{Adv}}_{F_{f_{1},f_{2}}^{P}}^{unify}\left(D\right)\leq & \left(3qp^{2}+2q^{2}p\right)\epsilon_{1}^{2}+2q^{3}\epsilon_{2}^{2}+2q^{2}p\epsilon_{1}\epsilon_{2}+q^{3/2}\epsilon_{2}+2p\sqrt{q}\epsilon_{1}+\frac{12q}{2^{2n/3}}\\ \; & +\frac{4q{\left(p+2q+6\sqrt{q}\right)}^{2}+q^{3}}{2^{2n}}+\frac{18q^{3/2}+6p\sqrt{q}+9q}{2^{n}}+\frac{16\sqrt{q}}{2^{n/3}}. \end{array}$$

Same to Corollary 1, the following corollary holds.

**Corollary** **4.** *Assume n≥6 and q≥64 are two positive integers. Let $P\overset{\$}{\leftarrow }\mathrm{Perm}\left(n\right)$ be an n-bit random permutation. Consider the keyed function $F_{K_{1},K_{2}}^{P}:{\left\{0,1\right\}}^{n}\to {\left\{0,1\right\}}^{n}$ defined in (22). For any deterministic distinguisher D making at most p queries to P and at most totally q queries to $F_{K_{1}^{1},K_{2}^{1}}^{P},\ldots ,F_{K_{1}^{m},K_{2}^{m}}^{P}$ (resp. m independent ideal random functions $\varphi_{1},\ldots ,\varphi_{m}$) satisfying $p+2q+6\sqrt{q}\leq 2^{n-1}$, we have*

(26) $$\begin{array}{cc} {\mathrm{Adv}}_{F_{K_{1},K_{2}}^{P}}^{mk}\left(D\right)\leq & \frac{4q{\left(p+2q+6\sqrt{q}\right)}^{2}}{2^{2n}}+\frac{3q^{3}+3qp^{2}+4pq^{2}}{2^{2n}}\\ \; & +\frac{19q^{3/2}+8p\sqrt{q}+9q}{2^{n}}+\frac{16\sqrt{q}}{2^{n/3}}+\frac{12q}{2^{2n/3}}. \end{array}$$

Similarly, given an $\epsilon_{1}$-regular and $\epsilon_{2}$-AXU keyed hash family H from T to ${\left\{0,1\right\}}^{n}$, the following corollary holds.

**Corollary** **5.** *Assume n≥6 and q≥64. Let H be an $\epsilon_{1}$-regular and $\epsilon_{2}$-AXU keyed hash family from T to ${\left\{0,1\right\}}^{n}$, and $P\overset{\$}{\leftarrow }\mathrm{Perm}\left(n\right)$ be an n-bit random permutation. Consider the tweakable function ${\mathrm{TPRF}}_{H_{K_{h}^{1}},H_{K_{h}^{2}}}^{P}:T\times {\left\{0,1\right\}}^{n}\to {\left\{0,1\right\}}^{n}$ defined in (23). For any deterministic distinguisher D making at most p queries to P and q queries to ${\mathrm{TPRF}}_{H_{K_{h}^{1}},H_{K_{h}^{2}}}^{P}$ or Φ such that $p+2q+6\sqrt{q}\leq 2^{n-1}$, we have*

(27) $$\begin{array}{cc} {\mathrm{Adv}}_{{\mathrm{TPRF}}_{H_{K_{h}^{1}},H_{K_{h}^{2}}}^{P}}^{tweak}\left(D\right)\leq & \left(3qp^{2}+2q^{2}p\right)\epsilon_{1}^{2}+2q^{3}\epsilon_{2}^{2}+2q^{2}p\epsilon_{1}\epsilon_{2}+q^{3/2}\epsilon_{2}+2p\sqrt{q}\epsilon_{1}+\frac{12q}{2^{2n/3}}\\ \; & +\frac{4q{\left(p+2q+6\sqrt{q}\right)}^{2}+q^{3}}{2^{2n}}+\frac{18q^{3/2}+6p\sqrt{q}+9q}{2^{n}}+\frac{16\sqrt{q}}{2^{n/3}}. \end{array}$$

Denote M as a message space. Let H be an $\epsilon_{1}$-regular and $\epsilon_{2}$-AXU keyed hash family from M to ${\left\{0,1\right\}}^{n}$. Then we can construct a nonce based MAC denoted by Sum1PMAC, from one random permutation $P\overset{\$}{\leftarrow }\mathrm{Perm}\left(n\right)$ as

(28) $$\begin{array}{c} T=P\left(N⊕H_{K_{h}^{1}}\left(M\right)\right)⊕P^{-1}\left(N⊕H_{K_{h}^{2}}\left(M\right)\right)⊕H_{K_{h}^{1}}\left(M\right)⊕H_{K_{h}^{2}}\left(M\right), \end{array}$$

where $\left(H_{K_{h}^{1}},H_{K_{h}^{2}}\right)\overset{\$}{\leftarrow }H^{2}$, M∈M is message, and $N\in {\left\{0,1\right\}}^{n}$ is a nonce. In this case, $\left(H_{K_{h}^{1}},H_{K_{h}^{2}}\right)$ is a good $\left(\epsilon_{1},\epsilon_{2}\right)$-key-derivation pair, and we can obtain the following corollary.

**Corollary** **6.** *Assume n≥3 and q≥64. Let H be an $\epsilon_{1}$-regular and $\epsilon_{2}$-AXU keyed hash family from M to ${\left\{0,1\right\}}^{n}$. Consider the nonce based MAC Sum1PMAC defined in (28) based on a random permutation $P\overset{\$}{\leftarrow }\mathrm{Perm}\left(n\right)$ and H. For any deterministic distinguisher D making at most p queries to P and q evaluation queries, we have*

(29) $$\begin{array}{cc} {\mathrm{Adv}}_{\mathrm{Sum}1\mathrm{PMAC}}^{\mathrm{prf}}\left(D\right)\leq & \left(3qp^{2}+2q^{2}p\right)\epsilon_{1}^{2}+2q^{3}\epsilon_{2}^{2}+2q^{2}p\epsilon_{1}\epsilon_{2}+q^{3/2}\epsilon_{2}+2p\sqrt{q}\epsilon_{1}+\frac{12q}{2^{2n/3}}\\ \; & +\frac{4q{\left(p+2q+6\sqrt{q}\right)}^{2}+q^{3}}{2^{2n}}+\frac{18q^{3/2}+6p\sqrt{q}+9q}{2^{n}}+\frac{16\sqrt{q}}{2^{n/3}}. \end{array}$$

Let M denote a message space, where for some l∈N, |M|<n·l holds for each message M∈M. Then, the keyed hash family from M to ${\left\{0,1\right\}}^{n}$ can be instantiated by the ${\mathrm{Poly}}_{H_{K_{h}}}$ defined in (10), which is $\frac{l}{2^{n}}$-regular and $\frac{l}{2^{n}}$-AXU. In this setting, when *l* is set to a constant, then Sum1PMAC is a beyond-birthday secure MAC.

**Proof of Theorem** **2.** In this proof, we follow some notations in [16,17] for convenience. Let $\bar{\tau }=\left({\bar{Q}}_{F},{\bar{Q}}_{P},\left(f_{1},f_{2}\right)\right)$ be an attainable transcript with $|{\bar{Q}}_{F}|=q$ and $|{\bar{Q}}_{P}|=p$. We write these sets more clearly as follows:

$$\begin{array}{cc} \; & {\bar{Q}}_{F}=\left\{\left(t_{1},x_{1},y_{1}\right),\ldots ,\left(t_{q},x_{q},y_{q}\right)\right\},\\ \; & {\bar{Q}}_{P}=\left\{\left(u_{1},v_{1}\right),\ldots ,\left(u_{p},v_{p}\right)\right\}. \end{array}$$

We also denote

$$\begin{array}{c} U=\left\{u_{1}\in {\left\{0,1\right\}}^{n}:\left(u_{1},v_{1}\right)\in {\bar{Q}}_{P}\right\}\;\mathrm{and}\;V=\left\{v_{1}\in {\left\{0,1\right\}}^{n}:\left(u_{1},v_{1}\right)\in {\bar{Q}}_{P}\right\} \end{array}$$

as domain and range of ${\bar{Q}}_{P}$ respectively. For each $u\in {\left\{0,1\right\}}^{n}$, two associated sets can be defined as:

$$\begin{array}{cc} \; & {\bar{X}}_{u}^{1}=\left\{\left(t,x,y\right)\in {\bar{Q}}_{F}:x⊕f_{1}\left(t\right)=u\right\}\;\mathrm{and}\;{\bar{X}}_{u}^{2}=\left\{\left(t,x,y\right)\in {\bar{Q}}_{F}:x⊕f_{2}\left(t\right)=u\right\}. \end{array}$$

Now we define four parameters for transcript $\bar{\tau }=\left({\bar{Q}}_{F},{\bar{Q}}_{P},\left(f_{1},f_{2}\right)\right)$ as

$$\begin{array}{cc} \; & {\bar{\alpha }}_{1}\overset{def}{=}\left|\left\{\left(t,x,y\right)\in {\bar{Q}}_{F}:x⊕f_{1}\left(t\right)\in U\right\}\right|,\\ \; & {\bar{\alpha }}_{2}\overset{def}{=}\left|\left\{\left(t,x,y\right)\in {\bar{Q}}_{F}:x⊕f_{2}\left(t\right)\in V\right\}\right|,\\ \; & {\bar{\beta }}_{1}\overset{def}{=}\left|\left\{\left(t,x,y\right)\in {\bar{Q}}_{F}:\exists \left(t,x,y\right)\neq \left(t^{\prime },x^{\prime },y^{\prime }\right),x⊕f_{1}\left(t\right)=x^{\prime }⊕f_{1}\left(t^{\prime }\right)\right\}\right|,\\ \; & {\bar{\beta }}_{2}\overset{def}{=}\left|\left\{\left(t,x,y\right)\in {\bar{Q}}_{F}:\exists \left(t,x,y\right)\neq \left(t^{\prime },x^{\prime },y^{\prime }\right),x⊕f_{2}\left(t\right)=x^{\prime }⊕f_{2}\left(t^{\prime }\right)\right\}\right|, \end{array}$$

where ${\bar{\beta }}_{1}$ and ${\bar{\beta }}_{2}$ can be also expressed as

β¯1=∑x∈{0,1}n:δD¯1(x)>1δD¯1(x)andβ¯2=∑x∈{0,1}n:δD¯2(x)>1δD¯2(x),

where ${\bar{D}}_{1}=\left\{x⊕f_{1}\left(t\right):\left(t,x,y\right)\in {\bar{Q}}_{F}\right\}$ and ${\bar{D}}_{2}=\left\{x⊕f_{2}\left(t\right):\left(t,x,y\right)\in {\bar{Q}}_{F}\right\}$.

An attainable transcript $\bar{\tau }=\left({\bar{Q}}_{F},{\bar{Q}}_{P},\left(f_{1},f_{2}\right)\right)$ is said bad if any one of the following conditions is satisfied:

- (C-1): ∃i∈[q] and $j,j^{\prime }\in \left[p\right]$ for $\left(t_{i},x_{i},y_{i}\right)\in {\bar{Q}}_{F}$, $u_{j}\in U$, and $v_{j^{\prime }}\in V$ such that $x_{i}⊕f_{1}\left(t_{i}\right)=u_{j}$ and $x_{i}⊕f_{2}\left(t_{i}\right)=v_{j^{\prime }}$.
- (C-2): ∃i∈[q] and $j,j^{\prime }\in \left[p\right]$ for $\left(t_{i},x_{i},y_{i}\right)\in {\bar{Q}}_{F}$, $\left(u_{j},v_{j}\right)\in {\bar{Q}}_{P}$, and $u_{j^{\prime }}\in U$ such that $x_{i}⊕f_{1}\left(t_{i}\right)=u_{j}$ and $v_{j}⊕f_{1}\left(t_{i}\right)⊕f_{2}\left(t_{i}\right)⊕y_{i}=u_{j^{\prime }}$.
- (C-3): ∃i∈[q] and $j,j^{\prime }\in \left[p\right]$ for $\left(t_{i},x_{i},y_{i}\right)\in {\bar{Q}}_{F}$, $\left(u_{j},v_{j}\right)\in {\bar{Q}}_{P}$, and $v_{j^{\prime }}\in V$ such that $x_{i}⊕f_{2}\left(t_{i}\right)=v_{j}$ and $u_{j}⊕f_{1}\left(t_{i}\right)⊕f_{2}\left(t_{i}\right)⊕y_{i}=v_{j^{\prime }}$.
- (C-4): $\exists i,i^{\prime }\in \left[q\right]$ and j∈[p] for $\left(t_{i},x_{i},y_{i}\right)\neq \left(t_{i^{\prime }},x_{i^{\prime }},y_{i^{\prime }}\right)\in {\bar{Q}}_{F}$ and $\left(u_{j},v_{j}\right)\in {\bar{Q}}_{P}$ such that $x_{i}⊕f_{1}\left(t_{i}\right)=u_{j}$ and $v_{j}⊕f_{1}\left(t_{i}\right)⊕f_{2}\left(t_{i}\right)⊕y_{i}=x_{i^{\prime }}⊕f_{1}\left(t_{i^{\prime }}\right)$.
- (C-5): $\exists i,i^{\prime }\in \left[q\right]$ and j∈[p] for $\left(t_{i},x_{i},y_{i}\right)\neq \left(t_{i^{\prime }},x_{i^{\prime }},y_{i^{\prime }}\right)\in {\bar{Q}}_{F}$ and $\left(u_{j},v_{j}\right)\in {\bar{Q}}_{P}$ such that $x_{i}⊕f_{2}\left(t_{i}\right)=v_{j}$ and $u_{j}⊕f_{1}\left(t_{i}\right)⊕f_{2}\left(t_{i}\right)⊕y_{i}=x_{i^{\prime }}⊕f_{2}\left(t_{i^{\prime }}\right)$.
- (C-6): $\exists i,i^{\prime }\in \left[q\right]$ and j∈[p] for $\left(t_{i},x_{i},y_{i}\right)\neq \left(t_{i^{\prime }},x_{i^{\prime }},y_{i^{\prime }}\right)\in {\bar{Q}}_{F}$ and $u_{j}\in U$ such that $x_{i}⊕f_{1}\left(t_{i}\right)=u_{j}$ and $x_{i}⊕f_{2}\left(t_{i}\right)=x_{i^{\prime }}⊕f_{2}\left(t_{i^{\prime }}\right)$.
- (C-7): $\exists i,i^{\prime }\in \left[q\right]$ and j∈[p] for $\left(t_{i},x_{i},y_{i}\right)\neq \left(t_{i^{\prime }},x_{i^{\prime }},y_{i^{\prime }}\right)\in {\bar{Q}}_{F}$ and $v_{j}\in V$ such that $x_{i}⊕f_{2}\left(t_{i}\right)=v_{j}$ and $x_{i}⊕f_{1}\left(t_{i}\right)=x_{i^{\prime }}⊕f_{1}\left(t_{i^{\prime }}\right)$.
- (C-8): $\exists i,i^{\prime }\in \left[q\right]$ for $\left(t_{i},x_{i},y_{i}\right)\neq \left(t_{i^{\prime }},x_{i^{\prime }},y_{i^{\prime }}\right)\in {\bar{Q}}_{F}$ such that $x_{i}⊕f_{1}\left(t_{i}\right)=x_{i^{\prime }}⊕f_{1}\left(t_{i^{\prime }}\right)$ and $f_{1}\left(t_{i}\right)⊕f_{2}\left(t_{i}\right)⊕y_{i}=f_{1}\left(t_{i^{\prime }}\right)⊕f_{2}\left(t_{i^{\prime }}\right)⊕y_{i^{\prime }}$.
- (C-9): $\exists i,i^{\prime }\in \left[q\right]$ for $\left(t_{i},x_{i},y_{i}\right)\neq \left(t_{i^{\prime }},x_{i^{\prime }},y_{i^{\prime }}\right)\in {\bar{Q}}_{F}$ such that $x_{i}⊕f_{2}\left(t_{i}\right)=x_{i^{\prime }}⊕f_{2}\left(t_{i^{\prime }}\right)$ and $f_{1}\left(t_{i}\right)⊕f_{2}\left(t_{i}\right)⊕y_{i}=f_{1}\left(t_{i^{\prime }}\right)⊕f_{2}\left(t_{i^{\prime }}\right)⊕y_{i^{\prime }}$.
- (C-10):$\exists i,i^{\prime },$ and $i^{\prime \prime }\in \left[q\right]$ for pairwise distinct $\left(t_{i},x_{i},y_{i}\right),\left(t_{i^{\prime }},x_{i^{\prime }},y_{i^{\prime }}\right),$ and $\left(t_{i^{\prime \prime }},x_{i^{\prime \prime }},y_{i^{\prime \prime }}\right)\in {\bar{Q}}_{F}$ such that $x_{i}⊕f_{1}\left(t_{i}\right)=x_{i^{\prime }}⊕f_{1}\left(t_{i^{\prime }}\right)$ and $x_{i}⊕f_{2}\left(t_{i}\right)=x_{i^{\prime \prime }}⊕f_{2}\left(t_{i^{\prime \prime }}\right)$.
- (C-11): $\exists i,i^{\prime }\in \left[p\right]$ and $j,j^{\prime }\in \left[p\right]$ for $\left(t_{i},x_{i},y_{i}\right)\neq \left(t_{i^{\prime }},x_{i^{\prime }},y_{i^{\prime }}\right)\in {\bar{Q}}_{F}$ and $\left(u_{j},v_{j}\right)$,$\left(u_{j^{\prime }},v_{j^{\prime }}\right)\in {\bar{Q}}_{P}$ such that $x_{i}⊕f_{1}\left(t_{i}\right)=u_{j}$, $x_{i^{\prime }}⊕f_{1}\left(t_{i^{\prime }}\right)=u_{j^{\prime }}$ and $v_{j}⊕f_{1}\left(t_{i}\right)⊕f_{2}\left(t_{i}\right)⊕y_{i}=v_{j^{\prime }}⊕f_{1}\left(t_{i^{\prime }}\right)⊕f_{2}\left(t_{i^{\prime }}\right)⊕y_{i^{\prime }}$.
- (C-12): $\exists i,i^{\prime }\in \left[p\right]$ and $j,j^{\prime }\in \left[p\right]$ for $\left(t_{i},x_{i},y_{i}\right)\neq \left(t_{i^{\prime }},x_{i^{\prime }},y_{i^{\prime }}\right)\in {\bar{Q}}_{F}$ and $\left(u_{j},v_{j}\right)$,$\left(u_{j^{\prime }},v_{j^{\prime }}\right)\in {\bar{Q}}_{P}$ such that $x_{i}⊕f_{2}\left(t_{i}\right)=v_{j}$, $x_{i^{\prime }}⊕f_{2}\left(t_{i^{\prime }}\right)=v_{j^{\prime }}$ and $u_{j}⊕f_{1}\left(t_{i}\right)⊕f_{2}\left(t_{i}\right)⊕y_{i}=u_{j^{\prime }}⊕f_{1}\left(t_{i^{\prime }}\right)⊕f_{2}\left(t_{i^{\prime }}\right)⊕y_{i^{\prime }}$.
- (C-13): ${\bar{\alpha }}_{1}\geq \sqrt{q}$.
- (C-14): ${\bar{\alpha }}_{2}\geq \sqrt{q}$.
- (C-15): ${\bar{\beta }}_{1}\geq \sqrt{q}$ or ${\bar{\beta }}_{2}\geq \sqrt{q}$.
- (C-16): $\exists i,i^{\prime },$ and $i^{\prime \prime }\in \left[q\right]$ for pairwise distinct $\left(t_{i},x_{i},y_{i}\right),\left(t_{i^{\prime }},x_{i^{\prime }},y_{i^{\prime }}\right),$ and $\left(t_{i^{\prime \prime }},x_{i^{\prime \prime }},y_{i^{\prime \prime }}\right)\in {\bar{Q}}_{F}$ such that $f_{1}\left(t_{i}\right)⊕f_{2}\left(t_{i}\right)⊕y_{i}=f_{1}\left(t_{i^{\prime }}\right)⊕f_{2}\left(t_{i^{\prime }}\right)⊕y_{i^{\prime }}$ and $f_{1}\left(t_{i}\right)⊕f_{2}\left(t_{i}\right)⊕y_{i}=f_{1}\left(t_{i^{\prime \prime }}\right)⊕f_{2}\left(t_{i^{\prime \prime }}\right)⊕y_{i^{\prime \prime }}$.
- (C-17): For sets ${\bar{Q}}_{0}=\left\{\left(t,x,y\right)\in {\bar{Q}}_{F}:\delta_{{\bar{D}}_{1}}\left(x⊕f_{1}\left(t\right)\right)=\delta_{{\bar{D}}_{2}}\left(x⊕f_{2}\left(t\right)\right)=1,x⊕f_{1}\left(t\right)\notin U,x⊕f_{2}\left(t\right)\notin V\right\}$, $\hat{U}=U\cup \left\{v⊕f_{1}\left(t\right)⊕f_{2}\left(t\right)⊕y:\left(t,x,y\right)\in {\bar{Q}}_{F},\left(u,v\right)\in {\bar{Q}}_{P},x⊕f_{1}\left(t\right)=u\in U\right\}\;\cup \left\{x⊕f_{1}\left(t\right):\left(t,x,y\right)\in Q_{F},x⊕f_{1}\left(t\right)\notin U\right\}$, and $\hat{V}=V\cup \left\{u⊕f_{1}\left(t\right)⊕f_{2}\left(t\right)⊕y:\left(t,x,y\right)\in {\bar{Q}}_{F},\left(u,v\right)\in {\bar{Q}}_{P},x⊕f_{2}\left(t\right)=v\in V\right\}\;\cup \left\{x⊕f_{2}\left(t\right):\left(t,x,y\right)\in {\bar{Q}}_{F},x⊕f_{2}\left(t\right)\notin V\right\}$ derived from the transcript, $D_{\hat{U}}\overset{def}{=}\left|\left\{x_{i}⊕f_{2}\left(t_{i}\right)⊕f_{1}\left(t_{i^{\prime }}\right)⊕f_{2}\left(t_{i^{\prime }}\right)⊕y_{i^{\prime }}\in \hat{U}:\left(t_{i},x_{i},y_{i}\right)\neq \left(t_{i^{\prime }},x_{i^{\prime }},y_{i^{\prime }}\right)\in {\bar{Q}}_{0}\right\}\right|\geq q^{3/2}$ or $D_{\hat{V}}\overset{def}{=}\left|\left\{x_{i}⊕f_{1}\left(t_{i}\right)⊕f_{1}\left(t_{i^{\prime }}\right)⊕f_{2}\left(t_{i^{\prime }}\right)⊕y_{i^{\prime }}\in \hat{V}:\left(t_{i},x_{i},y_{i}\right)\neq \left(t_{i^{\prime }},x_{i^{\prime }},y_{i^{\prime }}\right)\in {\bar{Q}}_{0}\right\}\right|\geq q^{3/2}$.

Otherwise, τ is said a good transcript.

### 4.1. Analysis of Bad Transcripts

Let $\Gamma_{i}^{\prime }$ be the set of all transcripts satisfying (C-*i*) for i∈[17]. The proportion of all bad transcripts in the ideal world can be upper bounded in the following lemma.

**Lemma** **5.** *Let $T_{\mathrm{id}}$ be the probability distribution of transcript $\bar{\tau }=({\bar{Q}}_{F},{\bar{Q}}_{P},\;(f_{1},f_{2}))$ in the ideal world, where $|{\bar{Q}}_{P}|=p$, $|{\bar{Q}}_{F}|=q$, and $\left(f_{1},f_{2}\right)$ is a good $\left(\epsilon_{1},\epsilon_{2}\right)$-key-derivation pair. Then we have*

$$\begin{array}{cc} \mathrm{Pr}[T_{\mathrm{id}}\in \Gamma_{\mathrm{bad}}]\leq & \left(3qp^{2}+2q^{2}p\right)\epsilon_{1}^{2}+2q^{3}\epsilon_{2}^{2}+2q^{2}p\epsilon_{1}\epsilon_{2}+q^{3/2}\epsilon_{2}\\ \; & +2p\sqrt{q}\epsilon_{1}+\frac{q+2\sqrt{q}\left(p+q\right)}{2^{n}}+\frac{q^{3}}{2^{2n}}. \end{array}$$

**Proof.**  Let $T_{\mathrm{id}}=\left({\bar{Q}}_{F},{\bar{Q}}_{P},\left(f_{1},f_{2}\right)\right)$ be any attainable transcript in the ideal world, where ${\bar{Q}}_{P}$ includes *p* permutation pairs from the interaction between distinguisher *D* and *P*. For each distinct construction query $\left(t,x,y\right)\in {\bar{Q}}_{F}$, *y* is sampled uniformly and independently from ${\left\{0,1\right\}}^{n}$. Without loss of generality, we assume that there exists no repeated items in ${\bar{Q}}_{F}$ and ${\bar{Q}}_{P}$.

The probabilities of $T_{\mathrm{id}}$ in $\Gamma_{\mathrm{bad}}$ can be upper bounded as

(30) $$\mathrm{Pr}\left[T_{\mathrm{id}}\in \Gamma_{\mathrm{bad}}\right]\leq \underset{{\mathrm{Bad}}_{M_{1}}}{\underset{︸}{\sum_{i=1}^{15}\mathrm{Pr}\left[T_{\mathrm{id}}\in \Gamma_{i}^{\prime }\right]}}+\underset{{\mathrm{Bad}}_{M_{2}}}{\underset{︸}{\mathrm{Pr}\left[T_{\mathrm{id}}\in \Gamma_{16}^{\prime }\right]+\mathrm{Pr}\left[T_{\mathrm{id}}\in \Gamma_{17}^{\prime }\right]}}.$$

For ${\mathrm{Bad}}_{M_{1}}$, one can obtain the following upper bound

(31) $$\begin{array}{c} {\mathrm{Bad}}_{M_{1}}\leq \left(3qp^{2}+2q^{2}p\right)\epsilon_{1}^{2}+2q^{3}\epsilon_{2}^{2}+2q^{2}p\epsilon_{1}\epsilon_{2}+2p\sqrt{q}\epsilon_{1}+q^{3/2}\epsilon_{2}+\frac{q}{2^{n}}, \end{array}$$

and more details can be found in Appendix C.

For ${\mathrm{Bad}}_{M_{2}}$, we need to study (C-16) and (C-17), respectively.

Bounding (C-16): For any three distinct construction queries $\left(t_{i},x_{i},y_{i}\right)$, $\left(t_{i^{\prime }},x_{i^{\prime }},y_{i^{\prime }}\right)$, and $\left(t_{i^{\prime \prime }},x_{i^{\prime \prime }},y_{i^{\prime \prime }}\right)\in {\bar{Q}}_{F}$, $y_{i^{\prime }}$ and $y_{i^{\prime \prime }}$ are independently and uniformly sampled from ${\left\{0,1\right\}}^{n}$. Hence, we have

$$\begin{array}{cc} \mathrm{Pr} & [\left(f_{1}\left(t_{i}\right)⊕f_{2}\left(t_{i}\right)⊕y_{i}=f_{1}\left(t_{i^{\prime }}\right)⊕f_{2}\left(t_{i^{\prime }}\right)⊕y_{i^{\prime }}\right)\wedge \\ \; & \left(f_{1}\left(t_{i}\right)⊕f_{2}\left(t_{i}\right)⊕y_{i}=f_{1}\left(t_{i^{\prime \prime }}\right)⊕f_{2}\left(t_{i^{\prime \prime }}\right)⊕y_{i^{\prime \prime }}\right)]\leq \frac{1}{2^{2n}}. \end{array}$$

Since the number of all possible tuples for $\left(\left(t_{i},x_{i},y_{i}\right),\left(t_{i^{\prime }},x_{i^{\prime }},y_{i^{\prime }}\right),\left(t_{i^{\prime \prime }},x_{i^{\prime \prime }},y_{i^{\prime \prime }}\right)\right)\in {\bar{Q}}_{F}\times {\bar{Q}}_{F}\times {\bar{Q}}_{F}$ is at most $q^{3}$, by union bound, one has

$$\mathrm{Pr}\left[T_{\mathrm{id}}\in \Gamma_{16}^{\prime }\right]\leq \frac{q^{3}}{2^{2n}}.$$

Bounding (C-17): First, we have $\left\{\left(t,x,y\right)\in {\bar{Q}}_{F}:x⊕f_{1}\left(t\right)\in U\right\}\cap \left\{\left(t,x,y\right)\in {\bar{Q}}_{F}:x⊕f_{1}\left(t\right)\notin U\right\}=\emptyset$ (which means $|\hat{U}|\leq p+q$). Hence, by the definition of ${\bar{Q}}_{0}$, it holds that $\left\{\left(t,x,y\right)\in {\bar{Q}}_{F}:x⊕f_{1}\left(t\right)\in U\right\}\cap {\bar{Q}}_{0}=\emptyset$. Similarly, we also have $\left\{\left(t,x,y\right)\in {\bar{Q}}_{F}:x⊕f_{2}\left(t\right)\in V\right\}\cap \left\{\left(t,x,y\right)\in {\bar{Q}}_{F}:x⊕f_{2}\left(t\right)\notin V\right\}=\emptyset$ (which means $|\hat{V}|\leq p+q$) and $\left\{\left(t,x,y\right)\in {\bar{Q}}_{F}:x⊕f_{2}\left(t\right)\in V\right\}\cap {\bar{Q}}_{0}=\emptyset$. By combing these facts and the definitions of $\hat{U}$, $\hat{V}$, and ${\bar{Q}}_{0}$, the random value *y* for each $\left(t,x,y\right)\in {\bar{Q}}_{0}$ in the ideal world is independent of any elements in $\hat{U}$ and $\hat{V}$. Therefore, for each pair $\left(\left(t_{i},x_{i},y_{i}\right),\left(t_{i^{\prime }},x_{i^{\prime }},y_{i^{\prime }}\right)\right)\in {\bar{Q}}_{0}\times {\bar{Q}}_{0}$, one has

$$\mathrm{Pr}\left[x_{i}⊕f_{2}\left(t_{i}\right)⊕f_{1}\left(t_{i^{\prime }}\right)⊕f_{2}\left(t_{i^{\prime }}\right)⊕y_{i^{\prime }}\in \hat{U}\right]\leq \frac{|\hat{U}|}{2^{n}}\leq \frac{p+q}{2^{n}}.$$

Then the expectation value of random variable $D_{\hat{U}}$ can be bounded as

E[DU^]≤∑((ti,xi,yi),(ti′,xi′,yi′))∈Q¯02:Pr[xi⊕f2(ti)⊕f1(ti′)⊕f2(ti′)⊕yi′∈U^]≤|Q¯0|2(p+q)2n≤q2(p+q)2n.

By Markov’s inequality, we have

$$\mathrm{Pr}\left[D_{\hat{U}}\geq q^{3/2}\right]\leq \frac{E[D_{\hat{U}}]}{q^{3/2}}\leq \frac{\sqrt{q}\left(p+q\right)}{2^{n}}.$$

Similarly, it holds that

$$\mathrm{Pr}\left[D_{\hat{V}}\geq q^{3/2}\right]\leq \frac{\sqrt{q}\left(p+q\right)}{2^{n}}.$$

Therefore, one has

$$\mathrm{Pr}\left[T_{\mathrm{id}}\in \Gamma_{17}^{\prime }\right]\leq \frac{2\sqrt{q}\left(p+q\right)}{2^{n}}.$$

Finally, by combining the upper bounds on ${\mathrm{Bad}}_{M_{1}}$ and ${\mathrm{Bad}}_{M_{2}}$ together, by (30), the proof of Lemma 5 is finished.  □

### 4.2. Analysis of Good Transcripts

In this part, we prove that for any good transcript $\bar{\tau }$, the probability to sample it in the real world is close to that in the ideal world, and this result can be formally stated in the following lemma.

**Lemma** **6.** *Assume that n≥6 and q≥64. Let $T_{\mathrm{id}}$ be the probability distribution of transcripts in the ideal world, and $T_{\mathrm{re}}$ be in the real world. Then for any good transcript $\bar{\tau }=\left({\bar{Q}}_{F},{\bar{Q}}_{P},\left(f_{1},f_{2}\right)\right)\in \Gamma_{\mathrm{good}}$ with parameters p and q satisfying $p+2q+6\sqrt{q}\leq 2^{n-1}$, one has*

$$\begin{array}{c} \frac{\mathrm{Pr}[T_{\mathrm{re}}=\bar{\tau }]}{\mathrm{Pr}[T_{\mathrm{id}}=\bar{\tau }]}\geq 1-\epsilon , \end{array}$$

*where $\epsilon =\frac{4q{\left(p+2q+6\sqrt{q}\right)}^{2}}{2^{2n}}+\frac{16q^{3/2}+4p\sqrt{q}+8q}{2^{n}}+\frac{16\sqrt{q}}{2^{n/3}}+\frac{12q}{2^{2n/3}}$.*

**Proof.**  Given a good transcript $\bar{\tau }$, we define the following probability

$$p\left(\bar{\tau }\right)\overset{\mathrm{def}}{=}\mathrm{Pr}\left[P\overset{\$}{\leftarrow }\mathrm{Perm}\left(n\right):F_{f_{1},f_{2}}^{P}⊢{\bar{Q}}_{F}\;|\;P⊢{\bar{Q}}_{P}\right].$$

By a simple combinatorial argument, it holds that

$$\frac{\mathrm{Pr}[T_{\mathrm{re}}=\bar{\tau }]}{\mathrm{Pr}[T_{\mathrm{id}}=\bar{\tau }]}=2^{nq}p\left(\bar{\tau }\right).$$

We first introduce some subsets of ${\bar{Q}}_{F}$ as follows:

$$\begin{array}{cc} \; & {\bar{Q}}_{U}=\left\{\left(t,x,y\right)\in {\bar{Q}}_{F}:x⊕f_{1}\left(t\right)\in U\right\},\;\;{\bar{Q}}_{V}=\left\{\left(t,x,y\right)\in {\bar{Q}}_{F}:x⊕f_{2}\left(t\right)\in V\right\},\\ \; & {\bar{Q}}_{X_{1}}=\left\{\left(t,x,y\right)\in {\bar{Q}}_{F}:\delta_{{\bar{D}}_{1}}\left(x⊕f_{1}\left(t\right)\right)>1\;\mathrm{and}\;x⊕f_{1}\left(t\right)\notin U\right\},\\ \; & {\bar{Q}}_{X_{2}}=\left\{\left(t,x,y\right)\in {\bar{Q}}_{F}:\delta_{{\bar{D}}_{2}}\left(x⊕f_{2}\left(t\right)\right)>1\;\mathrm{and}\;x⊕f_{2}\left(t\right)\notin V\right\},\\ \; & {\bar{Q}}_{0}=\{\left(t,x,y\right)\in {\bar{Q}}_{F}:\delta_{{\bar{D}}_{1}}\left(x⊕f_{1}\left(t\right)\right)=\delta_{{\bar{D}}_{2}}\left(x⊕f_{2}\left(t\right)\right)=1,\\ \; & \;\;\;x⊕f_{1}\left(t\right)\notin U,\;\mathrm{and}\;x⊕f_{2}\left(t\right)\notin V\}. \end{array}$$

Note that $|{\bar{Q}}_{U}|={\bar{\alpha }}_{1}$, $|{\bar{Q}}_{V}|={\bar{\alpha }}_{2}$, and ${\bar{Q}}_{0}$ has been defined in (C-17). In fact, these sets form a partition of ${\bar{Q}}_{F}$.

**Proposition** **2.** *Let $\bar{\tau }\in \Gamma_{\mathrm{good}}$ be a good transcript. Then $({\bar{Q}}_{U},{\bar{Q}}_{V},{\bar{Q}}_{X_{1}},{\bar{Q}}_{X_{2}},{\bar{Q}}_{0})$ defined above are pairwise disjoint.*

**Proof.**  By the definition of these five subsets, it holds that ${\bar{Q}}_{U}\cap {\bar{Q}}_{X_{1}}=\emptyset$, ${\bar{Q}}_{V}\cap {\bar{Q}}_{X_{2}}=\emptyset$, and ${\bar{Q}}_{U}\cap {\bar{Q}}_{0}={\bar{Q}}_{V}\cap {\bar{Q}}_{0}={\bar{Q}}_{X_{1}}\cap {\bar{Q}}_{0}={\bar{Q}}_{X_{2}}\cap {\bar{Q}}_{0}=\emptyset$. Since $\bar{\tau }$ does not satisfy (C-1), one has ${\bar{Q}}_{U}\cap {\bar{Q}}_{V}=\emptyset$. Besides, ${\bar{Q}}_{U}\cap {\bar{Q}}_{X_{2}}=\emptyset$ (resp. ${\bar{Q}}_{V}\cap {\bar{Q}}_{X_{1}}=\emptyset$) holds since $\bar{\tau }$ does not satisfy (C-6) (resp. (C-7)). Finally, ${\bar{Q}}_{X_{1}}\cap {\bar{Q}}_{X_{2}}=\emptyset$ since $\bar{\tau }\notin \Gamma_{10}^{\prime }$.   □

We use ${\bar{E}}_{U}$, ${\bar{E}}_{V}$, ${\bar{E}}_{X_{1}}$, ${\bar{E}}_{X_{2}}$, and ${\bar{E}}_{0}$ to denote the events $F_{f_{1},f_{2}}^{P}⊢{\bar{Q}}_{U},$${\bar{Q}}_{V},{\bar{Q}}_{X_{1}},$${\bar{Q}}_{X_{2}}$, and ${\bar{Q}}_{0}$, respectively. Note that $F_{f_{1},f_{2}}^{P}⊢{\bar{Q}}_{F}$ is equivalent to ${\bar{E}}_{U}\wedge {\bar{E}}_{V}\wedge {\bar{E}}_{X_{1}}\wedge {\bar{E}}_{X_{2}}\wedge {\bar{E}}_{0}$. Therefore, it holds that

$$\begin{array}{cc} p(\bar{\tau }) & =\mathrm{Pr}[P\overset{\$}{\leftarrow }\mathrm{Perm}\left(n\right):F_{f_{1},f_{2}}^{P}⊢{\bar{Q}}_{F}\;|\;P⊢{\bar{Q}}_{P}]\\ \; & =\mathrm{Pr}[P\overset{\$}{\leftarrow }\mathrm{Perm}\left(n\right):{\bar{E}}_{U}\wedge {\bar{E}}_{V}\wedge {\bar{E}}_{X_{1}}\wedge {\bar{E}}_{X_{2}}\wedge {\bar{E}}_{0}\;|\;P⊢{\bar{Q}}_{P}]\\ \; & =p^{\prime }\left(\bar{\tau }\right)\cdot p^{\prime \prime }\left(\bar{\tau }\right), \end{array}$$

where

$$p^{\prime }\left(\bar{\tau }\right)=\mathrm{Pr}\left[P\overset{\$}{\leftarrow }\mathrm{Perm}\left(n\right):{\bar{E}}_{U}\wedge {\bar{E}}_{V}\;|\;P⊢{\bar{Q}}_{P}\right],$$

and

$$p^{\prime \prime }\left(\bar{\tau }\right)=\mathrm{Pr}\left[P\overset{\$}{\leftarrow }\mathrm{Perm}\left(n\right):{\bar{E}}_{X_{1}}\wedge {\bar{E}}_{X_{2}}\wedge {\bar{E}}_{0}\;|\;{\bar{E}}_{U}\wedge {\bar{E}}_{V}\wedge \left(P⊢{\bar{Q}}_{P}\right)\right].$$

The next goal is to lower bound $p^{\prime }\left(\bar{\tau }\right)$ and $p^{\prime \prime }\left(\bar{\tau }\right)$.

$\mathrm{Lower}\;\mathrm{Bounding}\;p^{\prime }\left(\bar{\tau }\right)$. Conditioned on $P⊢{\bar{Q}}_{P}$, *P* is fixed on exactly *p* input-output pairs from *U* to *V*. For each $\left(t,x,y\right)\in {\bar{Q}}_{U}$, there exists a unique $\left(u,v\right)\in {\bar{Q}}_{P}$ satisfying $x⊕f_{1}\left(t\right)=u$. Hence, $P(x⊕f_{1}\left(t\right))=P\left(u\right)=v$. Then we define two sets:

$$\begin{array}{cc} \; & {\bar{U}}_{1}=\left\{P\left(x⊕f_{1}\left(t\right)\right)⊕f_{1}\left(t\right)⊕f_{2}\left(t\right)⊕y:\left(t,x,y\right)\in {\bar{Q}}_{U}\right\},\\ \; & {\bar{V}}_{1}=\left\{x⊕f_{2}\left(t\right):\left(t,x,y\right)\in {\bar{Q}}_{U}\right\}. \end{array}$$

All values in ${\bar{U}}_{1}$ (resp. ${\bar{V}}_{1}$) are distinct since τ does not satisfy (C-11) (resp. (C-6)). Moreover, since $\bar{\tau }\notin \Gamma_{2}^{\prime }$ and $\bar{\tau }\notin \Gamma_{1}^{\prime }$, one has ${\bar{U}}_{1}\cap U=\emptyset$ and ${\bar{V}}_{1}\cap V=\emptyset$ respectively.

For each $\left(t,x,y\right)\in {\bar{Q}}_{V}$, there exists a unique $\left(u,v\right)\in {\bar{Q}}_{P}$ satisfying $x⊕f_{2}\left(t\right)=v$. In this case, $P^{-1}\left(x⊕f_{2}\left(t\right)\right)=u$. Then we can define two sets:

$$\begin{array}{cc} \; & {\bar{U}}_{2}=\left\{x⊕f_{1}\left(t\right):\left(t,x,y\right)\in {\bar{Q}}_{V}\right\},\\ \; & {\bar{V}}_{2}=\left\{P^{-1}\left(x⊕f_{2}\left(t\right)\right)⊕f_{1}\left(t\right)⊕f_{2}\left(t\right)⊕y:\left(t,x,y\right)\in {\bar{Q}}_{V}\right\}. \end{array}$$

All elements in ${\bar{U}}_{2}$ (resp. ${\bar{V}}_{2}$) are distinct since $\bar{\tau }$ does not satisfy (C-7) (resp. (C-12)). Due to the fact $\bar{\tau }\notin \Gamma_{1}^{\prime }$ and $\bar{\tau }\notin \Gamma_{3}^{\prime }$, one has ${\bar{U}}_{2}\cap U=\emptyset$ and ${\bar{V}}_{2}\cap V=\emptyset$, respectively. Moreover, ${\bar{U}}_{2}\cap {\bar{U}}_{1}=\emptyset$ (resp. ${\bar{V}}_{2}\cap {\bar{V}}_{1}=\emptyset$) since $\bar{\tau }\notin \Gamma_{4}^{\prime }$ (resp. $\bar{\tau }\notin \Gamma_{5}^{\prime }$). Besides, it holds that $|{\bar{U}}_{1}\left|=\right|{\bar{V}}_{1}\left|=\right|{\bar{Q}}_{U}|={\bar{\alpha }}_{1}$ and $|{\bar{U}}_{2}\left|=\right|{\bar{V}}_{2}\left|=\right|{\bar{Q}}_{V}|={\bar{\alpha }}_{2}$. Therefore, one can obtain that

(32) $$\begin{array}{c} p^{\prime }\left(\bar{\tau }\right)=\mathrm{Pr}\left[P\overset{\$}{\leftarrow }\mathrm{Perm}\left(n\right):E_{U}\wedge E_{V}\;|\;P⊢{\bar{Q}}_{P}\right]=\frac{1}{{\left(2^{n}-p\right)}_{{\bar{\alpha }}_{1}+{\bar{\alpha }}_{2}}}. \end{array}$$

Now, we can define two disjoint collections $U\overset{\mathrm{def}}{=}\left(U,{\bar{U}}_{1},{\bar{U}}_{2}\right)$ and $V\overset{\mathrm{def}}{=}\left(V,{\bar{V}}_{1},{\bar{V}}_{2}\right)$. In this case, *P* is fixed on exactly $p+{\bar{\alpha }}_{1}+{\bar{\alpha }}_{2}$ input-output pairs from $U\cup {\bar{U}}_{1}\cup {\bar{U}}_{2}$ to $V\cup {\bar{V}}_{1}\cup {\bar{V}}_{2}$.

$\mathrm{Lower}\;\mathrm{Bounding}\;p^{\prime \prime }\left(\bar{\tau }\right)$. When conditioned on ${\bar{E}}_{U}\wedge {\bar{E}}_{V}\wedge \left(P⊢{\bar{Q}}_{P}\right)$, we next lower bound the number of all possible “new” and distinct input-output pairs of *P* such that the event ${\bar{E}}_{X_{1}}\wedge {\bar{E}}_{X_{2}}\wedge {\bar{E}}_{0}$ happens. First, one can define some multi-sets associated to ${\bar{Q}}_{X_{1}}$ and ${\bar{Q}}_{X_{2}}$ as follows:

$$\begin{array}{cc} \; & U_{3}=\left\{x⊕f_{1}\left(t\right):\left(t,x,y\right)\in {\bar{Q}}_{X_{1}}\right\},\;\;U_{5}=\left\{x⊕f_{1}\left(t\right):\left(t,x,y\right)\in {\bar{Q}}_{X_{2}}\right\},\\ \; & V_{4}=\left\{x⊕f_{2}\left(t\right):\left(t,x,y\right)\in {\bar{Q}}_{X_{1}}\right\},\;\;V_{6}=\left\{x⊕f_{2}\left(t\right):\left(t,x,y\right)\in {\bar{Q}}_{X_{2}}\right\}. \end{array}$$

Let $\alpha_{3}=\left|U_{3}\right|$, $\alpha_{4}=\left|V_{4}\right|$, $\alpha_{5}=\left|U_{5}\right|$, and $\alpha_{6}=\left|V_{6}\right|$. For convenience, we rewrite these sets as:

$$\begin{array}{cc} \; & U_{3}=\left\{u_{3,1},\ldots ,u_{3,\alpha_{3}}\right\},\;\;U_{5}=\left\{u_{5,1},\ldots ,u_{5,\alpha_{5}}\right\},\\ \; & V_{4}=\left\{v_{4,1},\ldots ,v_{4,\alpha_{4}}\right\},\;\;V_{6}=\left\{v_{6,1},\ldots ,v_{6,\alpha_{6}}\right\}. \end{array}$$

Let $V_{3}=P\left(U_{3}\right)$, $U_{4}=P^{-1}\left(V_{4}\right)$, $V_{5}=P\left(U_{5}\right)$, and $U_{6}=P^{-1}\left(V_{6}\right)$. These sets can be written more clearly as:

$$\begin{array}{cc} \; & V_{3}=\left\{P\left(x⊕f_{1}\left(t\right)\right):\left(t,x,y\right)\in {\bar{Q}}_{X_{1}}\right\},\;\;V_{5}=\left\{P\left(x⊕f_{1}\left(t\right)\right):\left(t,x,y\right)\in {\bar{Q}}_{X_{2}}\right\},\\ \; & U_{4}=\left\{P^{-1}\left(x⊕f_{2}\left(t\right)\right):\left(t,x,y\right)\in {\bar{Q}}_{X_{1}}\right\},\;\;U_{6}=\left\{P^{-1}\left(x⊕f_{2}\left(t\right)\right):\left(t,x,y\right)\in {\bar{Q}}_{X_{2}}\right\}. \end{array}$$

Recall that ${\bar{D}}_{1}=\left\{x⊕f_{1}\left(t\right):\left(t,x,y\right)\in {\bar{Q}}_{F}\right\}$ and ${\bar{D}}_{2}=\left\{x⊕f_{2}\left(t\right):\left(t,x,y\right)\in {\bar{Q}}_{F}\right\}$. Then, we get

α3≤∑x∈{0,1}n:δD¯1(x)>11≤∑x∈{0,1}n:δD¯1(x)>1δD¯1(x)2=β¯12≤q2,α4≤∑i=1α3δD¯1(u3,i)≤∑x∈{0,1}n:δD¯1(x)>1δD¯1(x)=β¯1≤q.

Similarly, it also holds that $\alpha_{6}\leq \frac{\sqrt{q}}{2}$ and $\alpha_{5}\leq \sqrt{q}$. Since $\bar{\tau }\notin \Gamma_{10}^{\prime }$, there exists no repeated items in $V_{4}$ and $U_{5}$. Hence, one can conclude that $\alpha_{4}=\left|{\bar{Q}}_{X_{1}}\right|$ and $\alpha_{5}=\left|{\bar{Q}}_{X_{2}}\right|$. Now we define two multi-sets associated to ${\bar{Q}}_{0}$ as

$$U_{7}=\left\{x⊕f_{1}\left(t\right):\left(t,x,y\right)\in {\bar{Q}}_{0}\right\},\;\;V_{8}=\left\{x⊕f_{2}\left(t\right):\left(t,x,y\right)\in {\bar{Q}}_{0}\right\}.$$

By the definition of ${\bar{Q}}_{0}$, there exists no repeated items in $U_{7}$ and $V_{8}$. Based on these two sets, one can define two corresponding sets as:

$$\begin{array}{cc} \; & V_{7}=P\left(U_{7}\right)=\left\{P\left(x⊕f_{1}\left(t\right)\right):\left(t,x,y\right)\in {\bar{Q}}_{0}\right\},\\ \; & U_{8}=P^{-1}\left(V_{8}\right)=\left\{P^{-1}\left(x⊕f_{2}\left(t\right)\right):\left(t,x,y\right)\in {\bar{Q}}_{0}\right\}. \end{array}$$

Set $U^{+}=\left(U_{3},U_{5},U_{7}\right)$ and $V^{+}=\left(V_{4},V_{6},V_{8}\right)$ as two set collections. Then we can conclude the following proposition.

**Proposition** **3.** *With notations as above, one has*

*(i)*  *All sets in $U^{+}$ (resp. $V^{+}$) are disjoint, i.e., $U_{3}\cap U_{5}=\emptyset$, $U_{3}\cap U_{7}=\emptyset$, and $U_{5}\cap U_{7}=\emptyset$ (resp. $V_{4}\cap V_{6}=\emptyset$, $V_{4}\cap V_{8}=\emptyset$, and $V_{6}\cap V_{8}=\emptyset$).*
*(ii)*  *$U^{+}$ is inner disjoint with U, and $V^{+}$ is inner disjoint with V.*

**Proof.**  We first prove (i). From the fact $\bar{\tau }\notin \Gamma_{10}^{\prime }$, we have $U_{3}\cap U_{5}=\emptyset$. By the definition of ${\bar{Q}}_{X_{1}}$ and ${\bar{Q}}_{0}$, one can conclude that $U_{3}\cap U_{7}=\emptyset$. $U_{5}\cap U_{7}=\emptyset$ holds due to the fact $\bar{\tau }\notin \Gamma_{10}^{\prime }$, and the disjoint property of ${\bar{Q}}_{X_{1}}$ and ${\bar{Q}}_{0}$. We can conclude that $V_{4}\cap V_{6}=\emptyset$, $V_{4}\cap V_{8}=\emptyset$, and $V_{6}\cap V_{8}=\emptyset$ in a similar way.

Next we prove (ii) by enumerating all possible cases. For $U_{3}$, the definition of ${\bar{Q}}_{X_{1}}$ means that $U_{3}\cap U=\emptyset$; $U_{3}\cap {\bar{U}}_{1}=\emptyset$ comes from the fact $\bar{\tau }\notin \Gamma_{4}^{\prime }$; $U_{3}\cap {\bar{U}}_{2}=\emptyset$ holds due to the disjoint property between ${\bar{Q}}_{X_{1}}$ and ${\bar{Q}}_{V}$, and the fact $\bar{\tau }\notin \Gamma_{7}^{\prime }$. For $U_{5}$, $U_{5}\cap U=\emptyset$ comes from the fact $\bar{\tau }\notin \Gamma_{6}^{\prime }$, and the definition of ${\bar{Q}}_{X_{2}}$; $U_{5}\cap {\bar{U}}_{1}=\emptyset$ comes from the fact $\bar{\tau }\notin \Gamma_{4}^{\prime }$; By the disjoint property between ${\bar{Q}}_{X_{2}}$ and ${\bar{Q}}_{V}$, and the fact $\bar{\tau }\notin \Gamma_{7}^{\prime }$, we have $U_{5}\cap {\bar{U}}_{2}=\emptyset$. For $U_{7}$, the definition of ${\bar{Q}}_{0}$ means $U_{7}\cap U=\emptyset$; $U_{7}\cap {\bar{U}}_{1}=\emptyset$ comes from the fact that $\bar{\tau }\notin \Gamma_{4}^{\prime }$; By the disjoint property between ${\bar{Q}}_{0}$ and ${\bar{Q}}_{V}$, and the fact $\bar{\tau }\notin \Gamma_{7}^{\prime }$, we has $U_{7}\cap {\bar{U}}_{2}=\emptyset$.

For $V_{4}$, $V_{4}\cap V=\emptyset$ comes from the fact $\bar{\tau }\notin \Gamma_{7}^{\prime }$, and the definition of ${\bar{Q}}_{X_{1}}$; $V_{4}\cap {\bar{V}}_{1}=\emptyset$ can be derived from the disjoint property between ${\bar{Q}}_{X_{1}}$ and ${\bar{Q}}_{U}$, and the fact $\bar{\tau }\notin \Gamma_{6}^{\prime }$; The fact $\bar{\tau }\notin \Gamma_{5}^{\prime }$ means $V_{4}\cap {\bar{V}}_{2}=\emptyset$. For $V_{6}$, $V_{6}\cap V=\emptyset$ holds from definition of ${\bar{Q}}_{X_{2}}$; $V_{6}\cap V_{1}=\emptyset$ comes from the definition of ${\bar{Q}}_{X_{2}}$, and the fact $\bar{\tau }\notin \Gamma_{6}^{\prime }$; The fact $\bar{\tau }\notin \Gamma_{5}^{\prime }$ means $V_{6}\cap {\bar{V}}_{2}=\emptyset$. For $V_{8}$, $V_{8}\cap V=\emptyset$ comes from definition of ${\bar{Q}}_{0}$; $V_{8}\cap {\bar{V}}_{1}=\emptyset$ holds due to the disjoint property between ${\bar{Q}}_{0}$ and ${\bar{Q}}_{U}$, and the fact $\bar{\tau }\notin \Gamma_{6}^{\prime }$; Finally the fact $\bar{\tau }\notin \Gamma_{5}^{\prime }$ means $V_{8}\cap {\bar{V}}_{2}=\emptyset$.   □

Now we define two disjoint union sets $U^{++}=U\cup {\bar{U}}_{1}\cup {\bar{U}}_{2}\cup U_{3}\cup U_{5}\cup U_{7}$ (which equals to $\hat{U}$ in (C-17)), and $V^{++}=V\cup {\bar{V}}_{1}\cup {\bar{V}}_{2}\cup V_{4}\cup V_{6}\cup V_{8}$ (which equals to $\hat{V}$ in (C-17)).

Let ${\bar{q}}^{\prime }=|{\bar{Q}}_{0}\left|=q-(\right|{\bar{Q}}_{U}\left|+\right|{\bar{Q}}_{V}\left|+\right|{\bar{Q}}_{X_{1}}\left|+\right|{\bar{Q}}_{X_{2}}\left|\right)=q-\left({\bar{\alpha }}_{1}+{\bar{\alpha }}_{2}+\alpha_{4}+\alpha_{5}\right)$ (actually, ${\bar{q}}^{\prime }=|U_{7}\left|=\right|V_{8}|$) and $M=\lfloor \frac{{\bar{q}}^{\prime }}{2^{n/3}}\rfloor$. Then it holds that ${\bar{q}}^{\prime }-2M\geq {\bar{q}}^{\prime }/2$ if n≥6. Next we try to sample “new” values for $V_{7}$ and $U_{8}$ by allowing that there exist many construction queries $\left(t,x,y\right),\left(t^{\prime },x^{\prime },y^{\prime }\right)\in {\bar{Q}}_{0}$ such that $P\left(x⊕f_{1}\left(t\right)\right)⊕f_{1}\left(t\right)⊕f_{2}\left(t\right)⊕y=x^{\prime }⊕f_{1}\left(t^{\prime }\right)$ or $P^{-1}\left(x⊕f_{2}\left(t\right)\right)⊕f_{1}\left(t\right)⊕f_{2}\left(t\right)⊕y=x^{\prime }⊕f_{2}\left(t^{\prime }\right)$ holds. In the first case, we can obtain three maps like

$$\left\{ \begin{array}{c} x⊕f_{1}\left(t\right)\overset{P}{⟼}x^{\prime }⊕f_{1}\left(t^{\prime }\right)⊕f_{1}\left(t\right)⊕f_{2}\left(t\right)⊕y,\\ x^{\prime }⊕f_{1}\left(t^{\prime }\right)\overset{P}{⟼}x⊕f_{2}\left(t\right),\\ x⊕f_{2}\left(t\right)⊕f_{1}\left(t^{\prime }\right)⊕f_{2}\left(t^{\prime }\right)⊕y^{\prime }\overset{P}{⟼}x^{\prime }⊕f_{2}\left(t^{\prime }\right). \end{array}\right.$$

In the second case, we have

$$\left\{ \begin{array}{c} x^{\prime }⊕f_{1}\left(t^{\prime }\right)\overset{P}{⟼}x⊕f_{1}\left(t\right)⊕f_{1}\left(t^{\prime }\right)⊕f_{2}\left(t^{\prime }\right)⊕y^{\prime },\\ x⊕f_{1}\left(t\right)\overset{P}{⟼}x^{\prime }⊕f_{2}\left(t^{\prime }\right),\\ x^{\prime }⊕f_{2}\left(t^{\prime }\right)⊕f_{1}\left(t\right)⊕f_{2}\left(t\right)⊕y\overset{P}{⟼}x⊕f_{2}\left(t\right). \end{array}\right.$$

If $x⊕f_{2}\left(t\right)⊕f_{1}\left(t^{\prime }\right)⊕f_{2}\left(t^{\prime }\right)⊕y^{\prime }\notin U^{++}$ and $x^{\prime }⊕f_{1}\left(t^{\prime }\right)⊕f_{1}\left(t\right)⊕f_{2}\left(t\right)⊕y\notin V^{++}$, or $x^{\prime }⊕f_{2}\left(t^{\prime }\right)⊕f_{1}\left(t\right)⊕f_{2}\left(t\right)⊕y\notin U^{++}$ and $x⊕f_{1}\left(t\right)⊕f_{1}\left(t^{\prime }\right)⊕f_{2}\left(t^{\prime }\right)⊕y^{\prime }\notin V^{++}$, then the above permutation maps are compatible with ${\bar{Q}}_{F}$ and ${\bar{Q}}_{P}$. Intuitively, when we consider the above “collision” maps, there would be as many permutations chosen to be compatible with ${\bar{Q}}_{F}$ and ${\bar{Q}}_{P}$ as possible so that our construction can achieve BBB security.

Conditioned on ${\bar{E}}_{U}\wedge {\bar{E}}_{V}\wedge \left(P⊢{\bar{Q}}_{P}\right)$, we next describe all possible permutations satisfying $E_{X_{1}}\wedge E_{X_{2}}\wedge E_{0}$, and finally compute and lower bound $p^{\prime \prime }\left(\bar{\tau }\right)$.

For each α∈[M], we define the following set

$$S=\{\left(\left(\sigma_{1},\xi_{1}\right),\left(\sigma_{1}^{\prime },\xi_{1}^{\prime }\right)\right),\ldots ,\left(\left(\sigma_{\alpha },\xi_{\alpha }\right),\left(\sigma_{\alpha }^{\prime },\xi_{\alpha }^{\prime }\right)\right)\},$$

where for each 1≤k≤α, one has $\sigma_{k}=x_{k}⊕f_{1}\left(t_{k}\right)$ (resp. $\xi_{k}=x_{k}⊕f_{2}\left(t_{k}\right)$) for some query $\left(t_{k},x_{k},y_{k}\right)\in {\bar{Q}}_{0}$ and $\sigma_{k}^{\prime }=x_{k}^{\prime }⊕f_{1}\left(t_{k}^{\prime }\right)$ (resp. $\xi_{k}^{\prime }=x_{k}^{\prime }⊕f_{2}\left(t_{k}^{\prime }\right)$) for another query $\left(t_{k}^{\prime },x_{k}^{\prime },y_{k}^{\prime }\right)\in {\bar{Q}}_{0}$.

**Definition** **2.** *We say $S=\{\left(\left(\sigma_{1},\xi_{1}\right),\left(\sigma_{1}^{\prime },\xi_{1}^{\prime }\right)\right),\ldots ,\left(\left(\sigma_{\alpha },\xi_{\alpha }\right),\left(\sigma_{\alpha }^{\prime },\xi_{\alpha }^{\prime }\right)\right)\}$ a “good” set if the following four conditions are all satisfied*

*(1)*  *$x_{k}⊕f_{2}\left(t_{k}\right)⊕f_{1}\left(t_{k}^{\prime }\right)⊕f_{2}\left(t_{k}^{\prime }\right)⊕y_{k}^{\prime }\notin U^{++}$,*
*(2)*  *$x_{k}^{\prime }⊕f_{1}\left(t_{k}^{\prime }\right)⊕f_{1}\left(t_{k}\right)⊕f_{2}\left(t_{k}\right)⊕y_{k}\notin V^{++}$,*
*(3)*  *$x_{k}⊕f_{2}\left(t_{k}\right)⊕f_{1}\left(t_{k}^{\prime }\right)⊕f_{2}\left(t_{k}^{\prime }\right)⊕y_{k}^{\prime }\neq x_{k^{\prime }}⊕f_{2}\left(t_{k^{\prime }}\right)⊕f_{1}\left(t_{k^{\prime }}^{\prime }\right)⊕f_{2}\left(t_{k^{\prime }}^{\prime }\right)⊕y_{k^{\prime }}^{\prime }$, for any $k^{\prime }<k$,*
*(4)*  *$x_{k}^{\prime }⊕f_{1}\left(t_{k}^{\prime }\right)⊕f_{1}\left(t_{k}\right)⊕f_{2}\left(t_{k}\right)⊕y_{k}\neq x_{k^{\prime }}^{\prime }⊕f_{1}\left(t_{k^{\prime }}^{\prime }\right)⊕f_{1}\left(t_{k^{\prime }}\right)⊕f_{2}\left(t_{k^{\prime }}\right)⊕y_{k^{\prime }}$, for any $k^{\prime }<k$.*

The next lemma shows that for each α∈[M], the number of all possible “good” sets derived from ${\bar{Q}}_{0}$ is close to ${\left({\bar{q}}^{\prime }\right)}_{2\alpha }/\alpha !$.

**Lemma** **7.** *Assume that q≥64 and n≥6. Let α be an integer with $0\leq \alpha \leq M=\lfloor \frac{{\bar{q}}^{\prime }}{2^{n/3}}\rfloor$. Let $N_{S}\left(\alpha \right)$ be the number of all “good” sets derived from ${\bar{Q}}_{0}$. Then we have*

$$N_{S}\left(\alpha \right)\geq \frac{{\left({\bar{q}}^{\prime }\right)}_{2\alpha }}{\alpha !}\left(1-\epsilon_{0}\right),$$

*where $\epsilon_{0}=\frac{6q}{2^{2n/3}}+\frac{16\sqrt{q}}{2^{n/3}}$.*

**Proof.**  We count all possible pairs in a “good” set step by step as follows. First, we decide all possible pairs for $\left(\left(\sigma_{1},\xi_{1}\right),\left(\sigma_{1}^{\prime },\xi_{1}^{\prime }\right)\right)$. There are ${\bar{q}}^{\prime }\left({\bar{q}}^{\prime }-1\right)$ possible pairs to be chosen for $\left(\left(\sigma_{1},\xi_{1}\right),\left(\sigma_{1}^{\prime },\xi_{1}^{\prime }\right)\right)$. Since $\tau \notin \Gamma_{17}^{\prime }$, there are at most $2q^{3/2}$ pairs not satisfying the first two conditions in Definition 2. Then we can choose at least ${\bar{q}}^{\prime }\left({\bar{q}}^{\prime }-1\right)-2q^{3/2}$ possible pairs for $\left(\left(\sigma_{1},\xi_{1}\right),\left(\sigma_{1}^{\prime },\xi_{1}^{\prime }\right)\right)$.

After choosing $\left(\sigma_{1},\xi_{1}\right),\left(\sigma_{1}^{\prime },\xi_{1}^{\prime }\right)$, we decide all possible $\left(\left(\sigma_{2},\xi_{2}\right),\left(\sigma_{2}^{\prime },\xi_{2}^{\prime }\right)\right)$ in the following way. We first choose $\left(\sigma_{2},\xi_{2}\right)$ from the remaining ${\bar{q}}^{\prime }-2$ possible pairs, and then choose the corresponding pair $\left(\sigma_{2}^{\prime },\xi_{2}^{\prime }\right)$ outside of $\left(\sigma_{1},\xi_{1}\right)$, $\left(\sigma_{1}^{\prime },\xi_{1}^{\prime }\right)$, and $\left(\sigma_{2}^{\prime },\xi_{2}^{\prime }\right)$ to satisfy all four conditions in Definition 2. To satisfy the last two conditions 3) and 4) in Definition 2, $\sigma_{2}^{\prime }$ and $\xi_{2}^{\prime }$ should chosen such that

$$\left\{ \begin{array}{c} \xi_{2}\neq \xi_{1}⊕f_{1}\left(t_{1}^{\prime }\right)⊕f_{2}\left(t_{1}^{\prime }\right)⊕y_{1}^{\prime }⊕f_{1}\left(t_{2}^{\prime }\right)⊕f_{2}\left(t_{2}^{\prime }\right)⊕y_{2}^{\prime },\\ \sigma_{2}^{\prime }\neq \sigma_{1}^{\prime }⊕f_{1}\left(t_{1}\right)⊕f_{2}\left(t_{1}\right)⊕y_{1}⊕f_{1}\left(t_{2}\right)⊕f_{2}\left(t_{2}\right)⊕y_{2}. \end{array}\right.$$

In this case, from the definition of ${\bar{Q}}_{0}$ and the fact $\bar{\tau }\notin \Gamma_{16}^{\prime }$, it excludes at most 3 possibilities to be chosen for $\left(\sigma_{2}^{\prime },\xi_{2}^{\prime }\right)$. Then there are at least $\left({\bar{q}}^{\prime }-2\right)\left({\bar{q}}^{\prime }-6\right)$ possibilities to be chosen for $\left(\left(\sigma_{2},\xi_{2}\right),\left(\sigma_{2}^{\prime },\xi_{2}^{\prime }\right)\right)$, when we only consider the last two conditions in Definition 2. Finally, from the fact $\tau \notin \Gamma_{17}^{\prime }$, there are at most $2q^{3/2}$ pairs to be removed for all possibilities $\left(\left(\sigma_{2},\xi_{2}\right),\left(\sigma_{2}^{\prime },\xi_{2}^{\prime }\right)\right)$ if we want them to satisfy the first two conditions 1) and 2) in Definition 2. Overall, there are at least $\left({\bar{q}}^{\prime }-2\right)\left({\bar{q}}^{\prime }-6\right)-2q^{3/2}$ possible pairs to be chosen for $\left(\left(\sigma_{2},\xi_{2}\right),\left(\sigma_{2}^{\prime },\xi_{2}^{\prime }\right)\right)$.

After choosing k−1 pairs $\left(\left(\sigma_{1},\xi_{1}\right),\left(\sigma_{1}^{\prime },\xi_{1}^{\prime }\right)\right)$, …, $\left(\left(\sigma_{k-1},\xi_{k-1}\right),\left(\sigma_{k-1}^{\prime },\xi_{k-1}^{\prime }\right)\right)$, there are at least $\left({\bar{q}}^{\prime }-2k\right)\left({\bar{q}}^{\prime }-5k-1\right)-2q^{3/2}$ possible pairs to be chosen for $\left(\left(\sigma_{k},\xi_{k}\right),\left(\sigma_{k}^{\prime },\xi_{k}^{\prime }\right)\right)$ by repeating the above step.

When we finish the choice of all possible cases for $(\left(\left(\sigma_{1},\xi_{1}\right),\left(\sigma_{1}^{\prime },\xi_{1}^{\prime }\right)\right),\ldots ,(\left(\sigma_{\alpha },\xi_{\alpha }\right)$, $\left(\sigma_{\alpha }^{\prime },\xi_{\alpha }^{\prime }))\right)$ satisfying all four conditions in Definition 2, one can conclude that

(33) $$N_{S}\left(\alpha \right)\geq \frac{1}{\alpha !}\prod_{k=0}^{\alpha -1}\left(\left({\bar{q}}^{\prime }-2k\right)\left({\bar{q}}^{\prime }-5k-1\right)-2q^{3/2}\right),$$

where the term α! appears because the set *S* is unordered.

Furthermore, $N_{S}\left(\alpha \right)$ can be lower bounded as follows

$$\begin{array}{cc} N_{S}\left(\alpha \right) & \geq \frac{1}{\alpha !}\prod_{k=0}^{\alpha -1}\left(\left({\bar{q}}^{\prime }-2k\right)\left({\bar{q}}^{\prime }-5k-1\right)-2q^{3/2}\right)\\ \; & \geq \frac{{\left({\bar{q}}^{\prime }\right)}_{2\alpha }}{\alpha !}\prod_{k=0}^{\alpha -1}\frac{\left({\bar{q}}^{\prime }-2k\right)\left({\bar{q}}^{\prime }-5k-1\right)-2q^{3/2}}{\left({\bar{q}}^{\prime }-2k\right)\left({\bar{q}}^{\prime }-2k-1\right)}\\ \; & \geq \frac{{\left({\bar{q}}^{\prime }\right)}_{2\alpha }}{\alpha !}\prod_{k=0}^{\alpha -1}\left(1-\frac{3k{\bar{q}}^{\prime }-6k^{2}+2q^{3/2}}{\left({\bar{q}}^{\prime }-2k\right)\left({\bar{q}}^{\prime }-2k-1\right)}\right)\\ \; & \overset{\left(i\right)}{\geq }\frac{{\left({\bar{q}}^{\prime }\right)}_{2\alpha }}{\alpha !}\prod_{k=0}^{\alpha -1}\left(1-\frac{3k{\bar{q}}^{\prime }+2q^{3/2}}{{\left({\bar{q}}^{\prime }-2M\right)}^{2}}\right)\\ \; & \geq \frac{{\left({\bar{q}}^{\prime }\right)}_{2\alpha }}{\alpha !}\left(1-\frac{3{\bar{q}}^{\prime }M^{2}/2+2q^{3/2}M}{{\left({\bar{q}}^{\prime }-2M\right)}^{2}}\right)\\ \; & \overset{\left(ii\right)}{\geq }\frac{{\left({\bar{q}}^{\prime }\right)}_{2\alpha }}{\alpha !}\left(1-\frac{6{\bar{q}}^{\prime }M^{2}+8q^{3/2}M}{{\bar{q}}^{\prime 2}}\right)\\ \; & \overset{\left(iii\right)}{\geq }\frac{{\left({\bar{q}}^{\prime }\right)}_{2\alpha }}{\alpha !}\left(1-\frac{6{\bar{q}}^{\prime }}{2^{2n/3}}-\frac{8q^{3/2}}{q^{\prime }2^{n/3}}\right)\\ \; & \overset{\left(iv\right)}{\geq }\frac{{\left({\bar{q}}^{\prime }\right)}_{2\alpha }}{\alpha !}\left(1-\frac{6q}{2^{2n/3}}-\frac{16\sqrt{q}}{2^{n/3}}\right), \end{array}$$

where (i) follows as ${\bar{q}}^{\prime }-2k,q^{\prime }-2k-1\geq {\bar{q}}^{\prime }-2\alpha \geq {\bar{q}}^{\prime }-2M$, (ii) follows as ${\bar{q}}^{\prime }-2M>{\bar{q}}^{\prime }/2$, (iii) follows as $M\leq \frac{{\bar{q}}^{\prime }}{2^{n/3}}$, and (iv) follows as $q/2\leq q-4\sqrt{q}\leq {\bar{q}}^{\prime }$ if q>64.   □

For a fixed α with 0≤α≤M and a corresponding “good” set

$$S=\{\left(\left(\sigma_{1},\xi_{1}\right),\left(\sigma_{1}^{\prime },\xi_{1}^{\prime }\right)\right),\ldots ,\left(\left(\sigma_{\alpha },\xi_{\alpha }\right),\left(\sigma_{\alpha }^{\prime },\xi_{\alpha }^{\prime }\right)\right)\},$$

the following assignment (34) for *P* is well-defined by the definition of *S*:

(34) $$\forall k\in \left[\alpha \right]\;\;\;\left\{ \begin{array}{c} \sigma_{k}\overset{P}{⟼}\sigma_{k}^{\prime }⊕f_{1}\left(t_{k}\right)⊕f_{2}\left(t_{k}\right)⊕y_{k},\\ \sigma_{k}^{\prime }\overset{P}{⟼}\xi_{k},\\ \xi_{k}⊕f_{1}\left(t_{k}^{\prime }\right)⊕f_{2}\left(t_{k}^{\prime }\right)⊕y_{k}^{\prime }\overset{P}{⟼}\xi_{k}^{\prime }. \end{array}\right.$$

Furthermore, based on the “good” set *S*, we define two subsets of $U_{7}$ and $V_{8}$ as

$$\begin{array}{cc} \; & U_{7,1}\overset{def}{=}\left\{\sigma_{1}=x_{1}⊕f_{1}\left(t_{1}\right),\sigma_{1}^{\prime }=x_{1}^{\prime }⊕f_{1}\left(t_{1}^{\prime }\right),\ldots ,\sigma_{\alpha }=x_{\alpha }⊕f_{1}\left(t_{\alpha }\right),\sigma_{\alpha }^{\prime }=x_{\alpha }^{\prime }⊕f_{1}\left(t_{\alpha }^{\prime }\right)\right\},\\ \; & V_{8,1}\overset{def}{=}\left\{\xi_{1}=x_{1}⊕f_{2}\left(t_{1}\right),\xi_{1}^{\prime }=x_{1}^{\prime }⊕f_{2}\left(t_{1}^{\prime }\right),\ldots ,\xi_{\alpha }=x_{\alpha }⊕f_{2}\left(t_{\alpha }\right),\xi_{\alpha }^{\prime }=x_{\alpha }^{\prime }⊕f_{2}\left(t_{\alpha }^{\prime }\right)\right\}. \end{array}$$

Besides, we can also denote two additional sets as

$$\begin{array}{cc} \; & U_{7,1}^{\prime }\overset{def}{=}\left\{\xi_{1}⊕f_{1}\left(t_{1}^{\prime }\right)⊕f_{2}\left(t_{1}^{\prime }\right)⊕y_{1}^{\prime },\ldots ,\xi_{\alpha }⊕f_{1}\left(t_{\alpha }^{\prime }\right)⊕f_{2}\left(t_{\alpha }^{\prime }\right)⊕y_{\alpha }^{\prime }\right\},\\ \; & V_{8,1}^{\prime }\overset{def}{=}\left\{\sigma_{1}^{\prime }⊕f_{1}\left(t_{1}\right)⊕f_{2}\left(t_{1}\right)⊕y_{1},\ldots ,\sigma_{\alpha }^{\prime }⊕f_{1}\left(t_{\alpha }\right)⊕f_{2}\left(t_{\alpha }\right)⊕y_{\alpha }\right\}, \end{array}$$

where $U_{7,1}^{\prime }\cap U^{++}=\emptyset$ (resp. $V_{8,1}^{\prime }\cap V^{++}=\emptyset$) and all items in $U_{7,1}^{\prime }$ (resp. $V_{8,1}^{\prime }$) are distinct. After the assignment (34) for *P*, *P* is fixed on 3α input-ouput pairs from $U_{7,1}\cup U_{7,1}^{\prime }$ to $V_{8,1}\cup V_{8,1}^{\prime }$. In addition, we can define the corresponding co-subset of $U_{7,1}$ and $V_{8,1}$ as $U_{7,2}\overset{def}{=}U_{7}\backslash U_{7,1}$ and $V_{8,2}\overset{def}{=}V_{8}\backslash V_{8,2}$, respectively.

Until now, the random permutation *P* is fixed on *p* input-output pairs from *U* to *V*, ${\bar{\alpha }}_{1}$ input-output pairs from ${\bar{U}}_{1}$ to ${\bar{V}}_{1}$, ${\bar{\alpha }}_{2}$ input-output pairs from ${\bar{U}}_{2}$ to ${\bar{V}}_{2}$, and 3α input-output pairs from $U_{7,1}\cup U_{7,1}^{\prime }$ to $V_{8,1}\cup V_{8,1}^{\prime }$. Based on these facts, the next work is to choose all other possible compatible items for $V_{3}=P\left(U_{3}\right)$, $U_{4}=P^{-1}\left(V_{4}\right)$, $V_{5}=P\left(U_{5}\right)$, $U_{6}=P^{-1}\left(V_{6}\right)$, $V_{7,2}=P\left(U_{7,2}\right)$ and $U_{8,2}=P^{-1}\left(V_{8,2}\right)$ to extend the fixed input-output pairs of *P*.

Note that once the items in $V_{3}=P\left(U_{3}\right)$ are fixed, the corresponding items in $U_{4}=P^{-1}\left(V_{4}\right)$ are uniquely determined since these two sets are both derived from ${\bar{Q}}_{X_{1}}$. Similarly, the items in $V_{5}=P\left(U_{5}\right)$ (resp. $V_{7,2}=P\left(U_{7,2}\right)$) uniquely determine the items in $U_{6}=P^{-1}\left(V_{6}\right)$ (resp. $U_{8,2}=P^{-1}\left(V_{8,2}\right)$). Then we sample all possible items for these sets through three steps.

$\mathrm{Step}\;I.\;\mathrm{Construct}\;V_{3}=P\left(U_{3}\right)\;\mathrm{and}\;U_{4}=P^{-1}\left(V_{4}\right)$.

Let $U^{3+}=U^{++}\cup U_{7,1}^{\prime }$ and $V^{3+}=V^{++}\cup V_{8,1}^{\prime }$. The size of $U^{3+}$ is $\Delta_{1}=p+{\bar{\alpha }}_{1}+{\bar{\alpha }}_{2}+\alpha_{3}+\alpha_{5}+{\bar{q}}^{\prime }+\alpha$, and the size of $V^{3+}$ is $\Delta_{2}=p+{\bar{\alpha }}_{1}+{\bar{\alpha }}_{2}+\alpha_{4}+\alpha_{6}+{\bar{q}}^{\prime }+\alpha$. Recall that ${\bar{X}}_{u}^{1}=\left\{\left(t,x,y\right)\in {\bar{Q}}_{F}:x⊕f_{1}\left(t\right)=u\right\}$ and ${\bar{X}}_{u}^{2}=\left\{\left(t,x,y\right)\in {\bar{Q}}_{F}:x⊕f_{2}\left(t\right)=u\right\}$. Let $N_{1}\left(\alpha \right)$ be the number of distinct tuples $\left(v_{3,1},\ldots ,v_{3,\alpha_{3}}\right)$ in ${\left\{0,1\right\}}^{n}\backslash V^{3+}$ such that the following two conditions are satisfied

(i) $\forall k\in [\alpha_{3}]$, for each $\left(t,x,y\right)\in {\bar{X}}_{u_{3,k}}^{1}$ where $u_{3,k}\in U_{3}$, $v_{3,k}⊕f_{1}\left(t\right)⊕f_{2}\left(t\right)⊕y\notin U^{3+}$.
(ii) $\forall k^{\prime },k\in \left[\alpha_{3}\right]$ with $k^{\prime }<k$, for each $\left(t,x,y\right)\in {\bar{X}}_{u_{3,k}}^{1}$, $v_{3,k}⊕f_{1}\left(t\right)⊕f_{2}\left(t\right)⊕y\neq v_{3,k^{\prime }}⊕f_{1}\left(t^{\prime }\right)⊕f_{2}\left(t^{\prime }\right)⊕y^{\prime }$ should be satisfied for each $\left(t^{\prime },x^{\prime },y^{\prime }\right)\in {\bar{X}}_{u_{3,k^{\prime }}}^{1}$.

Now we count the number of all possible distinct tuples $\left(v_{3,1},\ldots ,v_{3,\alpha_{3}}\right)\in {\left\{0,1\right\}}^{n}\backslash V^{3+}$ satisfying these two conditions. First, one has ${|\left\{0,1\right\}}^{n}\backslash V^{3+}|=2^{n}-\left(p+{\bar{\alpha }}_{1}+{\bar{\alpha }}_{2}+\alpha_{4}+\alpha_{6}+{\bar{q}}^{\prime }+\alpha \right)$. The first condition can remove at most $\left(p+{\bar{\alpha }}_{1}+{\bar{\alpha }}_{2}+\alpha_{3}+\alpha_{5}+{\bar{q}}^{\prime }+\alpha \right)\left|{\bar{X}}_{u_{3,k}}^{1}\right|$ items for each *k*, and the second condition can exclude at most $|{\bar{X}}_{u_{3,k}}^{1}\left|(\right|{\bar{X}}_{u_{3,1}}^{1}\left|+\ldots +\right|{\bar{X}}_{u_{3,k-1}}^{1}\left|\right)\leq \alpha_{4}\cdot \left|{\bar{X}}_{u_{3,k}}^{1}\right|$ values for each choice of $v_{3,k}$. By the choice of $v_{3,k}$ above, we obtain that

(35) $$\begin{array}{c} N_{1}\left(\alpha \right)\geq \prod_{k=0}^{\alpha_{3}-1}\left(2^{n}-\Delta_{2}-k-\left(\Delta_{1}+\alpha_{4}\right)\cdot \left|{\bar{X}}_{u_{3,k+1}}^{1}\right|\right). \end{array}$$

Let $V_{3}=\left\{v_{3,1},\ldots ,v_{3,\alpha_{3}}\right\}$ and $U_{4}=\left\{v_{3,k}⊕f_{1}\left(t\right)⊕f_{2}\left(t\right)⊕y:k\in \left[\alpha_{3}\right],\left(t,x,y\right)\in {\bar{X}}_{u_{3,k}}^{1}\right\}$. The first condition ensures that $U_{4}$ is disjoint with $U^{3+}$. Items in $U_{4}$ are distinct due to the second condition and the fact $\bar{\tau }\notin \Gamma_{8}^{\prime }$. This fact tells us that for each $k\in [\alpha_{3}]$ and $\left(t,x,y\right)\neq \left(t^{\prime },x^{\prime },y^{\prime }\right)\in {\bar{X}}_{u_{3,k}}^{1}$, it holds that $x⊕f_{1}\left(t\right)=x^{\prime }⊕f_{1}\left(t^{\prime }\right)=u_{3,k}$ but $f_{1}\left(t\right)⊕f_{2}\left(t\right)⊕y\neq f_{1}\left(t^{\prime }\right)⊕f_{2}\left(t^{\prime }\right)⊕y^{\prime }$, which means that $v_{3,k}⊕f_{1}\left(t\right)⊕f_{2}\left(t\right)⊕y\neq v_{3,k}⊕f_{1}\left(t^{\prime }\right)⊕f_{2}\left(t^{\prime }\right)⊕y^{\prime }$. Moreover, items in $V_{3}$ are distinct, and $V_{3}$ is disjoint with $V^{3+}$ by the choice of $\left(v_{3,1},\ldots ,v_{3,\alpha_{3}}\right)$. Let $U^{4+}=U^{3+}\cup U_{4}$, and $V^{4+}=V^{3+}\cup V_{3}$. The size of $U^{4+}$ is $\Delta_{3}=\Delta_{1}+\alpha_{4}$, and the size of $V^{4+}$ is $\Delta_{4}=\Delta_{2}+\alpha_{3}$.

$\mathrm{Step}\;II.\;\mathrm{Construct}\;V_{5}=P\left(U_{5}\right),\;\mathrm{and}\;U_{6}=P^{-1}\left(V_{6}\right)$.

Recall that $V_{6}=\left\{v_{6,1},\ldots ,v_{6,\alpha_{6}}\right\}$. Let $N_{2}\left(\alpha \right)$ be the number of all distinct tuples $\left(u_{6,1},\ldots ,u_{6,\alpha_{6}}\right)$ in ${\left\{0,1\right\}}^{n}\backslash U^{4+}$ satisfying the following two conditions:

(i) $\forall k\in [\alpha_{6}]$, for each $\left(t,x,y\right)\in {\bar{X}}_{v_{6,k}}^{2}$, $u_{6,k}⊕f_{1}\left(t\right)⊕f_{2}\left(t\right)⊕y\notin V^{4+}$.
(ii) $\forall k^{\prime },k\in \left[\alpha_{6}\right]$ with $k^{\prime }<k$, for each $\left(t,x,y\right)\in X_{v_{6,k}}^{2}$, $u_{6,k}⊕f_{1}\left(t\right)⊕f_{2}\left(t\right)⊕y\neq u_{6,k^{\prime }}⊕f_{1}\left(t^{\prime }\right)⊕f_{2}\left(t^{\prime }\right)⊕y^{\prime }$ should be satisfied for each $\left(t^{\prime },x^{\prime },y^{\prime }\right)\in X_{v_{6,k^{\prime }}}^{2}$.

Now we count the number of all possible distinct tuples $\left(u_{6,1},\ldots ,u_{6,\alpha_{6}}\right)\in {\left\{0,1\right\}}^{n}\backslash U^{4+}$ satisfying these two conditions. Similarly, one has ${|\left\{0,1\right\}}^{n}\backslash U^{4+}|=2^{n}-\left(p+{\bar{\alpha }}_{1}+{\bar{\alpha }}_{2}+\alpha_{3}+\alpha_{5}+{\bar{q}}^{\prime }+\alpha +\alpha_{4}\right)$. The first condition can remove at most $\left(p+{\bar{\alpha }}_{1}+{\bar{\alpha }}_{2}+\alpha_{4}+\alpha_{6}+{\bar{q}}^{\prime }+\alpha +\alpha_{3}\right)\cdot \left|{\bar{X}}_{v_{6,k}}^{2}\right|$ values for each *k*, and the second condition can exclude at most $\left(\right|{\bar{X}}_{v_{6,1}}^{2}\left|+\ldots +\right|{\bar{X}}_{v_{6,k-1}}^{2}\left|)\cdot \right|{\bar{X}}_{v_{6,k}}^{2}|\leq \alpha_{5}\cdot \left|{\bar{X}}_{v_{6,k}}^{2}\right|$ items for each choice of $u_{6,k}$. By the choice of ${\left(u_{6,k}\right)}_{k\in [\alpha_{6}]}$, we obtain that

(36) $$\begin{array}{c} N_{2}\left(\alpha \right)\geq \prod_{k=0}^{\alpha_{6}-1}\left(2^{n}-\Delta_{3}-k-\left(\Delta_{4}+\alpha_{5}\right)\cdot \left|{\bar{X}}_{v_{6,k}}^{2}\right|\right). \end{array}$$

Let $U_{6}=P^{-1}\left(V_{6}\right)\overset{def}{=}\left\{u_{6,1},\ldots ,u_{6,\alpha_{6}}\right\}$, and $V_{5}=P\left(U_{5}\right)\overset{def}{=}\left\{u_{6,k}⊕f_{1}\left(t\right)⊕f_{2}\left(t\right)⊕y:k\in \left[\alpha_{6}\right],\left(t,x,y\right)\in X_{v_{6,k}}^{2}\right\}$. It holds that items in $P^{-1}\left(V_{6}\right)$ are distinct. Furthermore, $P^{-1}\left(V_{6}\right)$ is disjoint with $U^{4+}$ by the choice of $\left(u_{6,1},\ldots ,u_{6,\alpha_{6}}\right)$. Let $U^{5+}=U^{4+}\cup U_{6}$, and $V^{5+}=V^{4+}\cup V_{5}$. The size of $U^{5+}$ is $\Delta_{5}=\Delta_{3}+\alpha_{6}$, and the size of $V^{5+}$ is $\Delta_{6}=\Delta_{4}+\alpha_{5}$.

$\mathrm{Step}\;III.\;\mathrm{Construct}\;V_{7,2}=P\left(U_{7,2}\right),\;\mathrm{and}\;U_{8,2}=P^{-1}\left(V_{8,2}\right)$.

Let ${\bar{q}}^{\prime \prime }={\bar{q}}^{\prime }-2\alpha$ (${\bar{q}}^{\prime \prime }=|U_{7,2}\left|=\right|V_{8,2}|$). Let *m* be the number of all distinct tweaks appearing in ${\bar{Q}}_{F}$, and then we use ${\bar{t}}_{1},\ldots ,{\bar{t}}_{m}$ to denote these *m* distinct tweaks. We denote $\tilde{Q_{0,i}}=\left\{\left({\bar{t}}_{i},x,y\right)\in {\bar{Q}}_{0}:x⊕f_{1}\left({\bar{t}}_{i}\right)\in U_{7,2}\wedge x⊕f_{2}\left({\bar{t}}_{i}\right)\in V_{8,2}\right\}$ and ${\bar{q}}_{i}^{\prime \prime }=\left|\tilde{Q_{0,i}}\right|$. In this case, it holds that ${\bar{q}}^{\prime \prime }=\sum_{i=1}^{m}{\bar{q}}_{i}^{\prime \prime }$. For convenience to count, we denote $\tilde{Q_{0}}={⋃}_{i=1}^{m}\tilde{Q_{0,i}}$ and rewrite the items in $\tilde{Q_{0}}$ indexed by the *m* distinct tweaks as

$$\begin{array}{c} \tilde{Q_{0}}=\left\{\left({\bar{t}}_{1},x_{1,1},y_{1,1}\right),\ldots ,\left({\bar{t}}_{1},x_{1,{\bar{q}}_{1}^{\prime \prime }},y_{1,{\bar{q}}_{1}^{\prime \prime }}\right),\ldots ,\left({\bar{t}}_{m},x_{m,1},y_{m,1}\right),\ldots ,\left({\bar{t}}_{m},x_{m,{\bar{q}}_{m}^{\prime \prime }},y_{m,{\bar{q}}_{m}^{\prime \prime }}\right)\right\}. \end{array}$$

For i=1,…,m and $j=1,\ldots ,{\bar{q}}_{i}^{\prime \prime }$, denote

$$\begin{array}{cc} \; & u_{7,i,j}=x_{i,j}⊕f_{1}\left({\bar{t}}_{i}\right)\;\mathrm{and}\;v_{8,i,j}=x_{i,j}⊕f_{2}\left({\bar{t}}_{i}\right). \end{array}$$

For convenience, $U_{7,2}$ and $V_{8,2}$ can be written as $U_{7,2}={\left\{u_{7,i,j}\right\}}_{1\leq i\leq m,1\leq j\leq {\bar{q}}_{i}^{\prime \prime }}$ and $V_{8,2}={\left\{v_{8,i,j}\right\}}_{1\leq i\leq m,1\leq j\leq {\bar{q}}_{i}^{\prime \prime }}$, respectively. Let ${\left(v_{7,i,j}\right)}_{1\leq i\leq m,1\leq j\leq {\bar{q}}_{i}^{\prime \prime }}$ be all possible different tuples in ${\left\{0,1\right\}}^{n}\backslash V^{5+}$ such that the following two conditions are satisfied.

(i) For each i=1,…,m and $j=1,\ldots ,{\bar{q}}_{i}^{\prime \prime }$, $v_{7,i,j}⊕f_{1}\left({\bar{t}}_{i}\right)⊕f_{2}\left({\bar{t}}_{i}\right)⊕y_{i,j}\notin U^{5+}$.
(ii) For each i=1,…,m and $j=1,\ldots ,{\bar{q}}_{i}^{\prime \prime }$, $v_{7,i,j}⊕f_{1}\left({\bar{t}}_{i}\right)⊕f_{2}\left({\bar{t}}_{i}\right)⊕y_{i,j}$ is distinct from the values $v_{7,k,l}⊕f_{1}\left({\bar{t}}_{k}\right)⊕f_{2}\left({\bar{t}}_{k}\right)⊕y_{k,l}$ for k<i and $l\in [{\bar{q}}_{k}^{\prime \prime }]$. Furthermore, $v_{7,i,j}⊕f_{1}\left({\bar{t}}_{i}\right)⊕f_{2}\left({\bar{t}}_{i}\right)⊕y_{i,j}$ should be distinct from the values $v_{7,i,j^{\prime }}⊕f_{1}\left({\bar{t}}_{i}\right)⊕f_{2}\left({\bar{t}}_{i}\right)⊕y_{i,j^{\prime }}$ for $j^{\prime }\in \left[{\bar{q}}_{i}^{\prime \prime }\right]$ with $j^{\prime }<j$.

Except these two conditions, each $v_{7,i,j}$ must be different from each other. By a simple computation, one has $|V^{5+}\left|=\right|U^{5+}|=p^{\prime }+{\bar{q}}^{\prime }+\alpha$, where $p^{\prime }=p+{\bar{\alpha }}_{1}+{\bar{\alpha }}_{2}+\alpha_{3}+\alpha_{4}+\alpha_{5}+\alpha_{6}$ and ${\bar{q}}^{\prime }=q-\left({\bar{\alpha }}_{1}+{\bar{\alpha }}_{2}+\alpha_{4}+\alpha_{5}\right)$. So ${|\left\{0,1\right\}}^{n}\backslash V^{5+}|=2^{n}-\left(p^{\prime }+{\bar{q}}^{\prime }+\alpha \right)$. Now we bound the number of all possible distinct tuples ${\left(v_{7,i,j}\right)}_{1\leq i\leq m,1\leq j\leq {\bar{q}}_{i}^{\prime \prime }}$ satisfying these two conditions. The first condition excludes at most $p^{\prime }+{\bar{q}}^{\prime }+\alpha$ values, and the second condition excludes at most $\sum_{k=1}^{i-1}{\bar{q}}_{k}^{\prime \prime }-j+1$ values for each choice of $v_{7,i,j}$. Furthermore, $v_{7,i,j}$ should not be same as any one of previous $\sum_{k=1}^{i-1}{\bar{q}}_{k}^{\prime \prime }-j+1$ items. By combining these facts, one can conclude that

(37) $$\begin{array}{cc} N_{0}\left(\alpha \right) & \geq \prod_{i=1}^{m}\prod_{j=0}^{{\bar{q}}_{i}^{\prime \prime }-1}\left(2^{n}-2p^{\prime }-2{\bar{q}}^{\prime }-2\alpha -2\sum_{k=1}^{i-1}{\bar{q}}_{k}^{\prime \prime }-2j\right). \end{array}$$

Overall, by combining (33), (35), (36), and (37), one has

(38) $$\begin{array}{c} p^{\prime \prime }\left(\bar{\tau }\right)=\sum_{0\leq \alpha \leq M}\frac{N_{S}\left(\alpha \right)\cdot N_{1}\left(\alpha \right)\cdot N_{2}\left(\alpha \right)\cdot N_{0}\left(\alpha \right)}{{\left(2^{n}-p-{\bar{\alpha }}_{1}-{\bar{\alpha }}_{2}\right)}_{\alpha_{3}+\alpha_{4}+\alpha_{5}+\alpha_{6}+2{\bar{q}}^{\prime \prime }+3\alpha }}. \end{array}$$

By combining (32) and (38), we have

(39) $$\begin{array}{c} p\left(\bar{\tau }\right)=\sum_{0\leq \alpha \leq M}\frac{N_{S}\left(\alpha \right)\cdot N_{1}\left(\alpha \right)\cdot N_{2}\left(\alpha \right)\cdot N_{0}\left(\alpha \right)}{{\left(2^{n}-p\right)}_{{\bar{\alpha }}_{1}+{\bar{\alpha }}_{2}+\alpha_{3}+\alpha_{4}+\alpha_{5}+\alpha_{6}+2{\bar{q}}^{\prime \prime }+3\alpha }}. \end{array}$$

Recall that

(40) $$\frac{\mathrm{Pr}[T_{\mathrm{re}}=\bar{\tau }]}{\mathrm{Pr}[T_{\mathrm{id}}=\bar{\tau }]}=2^{nq}p\left(\bar{\tau }\right).$$

By combining (39) and (40), we conclude that

(41) $$\begin{array}{cc} \frac{\mathrm{Pr}[T_{\mathrm{re}}=\bar{\tau }]}{\mathrm{Pr}[T_{\mathrm{id}}=\bar{\tau }]} & \geq \sum_{0\leq \alpha \leq M}\frac{2^{nq}\cdot N_{S}\left(\alpha \right)\cdot N_{1}\left(\alpha \right)\cdot N_{2}\left(\alpha \right)\cdot N_{0}\left(\alpha \right)}{{\left(2^{n}-p\right)}_{{\bar{\alpha }}_{1}+{\bar{\alpha }}_{2}+\alpha_{3}+\alpha_{4}+\alpha_{5}+\alpha_{6}+2q^{\prime \prime }+3\alpha }}\\ \; & =\sum_{0\leq \alpha \leq M}\underset{R_{1}\left(\alpha \right)}{\underset{︸}{\frac{N_{1}\left(\alpha \right)}{{\left(2^{n}-p\right)}_{\alpha_{3}}}}}\cdot \underset{R_{2}\left(\alpha \right)}{\underset{︸}{\frac{N_{2}\left(\alpha \right)}{{\left(2^{n}-p-\alpha_{3}\right)}_{\alpha_{6}}}}}\\ \; & \;\;\;\;\cdot \underset{\geq 1(*)}{\underset{︸}{\frac{2^{n(q-{\bar{q}}^{\prime })}}{{\left(2^{n}-p-\alpha_{3}-\alpha_{6}\right)}_{{\bar{\alpha }}_{1}+{\bar{\alpha }}_{2}+\alpha_{4}+\alpha_{5}}}}}\\ \; & \;\;\;\;\cdot \underset{R_{0}\left(\alpha \right)}{\underset{︸}{\frac{2^{n{\bar{q}}^{\prime }}\cdot N_{S}\left(\alpha \right)\cdot N_{0}\left(\alpha \right)}{{\left(2^{n}-p-{\bar{\alpha }}_{1}-{\bar{\alpha }}_{2}-\alpha_{3}-\alpha_{4}-\alpha_{5}-\alpha_{6}\right)}_{2{\bar{q}}^{\prime \prime }+3\alpha }}}}, \end{array}$$

where (*) follows as $q-{\bar{q}}^{\prime }={\bar{\alpha }}_{1}+{\bar{\alpha }}_{2}+\alpha_{4}+\alpha_{5}$.

Lower bounds on $R_{1}\left(\alpha \right)$, $R_{2}\left(\alpha \right)$, and $R_{0}\left(\alpha \right)$ are given in Appendix D, and the results are showed as follows:

(42) $$R_{1}\left(\alpha \right)\geq 1-\epsilon_{1},\;\mathrm{where}\;\epsilon_{1}=\frac{8q^{3/2}}{2^{n}}+\frac{2p\sqrt{q}}{2^{n}}+\frac{4q}{2^{n}}.$$

(43) $$R_{2}\left(\alpha \right)\geq 1-\epsilon_{2},\;\mathrm{where}\;\epsilon_{2}=\frac{8q^{3/2}}{2^{n}}+\frac{2p\sqrt{q}}{2^{n}}+\frac{4q}{2^{n}}.$$

(44) $$R_{0}\left(\alpha \right)\geq \left(1-\epsilon_{0}\right)\cdot \left(1-\epsilon_{3}\right)\cdot \left(1-\epsilon_{4}\right)\cdot {\mathrm{Hyp}}_{2^{n}-p^{\prime },{\bar{q}}^{\prime },{\bar{q}}^{\prime }}\left(\alpha \right),$$

where $\epsilon_{0}=\frac{6q}{2^{2n/3}}+\frac{16\sqrt{q}}{2^{n/3}}$, $\epsilon_{3}=\frac{4q}{2^{2n/3}}$, and $\epsilon_{4}=\frac{4q{\left(p+2q+6\sqrt{q}\right)}^{2}}{2^{2n}}$.

Putting (42), (43), and (44) into (41), we obtain

(45) $$\begin{array}{cc} \frac{\mathrm{Pr}[T_{\mathrm{re}}=\bar{\tau }]}{\mathrm{Pr}[T_{\mathrm{id}}=\bar{\tau }]} & \geq \left(1-\epsilon_{0}\right)\left(1-\epsilon_{1}\right)\left(1-\epsilon_{2}\right)\left(1-\epsilon_{3}\right)\left(1-\epsilon_{4}\right)\sum_{0\leq \alpha \leq M}{\mathrm{Hyp}}_{2^{n}-p^{\prime },{\bar{q}}^{\prime },{\bar{q}}^{\prime }}\left(\alpha \right). \end{array}$$

The last term in (45) can be bounded as

(46) $$\begin{array}{cc} \sum_{0\leq \alpha \leq M}{\mathrm{Hyp}}_{2^{n}-p^{\prime },{\bar{q}}^{\prime },{\bar{q}}^{\prime }}\left(\alpha \right) & =1-\sum_{\alpha >{\bar{q}}^{\prime }/2^{n/3}}{\mathrm{Hyp}}_{2^{n}-p^{\prime },{\bar{q}}^{\prime },{\bar{q}}^{\prime }}\left(\alpha \right)\\ \; & \overset{\left(v\right)}{\geq }\left(1-\frac{E[{\mathrm{Hyp}}_{2^{n}-p^{\prime },{\bar{q}}^{\prime },{\bar{q}}^{\prime }}\left(\alpha \right)]}{{\bar{q}}^{\prime }/2^{n/3}}\right)\\ \; & =\left(1-\frac{{\left({\bar{q}}^{\prime }\right)}^{2}}{\left(2^{n}-p^{\prime }\right){\bar{q}}^{\prime }/2^{n/3}}\right)\\ \; & =\left(1-\frac{{\bar{q}}^{\prime }\cdot 2^{\frac{n}{3}}}{2^{n}-p^{\prime }}\right)\\ \; & \overset{\left(vi\right)}{\geq }\left(1-\frac{2q}{2^{2n/3}}\right), \end{array}$$

where (v) follows as Markov’s inequality and (vi) follows as $2^{n}-p^{\prime }\geq 2^{n}-p-6\sqrt{q}\geq 2^{n-1}$ which comes from the assumption $p+6\sqrt{q}\leq p+6\sqrt{q}+2q\leq 2^{n-1}$ and the fact ${\bar{q}}^{\prime }\leq q$. Let $\epsilon_{5}=\frac{2q}{2^{2n/3}}$. Then we can write (45) as

(47) $$\begin{array}{cc} \frac{\mathrm{Pr}[T_{\mathrm{re}}=\bar{\tau }]}{\mathrm{Pr}[T_{\mathrm{id}}=\bar{\tau }]} & \geq \left(1-\epsilon_{0}\right)\left(1-\epsilon_{1}\right)\left(1-\epsilon_{2}\right)\left(1-\epsilon_{3}\right)\left(1-\epsilon_{4}\right)\left(1-\epsilon_{5}\right)\\ \; & \geq (1-\epsilon_{0}-\epsilon_{1}-\epsilon_{2}-\epsilon_{3}-\epsilon_{4}-\epsilon_{5}). \end{array}$$

Combing all these facts together, the proof of Lemma 6 is finished.  □

Finally, by Lemmas 1, 5 and 6, Theorem 2 follows.  □

## 5. Conclusions

In this paper, we first prove the BBB security of the construction SoEM22 in the multi-key setting, and further tweak this construction. When the bidirectionally efficient public random permutations are considered, we build the parallelizable beyond-birthday secure PRFs from one permutation in the multi-key setting, and also tweak this new construction while preserving BBB security. By a slight modification of two tweakable PRFs, we obtain two parallelizable nonce based MACs for variable length messages. In fact, the constructions mentioned above come from sum of two Even-Mansours. It is natural to generalize SoEM22 to sum of *s* Even-Mansours, namely

$$\begin{array}{c} F_{K_{1},\ldots ,K_{s}}^{P_{1},\ldots ,P_{s}}\left(x\right)=P_{1}\left(x⊕K_{1}\right)⊕K_{1}⊕\cdots ⊕P_{s}\left(x⊕K_{s}\right)⊕K_{s}, \end{array}$$

where $P_{1},\ldots ,P_{s}\overset{\$}{\leftarrow }\mathrm{Perm}\left(n\right)$ are *s* independent random permutations, and $K_{1},\ldots ,K_{s}$ are *s* *n*-bit uniformly random strings. Obliviously, this generalization is at least as secure as SoEM22 even in the multi-key setting. However, the detailed analysis of its security is not easy to see, and we leave it as a future work.

## Data Availability

Not applicable.

## Appendix A. Upper Bound on $\mathrm{Pr}[T_{\mathrm{id}}\in \Gamma_{\mathrm{bad}}]$ in Lemma 3

For each i∈[13], we upper bound $\mathrm{Pr}[T_{\mathrm{id}}\in \Gamma_{i}]$ as follows.

Bounding (B-1), (B-2), and (B-3): First, we consider (B-1). For any $\left(t_{i},x_{i},y_{i}\right)\in Q_{F}$, $u_{1,j}\in U_{1}$, and $u_{2,j^{\prime }}\in U_{2}$, by the $\epsilon_{1}$-regular property of $\left(f_{1},f_{2}\right)$, one has

$$\mathrm{Pr}\left[\left(f_{1}\left(t_{i}\right)=x_{i}⊕u_{1,j}\right)\wedge \left(f_{2}\left(t_{i}\right)=x_{i}⊕u_{2,j^{\prime }}\right)\right]\leq \epsilon_{1}^{2}.$$

Since the number of all possible tuples for ${\left(\left(t_{i},x_{i},y_{i}\right),u_{1,j},u_{2,j^{\prime }}\right)}_{i\in \left[q\right],j\in \left[p_{1}\right],j^{\prime }\in \left[p_{2}\right]}$ is $qp_{1}p_{2}$, by union bound, it holds that

$$\mathrm{Pr}\left[T_{\mathrm{id}}\in \Gamma_{1}\right]\leq qp_{1}p_{2}\epsilon_{1}^{2}.$$

Similarly, we can bound the probabilities of (B-2) and (B-3) as

$$\mathrm{Pr}\left[T_{\mathrm{id}}\in \Gamma_{2}\right]\leq qp_{1}p_{2}\epsilon_{1}^{2}\;\mathrm{and}\;\mathrm{Pr}\left[T_{\mathrm{id}}\in \Gamma_{3}\right]\leq qp_{1}p_{2}\epsilon_{1}^{2}.$$

Bounding (B-4) and (B-5): For any two distinct queries $\left(t_{i},x_{i},y_{i}\right)\neq \left(t_{i^{\prime }},x_{i^{\prime }},y_{i^{\prime }}\right)\in Q_{F}$, by the $\epsilon_{2}$-AXU property of pair $\left(f_{1},f_{2}\right)$, we have

$$\mathrm{Pr}\left[\left(f_{1}\left(t_{i}\right)⊕f_{1}\left(t_{i^{\prime }}\right)=x_{i}⊕x_{i^{\prime }}\right)\wedge \left(f_{2}\left(t_{i}\right)⊕f_{2}\left(t_{i^{\prime }}\right)=f_{1}\left(t_{i}\right)⊕f_{1}\left(t_{i^{\prime }}\right)⊕y_{i}⊕y_{i^{\prime }}\right)\right]\leq \epsilon_{2}^{2}.$$

Since there are q(q−1)/2 possible unordered pairs for ${\left\{\left(t_{i},x_{i},y_{i}\right),\left(t_{i^{\prime }},x_{i^{\prime }},y_{i^{\prime }}\right)\right\}}_{i\neq i^{\prime }\in \left[q\right]}$, by union bound, one can obtain that

$$\mathrm{Pr}\left[T_{\mathrm{id}}\in \Gamma_{4}\right]\leq \frac{\epsilon_{2}^{2}q^{2}}{2},\;\mathrm{and}\;\mathrm{similarly},\;\mathrm{Pr}\left[T_{\mathrm{id}}\in \Gamma_{5}\right]\leq \frac{\epsilon_{2}^{2}q^{2}}{2}.$$

Bounding (B-6) and (B-7): For any two distinct construction queries $\left(t_{i},x_{i},y_{i}\right)\neq \left(t_{i^{\prime }},x_{i^{\prime }},y_{i^{\prime }}\right)\in Q_{F}$ and any $u_{1,j}\in U_{1}$, by the $\epsilon_{1}$-regular and $\epsilon_{2}$-AXU properties of $\left(f_{1},f_{2}\right)$, we have

$$\mathrm{Pr}\left[\left(f_{1}\left(t_{i}\right)=x_{i}⊕u_{1,j}\right)\wedge \left(f_{2}\left(t_{i}\right)⊕f_{2}\left(t_{i^{\prime }}\right)=x_{i}⊕x_{i^{\prime }}\right)\right]\leq \epsilon_{1}\epsilon_{2}.$$

Then, summing over all $\left(t_{i},x_{i},y_{i}\right)\neq \left(t_{i^{\prime }},x_{i^{\prime }},y_{i^{\prime }}\right)\in Q_{F}$ and $u_{1,j}\in U_{1}$, one has

$$\mathrm{Pr}\left[T_{\mathrm{id}}\in \Gamma_{6}\right]\leq \frac{\epsilon_{1}\epsilon_{2}q^{2}p_{1}}{2},\;\mathrm{and}\;\mathrm{similarly},\;\mathrm{Pr}\left[T_{\mathrm{id}}\in \Gamma_{7}\right]\leq \frac{\epsilon_{1}\epsilon_{2}q^{2}p_{2}}{2}.$$

Bounding (B-8): For any $\left(t_{i},x_{i},y_{i}\right),\left(t_{i^{\prime }},x_{i^{\prime }},y_{i^{\prime }}\right),\left(t_{i^{\prime \prime }},x_{i^{\prime \prime }},y_{i^{\prime \prime }}\right)\in Q_{F}$ with $\left(t_{i},x_{i},y_{i}\right)\neq \left(t_{i^{\prime }},x_{i^{\prime }},y_{i^{\prime }}\right)$ and $\left(t_{i},x_{i},y_{i}\right)\neq \left(t_{i^{\prime \prime }},x_{i^{\prime \prime }},y_{i^{\prime \prime }}\right)$, by the $\epsilon_{2}$-AXU property of $\left(f_{1},f_{2}\right)$, one concludes that

$$\mathrm{Pr}\left[\left(f_{1}\left(t_{i}\right)⊕f_{1}\left(t_{i^{\prime }}\right)=x_{i}⊕x_{i^{\prime }}\right)\wedge \left(f_{2}\left(t_{i}\right)⊕f_{2}\left(t_{i^{\prime \prime }}\right)=x_{i}⊕x_{i^{\prime \prime }}\right)\right]\leq \epsilon_{2}^{2}.$$

Note that the above inequality also holds for the case $t_{i}=t_{i^{\prime }}$ (resp. $t_{i}=t_{i^{\prime \prime }}$) since we have $x_{i}\neq x_{i^{\prime }}$ (resp. $x_{i}\neq x_{i^{\prime \prime }}$) i.e., $x_{i}⊕x_{i^{\prime }}=0$ (resp. $x_{i}⊕x_{i^{\prime \prime }}=0$). It is easy to count that the number of all possible $\left(t_{i},x_{i},y_{i}\right),\left(t_{i^{\prime }},x_{i^{\prime }},y_{i^{\prime }}\right),\left(t_{i^{\prime \prime }},x_{i^{\prime \prime }},y_{i^{\prime \prime }}\right)$ is at most $q^{3}$, which means that

$$\mathrm{Pr}\left[T_{\mathrm{id}}\in \Gamma_{8}\right]\leq \epsilon_{2}^{2}q^{3}.$$

Bounding (B-9), (B-10), (B-11), and (B-12): We deal with bad conditions (B-9) and (B-11) together by using the fact that

$$\mathrm{Pr}\left[T_{\mathrm{id}}\in \Gamma_{9}\cup \Gamma_{11}\right]\leq \mathrm{Pr}\left[T_{\mathrm{id}}\in \Gamma_{11}\right]+\mathrm{Pr}\left[T_{\mathrm{id}}\in \Gamma_{9}\backslash \Gamma_{11}\right].$$

We first consider how to upper bound $\mathrm{Pr}[T_{\mathrm{id}}\in \Gamma_{11}]$. For the random variable $\alpha_{1}=\left|\left\{\left(t,x,y\right)\in Q_{F}:x⊕f_{1}\left(t\right)\in U_{1}\right\}\right|$ (the randomness from the choice of $f_{1}$), its expectation value can be computed as

E[α1]≤∑(t,x,y)∈QF:∑u1∈U1:Pr[x⊕f1(t)=u1]≤ϵ1qp1,

due to the $\epsilon_{1}$-regular property of $\left(f_{1},f_{2}\right)$. By Markov’s inequality, one has

$$\mathrm{Pr}\left[T_{\mathrm{id}}\in \Gamma_{11}\right]\leq \frac{E[\alpha_{1}]}{\sqrt{q}}\leq \epsilon_{1}\sqrt{q}p_{1}.$$

Under the condition $\alpha_{1}\leq \sqrt{q}$, there are at most q/2 pairs $\{\left(\left(t_{i},x_{i},y_{i}\right),u_{1,j}\right)$, $(\left(t_{i^{\prime }},x_{i^{\prime }},y_{i^{\prime }}\right),\;u_{1,j^{\prime }})\}$ such that $x_{i}⊕f_{1}\left(t_{i}\right)=u_{1,j}$ and $x_{i^{\prime }}⊕f_{1}\left(t_{i^{\prime }}\right)=u_{1,j^{\prime }}$ where $\left(t_{i},x_{i},y_{i}\right)\neq \left(t_{i^{\prime }},x_{i^{\prime }},y_{i^{\prime }}\right)\in Q_{F}$ and $\left(u_{1,j},v_{1,j}\right),\left(u_{1,j^{\prime }},v_{1,j^{\prime }}\right)\in Q_{P_{1}}$. In this case, the corresponding $y_{i}$ and $y_{i^{\prime }}$ are two independently uniform random variables over ${\left\{0,1\right\}}^{n}$ so that we have

$$\mathrm{Pr}\left[v_{1,j}⊕f_{1}\left(t_{i}\right)⊕f_{2}\left(t_{i}\right)⊕y_{i}=v_{1,j^{\prime }}⊕f_{1}\left(t_{i^{\prime }}\right)⊕f_{2}\left(t_{i^{\prime }}\right)⊕y_{i^{\prime }}\right]\leq \frac{1}{2^{n}}.$$

By summing over all the q/2 possible pairs, one can obtain that

$$\mathrm{Pr}\left[T_{\mathrm{id}}\in \Gamma_{9}\backslash \Gamma_{11}\right]\leq \frac{q}{2^{n+1}}.$$

Finally, it holds that

$$\mathrm{Pr}\left[T_{\mathrm{id}}\in \Gamma_{9}\cup \Gamma_{11}\right]\leq \epsilon_{1}\sqrt{q}p_{1}+\frac{q}{2^{n+1}}.$$

Similarly, we obtain

$$\mathrm{Pr}\left[T_{\mathrm{id}}\in \Gamma_{10}\cup \Gamma_{12}\right]\leq \epsilon_{1}\sqrt{q}p_{2}+\frac{q}{2^{n+1}}.$$

Bounding (B-13): To bound $\mathrm{Pr}[\beta_{1}\geq \sqrt{q}]$, we first define the random variable $T_{F}=\left|\left\{\left(\left(t,x,y\right),\left(t^{\prime },x^{\prime },y^{\prime }\right)\right)\in Q_{F}\times Q_{F}:\left(t,x,y\right)\neq \left(t^{\prime },x^{\prime },y^{\prime }\right),x⊕f_{1}\left(t\right)=x^{\prime }⊕f_{1}\left(t^{\prime }\right)\right\}\right|$. By definition of $\beta_{1}$, one has

$$\beta_{1}=\left|\left\{\left(t,x,y\right)\in Q_{F}:\exists \left(t,x,y\right)\neq \left(t^{\prime },x^{\prime },y^{\prime }\right),x⊕f_{1}\left(t\right)=x^{\prime }⊕f_{1}\left(t^{\prime }\right)\right\}\right|\leq T_{F}.$$

Hence, $E\left[\beta_{1}\right]\leq E\left[T_{F}\right]$. We can compute the expectation value of $T_{F}$ as

E[TF]=∑(t,x,y)≠(t′,x′,y′):Pr[x⊕f1(t)=x′⊕f1(t′)]≤ϵ2q22

from the $\epsilon_{2}$-AXU property of $\left(f_{1},f_{2}\right)$. By Markov’s inequality, one has

$$\mathrm{Pr}\left[\beta_{1}\geq \sqrt{q}\right]\leq \frac{E[\beta_{1}]}{\sqrt{q}}\leq \frac{E[T_{F}]}{\sqrt{q}}\leq \frac{\epsilon_{2}q^{3/2}}{2},\;\mathrm{and}\;\mathrm{similarly},\;\mathrm{Pr}\left[\beta_{2}\geq \sqrt{q}\right]\leq \frac{\epsilon_{2}q^{3/2}}{2}.$$

Finally, we obtain

$$\mathrm{Pr}\left[T_{\mathrm{id}}\in \Gamma_{13}\right]=\mathrm{Pr}\left[\left(\beta_{1}\geq \sqrt{q}\right)\vee \left(\beta_{2}\geq \sqrt{q}\right)\right]\leq \epsilon_{2}q^{3/2}.$$

## Appendix B. More Details in Proof of Lemma 4

$\mathrm{Lower}\;\mathrm{Bounding}\;p^{\prime }\left(\tau \right)$. Conditioned on $P_{1}⊢Q_{P_{1}}$ and $P_{2}⊢Q_{P_{2}}$, $P_{1}$ (resp. $P_{2}$) is fixed on exactly $p_{1}$ (resp. $p_{2}$) input-output pairs. For each $\left(t,x,y\right)\in Q_{U_{1}}$, there exists a unique $\left(u_{1},v_{1}\right)\in Q_{P_{1}}$ satisfying $x⊕f_{1}\left(t\right)=u_{1}$ so that $P_{1}\left(x⊕f_{1}\left(t\right)\right)=P_{1}\left(u_{1}\right)=v_{1}$. Then we can define two corresponding multi-sets as:

$$\begin{array}{cc} \; & {\tilde{U}}_{2}=\left\{x⊕f_{2}\left(t\right):\left(t,x,y\right)\in Q_{U_{1}}\right\},\\ \; & {\tilde{V}}_{2}=\left\{P_{1}\left(x⊕f_{1}\left(t\right)\right)⊕f_{1}\left(t\right)⊕f_{2}\left(t\right)⊕y:\left(t,x,y\right)\in Q_{U_{1}}\right\}. \end{array}$$

Note that all values in ${\tilde{U}}_{2}$ (resp. ${\tilde{V}}_{2}$) are distinct since otherwise τ would satisfy (B-6) (resp. (B-9)). Then it holds that $|{\tilde{U}}_{2}\left|=\right|{\tilde{V}}_{2}\left|=\right|Q_{U_{1}}|=\alpha_{1}$. Moreover, since $\tau \notin \Gamma_{1}$ and $\tau \notin \Gamma_{2}$, one conclude that $U_{2}\cap {\tilde{U}}_{2}=\emptyset$ and $V_{2}\cap {\tilde{V}}_{2}=\emptyset$, respectively. Then we get

(A1) $$\begin{array}{c} \mathrm{Pr}\left[E_{U_{1}}\;|\;P_{2}⊢Q_{P_{2}}\right]=\frac{1}{{\left(2^{n}-p_{2}\right)}_{\alpha_{1}}}. \end{array}$$

Similarly, for each $\left(t,x,y\right)\in Q_{U_{2}}$, there exists a unique $\left(u_{2},v_{2}\right)\in Q_{P_{2}}$ satisfying $x⊕f_{2}\left(t\right)=u_{2}$, which means $P_{2}\left(x⊕f_{2}\left(t\right)\right)=v_{2}$. Then two corresponding multi-sets can be defined as:

$$\begin{array}{cc} \; & {\tilde{U}}_{1}=\left\{x⊕f_{1}\left(t\right):\left(t,x,y\right)\in Q_{U_{2}}\right\},\\ \; & {\tilde{V}}_{1}=\left\{P_{2}\left(x⊕f_{2}\left(t\right)\right)⊕f_{1}\left(t\right)⊕f_{2}\left(t\right)⊕y:\left(t,x,y\right)\in Q_{U_{2}}\right\}. \end{array}$$

All values in ${\tilde{U}}_{1}$ (resp. ${\tilde{V}}_{1}$) are distinct since otherwise τ would satisfy (B-7) (resp. (B-10)). Then one has $|{\tilde{U}}_{1}\left|=\right|{\tilde{V}}_{1}\left|=\right|Q_{U_{2}}|=\alpha_{2}$. Moreover, since $\tau \notin \Gamma_{1}$ and $\tau \notin \Gamma_{3}$, it holds that $U_{1}\cap {\tilde{U}}_{1}=\emptyset$ and $V_{1}\cap {\tilde{V}}_{1}=\emptyset$, respectively. Hence,

(A2) $$\begin{array}{c} \mathrm{Pr}\left[E_{U_{2}}\;|\;P_{1}⊢Q_{P_{1}}\right]=\frac{1}{{\left(2^{n}-p_{1}\right)}_{\alpha_{2}}}. \end{array}$$

By combing (A1) and (A2), one can conclude that

(A3) $$\begin{array}{c} p^{\prime }\left(\tau \right)=\frac{1}{{\left(2^{n}-p_{2}\right)}_{\alpha_{1}}{\left(2^{n}-p_{1}\right)}_{\alpha_{2}}}. \end{array}$$

Now it holds that $|{\tilde{U}}_{1}\left|=\right|{\tilde{V}}_{1}|=\alpha_{2}$ and $|{\tilde{U}}_{2}\left|=\right|{\tilde{V}}_{2}|=\alpha_{1}$. Then we define four disjoint collections $U_{1}\overset{\mathrm{def}}{=}\left(U_{1},{\tilde{U}}_{1}\right)$, $V_{1}\overset{\mathrm{def}}{=}\left(V_{1},{\tilde{V}}_{1}\right)$, $U_{2}\overset{\mathrm{def}}{=}\left(U_{2},{\tilde{U}}_{2}\right)$, and $V_{2}\overset{\mathrm{def}}{=}\left(V_{2},{\tilde{V}}_{2}\right)$. Notice that when conditioned on $E_{U_{1}}\wedge E_{U_{2}}\wedge \left(P_{i}⊢Q_{P_{i}},i=1,2\right)$, $P_{1}$ is fixed on exactly $p_{1}+\alpha_{2}$ input-output pairs and $P_{2}$ is fixed on exactly $p_{2}+\alpha_{1}$ input-output pairs.

$\mathrm{Lower}\;\mathrm{Bounding}\;p^{\prime \prime }\left(\tau \right)$. Conditioned on $E_{U_{1}}\wedge E_{U_{2}}\wedge \left(P_{i}⊢Q_{P_{i}},i=1,2\right)$ we next lower bound the number of all possible “new” and distinct input-output pairs of $P_{1}$ and $P_{2}$ such that the event $E_{X_{1}}\wedge E_{X_{2}}\wedge E_{0}$ happens. We first define four multi-sets derived from $Q_{X_{1}}$ and $Q_{X_{2}}$ as:

$$\begin{array}{cc} \; & U_{1,1}=\left\{x⊕f_{1}\left(t\right):\left(t,x,y\right)\in Q_{X_{1}}\right\},\;\;U_{2,1}=\left\{x⊕f_{1}\left(t\right):\left(t,x,y\right)\in Q_{X_{2}}\right\},\\ \; & U_{1,2}=\left\{x⊕f_{2}\left(t\right):\left(t,x,y\right)\in Q_{X_{1}}\right\},\;\;U_{2,2}=\left\{x⊕f_{2}\left(t\right):\left(t,x,y\right)\in Q_{X_{2}}\right\}. \end{array}$$

The size of four sets above can be denoted as $\alpha_{1,1}=\left|U_{1,1}\right|$, $\alpha_{1,2}=\left|U_{1,2}\right|$, $\alpha_{2,1}=\left|U_{2,1}\right|$, and $\alpha_{2,2}=\left|U_{2,2}\right|$. We also denote four additional sets as $V_{1,1}=P\left(U_{1,1}\right)$, $V_{1,2}=P\left(U_{1,2}\right)$, $V_{2,1}=P\left(U_{2,1}\right)$, and $V_{2,2}=P\left(U_{2,2}\right)$, which can be wrote more clearly as:

$$\begin{array}{cc} \; & V_{1,1}=\left\{P\left(x⊕f_{1}\left(t\right)\right):\left(t,x,y\right)\in Q_{X_{1}}\right\},\;\;V_{2,1}=\left\{P\left(x⊕f_{1}\left(t\right)\right):\left(t,x,y\right)\in Q_{X_{2}}\right\},\\ \; & V_{1,2}=\left\{P\left(x⊕f_{2}\left(t\right)\right):\left(t,x,y\right)\in Q_{X_{1}}\right\},\;\;V_{2,2}=\left\{P\left(x⊕f_{2}\left(t\right)\right):\left(t,x,y\right)\in Q_{X_{2}}\right\}. \end{array}$$

For convenience, we rewrite $U_{1,1}$ and $U_{2,2}$ as:

$$\begin{array}{cc} \; & U_{1,1}=\left\{u_{1,1},\ldots ,u_{1,\alpha_{1,1}}\right\},\;\;U_{2,2}=\left\{u_{2,1},\ldots ,u_{2,\alpha_{2,2}}\right\}. \end{array}$$

Recall that $D_{1}=\left\{x⊕f_{1}\left(t\right):\left(t,x,y\right)\in Q_{F}\right\}$ and $D_{2}=\left\{x⊕f_{2}\left(t\right):\left(t,x,y\right)\in Q_{F}\right\}$. Then $\alpha_{1,1}$ and $\alpha_{1,2}$ can be bounded as:

α1,1≤∑x∈{0,1}n:δD1(x)>11≤∑x∈{0,1}n:δD1(x)>1δD1(x)2=β12≤q2,α1,2≤∑i=1α1,1δD1(u1,i)≤∑x∈{0,1}n:δD1(x)>1δD1(x)=β1≤q.

Similarly, we obtain $\alpha_{2,2}\leq \frac{\sqrt{q}}{2}$ and $\alpha_{2,1}\leq \sqrt{q}$. From the fact $\tau \notin \Gamma_{8}$, one has that any items in the $U_{1,2}$ (resp.$U_{2,1}$) are distinct so that $\alpha_{1,2}=\left|Q_{X_{1}}\right|$ (resp. $\alpha_{2,1}=\left|Q_{X_{2}}\right|$) holds. Finally, we define two multi-sets derived from $Q_{0}$ as

$$\begin{array}{c} U_{0}^{1}=\left\{x⊕f_{1}\left(t\right):\left(t,x,y\right)\in Q_{0}\right\}\;\mathrm{and}\;U_{0}^{2}=\left\{x⊕f_{2}\left(t\right):\left(t,x,y\right)\in Q_{0}\right\}. \end{array}$$

Due to the definition of $Q_{0}$, it holds that any items in $U_{0}^{1}$ (resp. $U_{0}^{2}$) are distinct. We can also denote two additional sets as

$$\begin{array}{c} V_{0}^{1}=P\left(U_{0}^{1}\right)=\left\{P\left(x⊕f_{1}\left(t\right)\right):\left(t,x,y\right)\in Q_{0}\right\},\\ V_{0}^{2}=P\left(U_{0}^{2}\right)=\left\{P\left(x⊕f_{2}\left(t\right)\right):\left(t,x,y\right)\in Q_{0}\right\}. \end{array}$$

Let $q^{\prime }\overset{\mathrm{def}}{=}|Q_{0}\left|=q-(\right|Q_{U}\left|+\right|Q_{V}\left|+\right|Q_{X_{1}}\left|+\right|Q_{X_{2}}\left|\right)=q-\left(\alpha_{1}+\alpha_{2}+\alpha_{1,2}+\alpha_{2,1}\right)$ (besides, $q^{\prime }=|U_{0}^{1}\left|=\right|U_{0}^{2}|$). Let *m* be the number of all distinct tweaks appearing in $Q_{F}$, and then we use ${\hat{t}}_{1},\ldots ,{\hat{t}}_{m}$ to denote these *m* distinct tweaks. Furthermore, write $Q_{0,i}$ as a set consisting of all the query-response tuples indexed by the tweak ${\hat{t}}_{i}$ in $Q_{0}$ and denote $q_{i}^{\prime }=\left|Q_{0,i}\right|$ ($q_{i}^{\prime }$ might be zero for some *i*). Then it holds that $Q_{0}={⋃}_{i=1}^{m}Q_{0,i}$ and respectively $q^{\prime }=\sum_{i=1}^{m}q_{i}^{\prime }$. For convenience to count, we rearrange the items in $Q_{0}$ as

$$\begin{array}{c} Q_{0}=\left\{\left({\hat{t}}_{1},x_{1,1},y_{1,1}\right),\ldots ,\left({\hat{t}}_{1},x_{1,q_{1}^{\prime }},y_{1,q_{1}^{\prime }}\right),\ldots ,\left({\hat{t}}_{m},x_{m,1},y_{m,1}\right),\ldots ,\left({\hat{t}}_{m},x_{m,q_{m}^{\prime }},y_{m,q_{m}^{\prime }}\right)\right\}. \end{array}$$

For i=1,…,m and $j=1,\ldots ,q_{i}^{\prime }$, we denote

$$\begin{array}{cc} \; & {\hat{u}}_{1,i,j}=x_{i,j}⊕f_{1}\left({\hat{t}}_{i}\right)\;\mathrm{and}\;{\hat{u}}_{2,i,j}=x_{i,j}⊕f_{2}\left({\hat{t}}_{i}\right). \end{array}$$

For convenience to describe, we rewrite the sets $U_{0}^{1}$ and $U_{0}^{2}$ as

$$\begin{array}{c} U_{0}^{1}=\left\{{\hat{u}}_{1,i,j}:1\leq i\leq m,1\leq j\leq q_{i}^{\prime }\right\}\;\mathrm{and}\;U_{0}^{2}=\left\{{\hat{u}}_{2,i,j}:1\leq i\leq m,1\leq j\leq q_{i}^{\prime }\right\}. \end{array}$$

Let $U_{1}^{+}=\left(U_{1,1},U_{2,1},U_{0}^{1}\right)$ and $U_{2}^{+}=\left(U_{2,2},U_{1,2},U_{0}^{2}\right)$. Then the following proposition holds.

**Proposition** **A1.**
*With notations as above, we have*

(i) *All sets in $U_{1}^{+}$ (resp. $U_{2}^{+}$) are disjoint, i.e., $U_{1,1}\cap U_{2,1}=\emptyset$, $U_{1,1}\cap U_{0}^{1}=\emptyset$, and $U_{2,1}\cap U_{0}^{1}=\emptyset$ (resp. $U_{2,2}\cap U_{1,2}=\emptyset$, $U_{2,2}\cap U_{0}^{2}=\emptyset$, and $U_{1,2}\cap U_{0}^{2}=\emptyset$).*
(ii) *$U_{1}^{+}$ is inner disjoint with $U_{1}$ and $U_{2}^{+}$ is inner disjoint with $U_{2}$.*

**Proof.** We first prove (i). From the fact $\tau \notin \Gamma_{8}$, one can conclude that $U_{1,1}\cap U_{2,1}=\emptyset$. By definition of $Q_{X_{1}}$ and $Q_{0}$, $U_{1,1}\cap U_{0}^{1}=\emptyset$ holds. By combining the fact $\tau \notin \Gamma_{8}$ and the disjoint property of $Q_{X_{2}}$ and $Q_{0}$, one has $U_{2,1}\cap U_{0}^{1}=\emptyset$. We can conclude that $U_{2,2}\cap U_{1,2}=\emptyset$, $U_{2,2}\cap U_{0}^{2}=\emptyset$, and $U_{1,2}\cap U_{0}^{2}=\emptyset$ in a similar way. Next we prove (ii) by enumerating all possible cases. For $U_{1,1}$, the definition of $Q_{X_{1}}$ means that $U_{1,1}\cap U_{1}=\emptyset$; $U_{1,1}\cap {\tilde{U}}_{1}=\emptyset$ comes from the fact $\tau \notin \Gamma_{7}$. For $U_{2,1}$, $U_{2,1}\cap U_{1}=\emptyset$ holds due to the fact $\tau \notin \Gamma_{6}$; $U_{2,1}\cap {\tilde{U}}_{1}=\emptyset$ can be obtained from the fact $\tau \notin \Gamma_{8}$ and the disjoint property between $Q_{X_{2}}$ and $Q_{U_{2}}$. For $U_{0}^{1}$, the definition of $Q_{0}$ means $U_{0}^{1}\cap U_{1}=\emptyset$; $U_{0}^{1}\cap {\tilde{U}}_{1}=\emptyset$ holds for the reason that $\tau \notin \Gamma_{7}$ and $Q_{0}$ is disjoint with $Q_{U_{2}}$. For $U_{2,2}$, the definition of $Q_{X_{2}}$ means that $U_{2,2}\cap U_{2}=\emptyset$; $U_{2,2}\cap {\tilde{U}}_{2}=\emptyset$ comes from the fact $\tau \notin \Gamma_{6}$. For $U_{1,2}$, one has $U_{1,2}\cap U_{2}=\emptyset$ since $\tau \notin \Gamma_{7}$; $U_{1,2}\cap {\tilde{U}}_{2}=\emptyset$ holds due to the fact $\tau \notin \Gamma_{8}$ and the disjoint property between $Q_{X_{1}}$ and $Q_{U_{1}}$. For $U_{0}^{2}$, one has $U_{0}^{2}\cap U_{2}=\emptyset$ by the definition of $Q_{0}$; $U_{0}^{2}\cap {\tilde{U}}_{2}=\emptyset$ holds for the reason that $\tau \notin \Gamma_{6}$ and $Q_{0}$ is disjoint with $Q_{U_{1}}$.   □

Until now *P* is fixed on $p_{1}$ input-output pairs from $U_{1}$ to $V_{1}$, $\alpha_{2}$ input-output pairs from ${\tilde{U}}_{1}$ from ${\tilde{V}}_{1}$, $p_{2}$ input-output pairs from $U_{2}$ to $V_{2}$, and $\alpha_{1}$ input-output pairs from ${\tilde{U}}_{2}$ to ${\tilde{V}}_{2}$. Based on these facts, the next work is to choose other possible compatible items for $V_{1,1}=P_{1}\left(U_{1,1}\right)$, $V_{2,1}=P_{1}\left(U_{2,1}\right)$, $V_{0}^{1}=P_{1}\left(U_{0}^{1}\right)$, $V_{1,2}=P_{2}\left(U_{1,2}\right)$, $V_{2,2}=P_{2}\left(U_{2,2}\right)$, and $V_{0}^{2}=P_{2}\left(U_{0}^{2}\right)$ to extend the fixed input-output pairs of permutations $P_{1}$ and $P_{2}$, respectively.

Note that once the items in $V_{1,1}=P_{1}\left(U_{1,1}\right)$ are fixed, then the corresponding items in $V_{1,2}=P_{2}\left(U_{1,2}\right)$ are uniquely determined since these two sets are both derived from $Q_{X_{1}}$. Similarly, the choices for items in $V_{2,2}=P_{2}\left(U_{2,2}\right)$ (resp. $V_{0}^{1}=P_{1}\left(U_{0}^{1}\right)$) uniquely determine the items in $V_{2,1}=P_{1}\left(U_{2,1}\right)$ (resp. $V_{0}^{2}=P_{2}\left(U_{0}^{2}\right)$). Then we sample all possible items for these sets through three steps.

$\mathrm{Step}\;I.\;\mathrm{Construct}\;V_{1,1}=P_{1}\left(U_{1,1}\right)\;\mathrm{and}\;V_{1,2}=P_{2}\left(U_{1,2}\right)$.

Recall that $X_{u}^{1}=\left\{\left(t,x,y\right)\in Q_{F}:x⊕f_{1}\left(t\right)=u\right\}$ and $U_{1,1}=\left\{u_{1,1},\ldots ,u_{1,\alpha_{1,1}}\right\}$. Let $N_{X_{1}}$ be the number of $\alpha_{1,1}$-wise tuples of distinct values $\left(v_{1,1},\ldots ,v_{1,\alpha_{1,1}}\right)$ in ${\left\{0,1\right\}}^{n}\backslash V_{1}\cup {\tilde{V}}_{1}$ satisfying the following two conditions:

(i) For each $i\in [\alpha_{1,1}]$ and each $\left(t,x,y\right)\in X_{u_{1,i}}^{1}$, $v_{1,i}⊕f_{1}\left(t\right)⊕f_{2}\left(t\right)⊕y\notin V_{2}\cup {\tilde{V}}_{2}$.
(ii) For each $i\in [\alpha_{1,1}]$ and $\left(t,x,y\right)\in X_{u_{1,i}}^{1}$, $v_{1,i}⊕f_{1}\left(t\right)⊕f_{2}\left(t\right)⊕y$ is distinct from the values $v_{1,j}⊕f_{1}\left(t^{\prime }\right)⊕f_{2}\left(t^{\prime }\right)⊕y^{\prime }$, for j<i and $\left(t^{\prime },x^{\prime },y^{\prime }\right)\in X_{u_{1,j}}^{1}$.

Now we count the number of all possible distinct tuples $\left(v_{1,1},\ldots ,v_{1,\alpha_{1,1}}\right)$ in ${\left\{0,1\right\}}^{n}\backslash V_{1}\cup {\tilde{V}}_{1}$ satisfying the above two conditions. First, we have ${|\left\{0,1\right\}}^{n}\backslash V_{1}\cup {\tilde{V}}_{1}|=2^{n}-\left(p_{1}+\alpha_{2}\right)$. The first condition can remove at most $\left(\right|V_{2}\left|+\right|{\tilde{V}}_{2}\left|)\cdot \right|X_{u_{1,i}}^{1}|=\left(p_{2}+\alpha_{1}\right)\cdot \left|X_{u_{1,i}}^{1}\right|$ values, and the final condition can exclude at most $|X_{u_{1,i}}^{1}|\cdot \sum_{j=1}^{i-1}|X_{u_{1,j}}^{1}|\leq \alpha_{1,2}\cdot \left|X_{u_{1,i}}^{1}\right|$ values for each choice of $v_{1,i}$. By combining above facts, one gets that

(A4) $$\begin{array}{c} N_{X_{1}}\geq \prod_{i=1}^{\alpha_{1,1}}(2^{n}-p_{1}-\alpha_{2}-\left(i-1\right)-\left(p_{2}+\alpha_{1}+\alpha_{1,2}\right)|X_{u_{1,i}}^{1}\left|\right). \end{array}$$

In Condition (ii), for each $i\in [\alpha_{1,1}]$ and $\left(t,x,y\right)\neq \left(t^{\prime },x^{\prime },y^{\prime }\right)\in X_{u_{1,i}}^{1}$, it holds that $v_{1,i}⊕f_{1}\left(t\right)⊕f_{2}\left(t\right)⊕y\neq v_{1,i}⊕f_{1}\left(t^{\prime }\right)⊕f_{2}\left(t^{\prime }\right)⊕y^{\prime }$ (which is equivalent to $f_{1}\left(t\right)⊕f_{2}\left(t\right)⊕y\neq f_{1}\left(t^{\prime }\right)⊕f_{2}\left(t^{\prime }\right)⊕y^{\prime }$) from the fact $\tau \notin \Gamma_{4}$. After choosing any tuple of distinct values $v_{1,i}\in {\left\{0,1\right\}}^{n}\backslash V_{1}\cup {\tilde{V}}_{1}$ such that Conditions (i) and (ii) hold, we define two corresponding sets as follows:

$$\begin{array}{cc} \; & V_{1,1}=\left\{v_{1,1},\ldots ,v_{1,\alpha_{1,1}}\right\},\\ \; & V_{1,2}=\left\{v_{1,i}⊕f_{1}\left(t\right)⊕f_{2}\left(t\right)⊕y:i=1,\ldots ,\alpha_{1,1}\;and\;\left(t,x,y\right)\in X_{u_{1,i}}^{1}\right\}. \end{array}$$

From the above discussion, we know that all values in $V_{1,1}$ are distinct, and all values in $V_{1,2}$ are also distinct. By the choice of $v_{1,i}$, it holds that $V_{1,1}\cap \left(V_{1}\cup {\tilde{V}}_{1}\right)=\emptyset$ and $V_{1,2}\cap \left(V_{2}\cup {\tilde{V}}_{2}\right)=\emptyset$. After this step, $P_{1}$ is fixed on $\alpha_{1,1}$ input-output pairs from $U_{1,1}$ to $V_{1,1}$, and $P_{2}$ is fixed on $\alpha_{1,2}$ input-output pairs from $U_{1,2}$ to $V_{1,2}$.

$\mathrm{Step}\;II.\;\mathrm{Construct}\;V_{2,2}=P_{2}\left(U_{2,2}\right)\;\mathrm{and}\;V_{2,1}=P_{1}\left(U_{2,1}\right)$.

We next deal with $Q_{X_{2}}$. Recall that $U_{2,2}=\left\{u_{2,1},\ldots ,u_{2,\alpha_{2,2}}\right\}$ and $X_{u}^{2}=\left\{\left(t,x,y\right)\in Q_{F}:x⊕f_{2}\left(t\right)=u\right\}$. Let $N_{X_{2}}$ be the number of $\alpha_{2,2}$-wise tuples of distinct values $\left(v_{2,1},\ldots ,v_{2,\alpha_{2,2}}\right)$ in ${\left\{0,1\right\}}^{n}\backslash V_{2}\cup {\tilde{V}}_{2}\cup V_{1,2}$ such that the following two conditions hold:

(i) For each $i\in [\alpha_{2,2}]$ and each $\left(t,x,y\right)\in X_{u_{2,i}}^{2}$, $v_{2,i}⊕f_{1}\left(t\right)⊕f_{2}\left(t\right)⊕y\notin V_{1}\cup {\tilde{V}}_{1}\cup V_{1,1}$.
(ii) For each $i\in [\alpha_{2,2}]$ and $\left(t,x,y\right)\in X_{u_{2,i}}^{2}$, $v_{2,i}⊕f_{1}\left(t\right)⊕f_{2}\left(t\right)⊕y$ is distinct from the values $v_{2,j}⊕f_{1}\left(t^{\prime }\right)⊕f_{2}\left(t^{\prime }\right)⊕y^{\prime }$, for j<i and $\left(t^{\prime },x^{\prime },y^{\prime }\right)\in X_{u_{2,j}}^{2}$.

Now we count the number of all possible distinct tuples $\left(v_{2,1},\ldots ,v_{2,\alpha_{2,2}}\right)$ in ${\left\{0,1\right\}}^{n}\backslash V_{2}\cup {\tilde{V}}_{2}\cup V_{1,2}$ satisfying above two conditions. It is easy to see that ${|\left\{0,1\right\}}^{n}\backslash V_{2}\cup {\tilde{V}}_{2}\cup V_{1,2}|=2^{n}-\left(p_{2}+\alpha_{1}+\alpha_{1,2}\right)$. The first condition can remove at most $\left(\right|V_{1}\left|+\right|{\tilde{V}}_{1}\left|+\right|V_{1,1}\left|)\cdot \right|X_{u_{2,i}}^{2}|=\left(p_{1}+\alpha_{2}+\alpha_{1,1}\right)\cdot \left|X_{u_{2,i}}^{2}\right|$ values, and the final condition can exclude at most $|X_{u_{2,i}}^{2}|\cdot \sum_{j=1}^{i-1}|X_{u_{2,j}}^{2}|\leq \alpha_{2,1}\cdot \left|X_{u_{2,i}}^{2}\right|$ values for each choice of $v_{2,i}$. Then we can bound $N_{X_{2}}$ as

(A5) NX2≥∏i=1α2,2(2n−p2−α1−α1,2−(i−1)−(p1+α2+α1,1+α2,1)|Xu2,i2|).

In Condition (ii), for each *i* and $\left(t,x,y\right)\neq \left(t^{\prime },x^{\prime },y^{\prime }\right)\in X_{u_{2,i}}^{2}$, it holds that $v_{2,i}⊕f_{1}\left(t\right)⊕f_{2}\left(t\right)⊕y\neq v_{2,i}⊕f_{1}\left(t^{\prime }\right)⊕f_{2}\left(t^{\prime }\right)⊕y^{\prime }$ (which is equivalent to $f_{1}\left(t\right)⊕f_{2}\left(t\right)⊕y\neq f_{1}\left(t^{\prime }\right)⊕f_{2}\left(t^{\prime }\right)⊕y^{\prime }$) since otherwise τ would satisfy Condition (B-5). Similarly, we define two sets as:

$$\begin{array}{cc} \; & V_{2,2}=\left\{v_{2,1},\ldots ,v_{2,\alpha_{2,2}}\right\},\\ \; & V_{2,1}=\left\{v_{2,i}⊕f_{1}\left(t\right)⊕f_{2}\left(t\right)⊕y:i=1,\ldots ,\alpha_{2,2}\;\mathrm{and}\;\left(t,x,y\right)\in X_{u_{2,i}}^{2}\right\}. \end{array}$$

By the discussion above, all values in $V_{2,1}$ are distinct and all values in $V_{2,2}$ are also distinct. Then $V_{2,1}\cap \left(V_{1}\cup {\tilde{V}}_{1}\cup V_{1,1}\right)=\emptyset$ and $V_{2,2}\cap \left(V_{2}\cup {\tilde{V}}_{2}\cup V_{1,2}\right)=\emptyset$ hold from the choice of $v_{2,i}$. After this step, $P_{2}$ is fixed on $\alpha_{2,2}$ input-output pairs from $U_{2,2}$ to $V_{2,2}$, and $P_{1}$ is fixed on $\alpha_{2,1}$ input-output pairs from $U_{2,1}$ to $V_{2,1}$.

$\mathrm{Step}\;III.\;\mathrm{Construct}\;V_{0}^{1}=P_{1}\left(U_{0}^{1}\right)\;\mathrm{and}\;V_{0}^{2}=P_{2}\left(U_{0}^{2}\right)$.

It remains to sample all possible compatible values in $V_{0}^{1}$ and $V_{0}^{2}$. First, we denote $p_{1}^{\prime }$ and $p_{2}^{\prime }$ as

$$\begin{array}{cc} \; & p_{1}^{\prime }=\left|V_{1}\cup {\tilde{V}}_{1}\cup V_{1,1}\cup V_{2,1}\right|=p_{1}+\alpha_{2}+\alpha_{1,1}+\alpha_{2,1},\\ \; & p_{2}^{\prime }=\left|V_{2}\cup {\tilde{V}}_{2}\cup V_{2,2}\cup V_{1,2}\right|=p_{2}+\alpha_{1}+\alpha_{1,2}+\alpha_{2,2}. \end{array}$$

Recall that $U_{0}^{1}=\left\{{\hat{u}}_{1,i,j}:1\leq i\leq m,1\leq j\leq q_{i}^{\prime }\right\}$ and $U_{0}^{2}=\left\{{\hat{u}}_{2,i,j}:1\leq i\leq m,1\leq j\leq q_{i}^{\prime }\right\}$. Let $N_{0}$ be the number of $q^{\prime }$-wise tuples of distinct values ${\left({\hat{v}}_{1,i,j}\right)}_{1\leq i\leq m,1\leq j\leq q_{i}^{\prime }}$ in ${\left\{0,1\right\}}^{n}\backslash V_{1}\cup {\tilde{V}}_{1}\cup V_{1,1}\cup V_{2,1}$ such that the following two conditions hold:

(i) For each i=1,…,m and $j=1,\ldots ,q_{i}^{\prime }$, ${\hat{v}}_{1,i,j}⊕f_{1}\left({\hat{t}}_{i}\right)⊕f_{2}\left({\hat{t}}_{i}\right)⊕y_{i,j}\notin V_{2}\cup {\tilde{V}}_{2}\cup V_{1,2}\cup V_{2,2}$.
(ii) For each i=1,…,m and $j=1,\ldots ,q_{i}^{\prime }$, ${\hat{v}}_{1,i,j}⊕f_{1}\left({\hat{t}}_{i}\right)⊕f_{2}\left({\hat{t}}_{i}\right)⊕y_{i,j}$ is distinct from the values ${\hat{v}}_{1,k,l}⊕f_{1}\left({\hat{t}}_{k}\right)⊕f_{2}\left({\hat{t}}_{k}\right)⊕y_{k,l}$ for k<i and $l\in [q_{k}^{\prime }]$. Furthermore, ${\hat{v}}_{1,i,j}⊕f_{1}\left({\hat{t}}_{i}\right)⊕f_{2}\left({\hat{t}}_{i}\right)⊕y_{i,j}$ should also be distinct from the values ${\hat{v}}_{1,i,j^{\prime }}⊕f_{1}\left({\hat{t}}_{i}\right)⊕f_{2}\left({\hat{t}}_{i}\right)⊕y_{i,j^{\prime }}$ with $j^{\prime }<j$.

Except these two conditions, each ${\hat{v}}_{1,i,j}$ must be chosen distinctly from each other. First, one has ${|\left\{0,1\right\}}^{n}\backslash V_{1}\cup {\tilde{V}}_{1}\cup V_{1,1}\cup V_{2,1}|=2^{n}-p_{1}^{\prime }$. Then we count the number of all possible distinct tuples ${\left({\hat{v}}_{1,i,j}\right)}_{1\leq i\leq m,1\leq j\leq q_{i}^{\prime }}$ satisfying above two conditions. The first condition can exclude at most $p_{2}^{\prime }$ values, and the second condition can exclude at most $\sum_{k=1}^{i-1}q_{k}^{\prime }-j+1$ values for each choice of ${\hat{v}}_{1,i,j}$. Furthermore, ${\hat{v}}_{1,i,j}$ should not be same to previous $\sum_{k=1}^{i-1}q_{k}^{\prime }-j+1$ items. Based on these facts, one can obtain that

(A6) $$\begin{array}{cc} N_{0} & \geq \prod_{i=1}^{m}\prod_{j=1}^{q_{i}^{\prime }}\left(2^{n}-p_{1}^{\prime }-p_{2}^{\prime }-2\sum_{k=1}^{i-1}q_{k}^{\prime }-2\left(j-1\right)\right). \end{array}$$

Until now, we have chosen $N_{X_{1}}\cdot N_{X_{2}}\cdot N_{0}$ possible values for ${\left(v_{1,i}\right)}_{1\leq i\leq \alpha_{1,1}}$, ${\left(v_{2,i}\right)}_{1\leq i\leq \alpha_{2,2}}$, and ${\left({\hat{v}}_{1,i,j}\right)}_{1\leq i\leq m,1\leq j\leq q_{i}^{\prime }}$ satisfying all above conditions. By this way, when conditioned on $E_{U_{1}}\wedge E_{U_{2}}\wedge \left(P_{i}⊢Q_{P_{i}},i=1,2\right)$, the event $E_{X_{1}}\wedge E_{X_{2}}\wedge E_{0}$ happens means that $P_{1}$ (resp. $P_{2}$) is fixed on exactly $\alpha_{1,1}+\alpha_{2,1}+q^{\prime }$ (resp. $\alpha_{1,2}+\alpha_{2,2}+q^{\prime }$) “new” input-output pairs from $U_{1,1}\cup U_{2,1}\cup U_{0}^{1}$ (resp. $U_{2,2}\cup U_{1,2}\cup U_{0}^{2}$) to $V_{1,1}\cup V_{2,1}\cup V_{0}^{1}$ (resp. $V_{2,2}\cup V_{1,2}\cup V_{0}^{2}$). Finally, we conclude that

(A7) $$\begin{array}{c} p^{\prime \prime }\left(\tau \right)\geq \frac{N_{X_{1}}\cdot N_{X_{2}}\cdot N_{0}}{{\left(2^{n}-p_{1}-\alpha_{2}\right)}_{\alpha_{1,1}+\alpha_{2,1}+q^{\prime }}{\left(2^{n}-p_{2}-\alpha_{1}\right)}_{\alpha_{1,2}+\alpha_{2,2}+q^{\prime }}}. \end{array}$$

From (A3) and (A7), one has

(A8) $$\begin{array}{c} p\left(\tau \right)\geq \frac{N_{X_{1}}\cdot N_{X_{2}}\cdot N_{0}}{{\left(2^{n}-p_{1}\right)}_{\alpha_{2}+\alpha_{1,1}+\alpha_{2,1}+q^{\prime }}{\left(2^{n}-p_{2}\right)}_{\alpha_{1}+\alpha_{1,2}+\alpha_{2,2}+q^{\prime }}}. \end{array}$$

Combining (21) and (A8), we get

(A9) $$\begin{array}{cc} \frac{\mathrm{Pr}[T_{\mathrm{re}}=\tau ]}{\mathrm{Pr}[T_{\mathrm{id}}=\tau ]} & \geq \frac{N_{X_{1}}\cdot N_{X_{2}}\cdot N_{0}\cdot 2^{nq}}{{\left(2^{n}-p_{1}\right)}_{\alpha_{2}+\alpha_{1,1}+\alpha_{2,1}+q^{\prime }}{\left(2^{n}-p_{2}\right)}_{\alpha_{1}+\alpha_{1,2}+\alpha_{2,2}+q^{\prime }}}\\ \; & =\underset{R_{X_{1}}}{\underset{︸}{\frac{N_{X_{1}}}{{\left(2^{n}-p_{1}-\alpha_{2}\right)}_{\alpha_{1,1}}}}}\cdot \underset{R_{X_{2}}}{\underset{︸}{\frac{N_{X_{2}}}{{\left(2^{n}-p_{2}-\alpha_{1}-\alpha_{1,2}\right)}_{\alpha_{2,2}}}}}\\ \; & \;\;\;\;\cdot \underset{R_{0}}{\underset{︸}{\frac{N_{0}\cdot 2^{nq^{\prime }}}{{\left(2^{n}-p_{1}^{\prime }\right)}_{q^{\prime }}{\left(2^{n}-p_{2}^{\prime }\right)}_{q^{\prime }}}}}\\ \; & \;\;\;\;\cdot \underset{\geq 1(**)}{\underset{︸}{\frac{2^{n(q-q^{\prime })}}{{\left(2^{n}-p_{1}\right)}_{\alpha_{2}}\cdot {\left(2^{n}-p_{1}-\alpha_{2}-\alpha_{1,1}\right)}_{\alpha_{2,1}}\cdot {\left(2^{n}-p_{2}\right)}_{\alpha_{1}+\alpha_{1,2}}}}}, \end{array}$$

where (**) can be obtained from the fact ${\left(2^{n}-p_{1}\right)}_{\alpha_{2}}\cdot$${\left(2^{n}-p_{1}-\alpha_{2}-\alpha_{1,1}\right)}_{\alpha_{2,1}}\cdot$${\left(2^{n}-p_{2}\right)}_{\alpha_{1}+\alpha_{1,2}}$$\leq {2^{n}}^{\left(\alpha_{2}+\alpha_{2,1}+\alpha_{1}+\alpha_{1,2}\right)}={2^{n}}^{\left(q-q^{\prime }\right)}$.

First, $R_{X_{1}}$ can be bounded as follows:

(A10) $$\begin{array}{cc} R_{X_{1}} & \geq \frac{\prod_{i=1}^{\alpha_{1,1}}(2^{n}-p_{1}-\alpha_{2}-\left(i-1\right)-\left(p_{2}+\alpha_{1}+\alpha_{1,2}\right)|X_{u_{1,i}}^{1}\left|\right)}{{\left(2^{n}-p_{1}-\alpha_{2}\right)}_{\alpha_{1,1}}}\\ \; & \geq \prod_{i=1}^{\alpha_{1,1}}\left(1-\frac{\left(p_{2}+\alpha_{1}+\alpha_{1,2}\right)\left|X_{u_{1,i}}^{1}\right|}{2^{n}-p_{1}-\alpha_{2}-\left(i-1\right)}\right)\\ \; & \geq 1-\frac{\left(p_{2}+\alpha_{1}+\alpha_{1,2}\right)\sum_{i=1}^{\alpha_{1,1}}\left|X_{u_{1,i}}^{1}\right|}{2^{n}-p_{1}-\alpha_{2}-\alpha_{1,1}}\\ \; & =1-\frac{\left(p_{2}+\alpha_{1}+\alpha_{1,2}\right)\alpha_{1,2}}{2^{n}-p_{1}-\alpha_{2}-\alpha_{1,1}}\\ \; & \geq 1-\frac{2\sqrt{q}\left(p_{2}+2\sqrt{q}\right)}{2^{n}}, \end{array}$$

where the last equality holds from the fact $\alpha_{1}\leq \sqrt{q}$, $\alpha_{1,2}\leq \sqrt{q}$, and $p_{1}+\alpha_{2}+\alpha_{1,1}\leq p_{1}+2q\leq p_{1}+p_{2}+3q\leq 2^{n-1}$.

Next, we can bound $R_{X_{2}}$ as

(A11) $$\begin{array}{cc} R_{X_{2}} & \geq \frac{\prod_{i=1}^{\alpha_{2,2}}(2^{n}-p_{2}-\alpha_{1}-\alpha_{1,2}-\left(i-1\right)-\left(p_{1}+\alpha_{2}+\alpha_{1,1}+\alpha_{2,1}\right)|X_{u_{2,i}}^{2}\left|\right)}{{\left(2^{n}-p_{2}-\alpha_{1}-\alpha_{1,2}\right)}_{\alpha_{2,2}}}\\ \; & \geq \prod_{i=1}^{\alpha_{2,2}}\left(1-\frac{\left(p_{1}+\alpha_{2}+\alpha_{1,1}+\alpha_{2,1}\right)\left|X_{u_{2,i}}^{2}\right|}{2^{n}-p_{2}-\alpha_{1}-\alpha_{1,2}-\alpha_{2,2}}\right)\\ \; & \geq 1-\frac{\left(p_{1}+\alpha_{2}+\alpha_{1,1}+\alpha_{2,1}\right)\sum_{i=1}^{\alpha_{2,2}}\left|X_{u_{2,i}}^{2}\right|}{2^{n}-p_{2}-\alpha_{1}-\alpha_{1,2}-\alpha_{2,2}}\\ \; & =1-\frac{\left(p_{1}+\alpha_{2}+\alpha_{1,1}+\alpha_{2,1}\right)\alpha_{2,1}}{2^{n}-p_{2}-\alpha_{1}-\alpha_{1,2}-\alpha_{2,2}}\\ \; & \geq 1-\frac{2\sqrt{q}\left(p_{1}+3\sqrt{q}\right)}{2^{n}}, \end{array}$$

where the last equality holds from the fact $\alpha_{2}\leq \sqrt{q}$, $\alpha_{1,1}\leq \sqrt{q}$, $\alpha_{2,1}\leq \sqrt{q}$, and $p_{2}+\alpha_{1}+\alpha_{1,2}+\alpha_{2,2}\leq p_{2}+3q\leq p_{1}+p_{2}+3q\leq 2^{n-1}$.

Finally, $R_{0}$ can be bounded in the following way:

(A12) $$\begin{array}{cc} R_{0} & \geq \frac{\prod_{i=1}^{m}2^{nq_{i}^{\prime }}\cdot \prod_{j=0}^{q_{i}^{\prime }-1}\left(2^{n}-p_{1}^{\prime }-p_{2}^{\prime }-2\sum_{k=1}^{i-1}q_{k}^{\prime }-2j\right)}{{\left(2^{n}-p_{1}^{\prime }\right)}_{q^{\prime }}{\left(2^{n}-p_{2}^{\prime }\right)}_{q^{\prime }}}\\ \; & =\prod_{i=1}^{m}\left(\frac{2^{nq_{i}^{\prime }}\cdot \prod_{j=0}^{q_{i}^{\prime }-1}\left(2^{n}-p_{1}^{\prime }-p_{2}^{\prime }-2\sum_{k=1}^{i-1}q_{k}^{\prime }-2j\right)}{{\left(2^{n}-p_{1}^{\prime }-\sum_{k=1}^{i-1}q_{k}^{\prime }\right)}_{q_{i}^{\prime }}{\left(2^{n}-p_{2}^{\prime }-\sum_{k=1}^{i-1}q_{k}^{\prime }\right)}_{q_{i}^{\prime }}}\right)\\ \; & =\prod_{i=1}^{m}\prod_{j=0}^{q_{i}^{\prime }-1}\left(\frac{2^{n}\left(2^{n}-p_{1}^{\prime }-p_{2}^{\prime }-2\sum_{k=1}^{i-1}q_{k}^{\prime }-2j\right)}{\left(2^{n}-p_{1}^{\prime }-\sum_{k=1}^{i-1}q_{k}^{\prime }-j\right)\left(2^{n}-p_{2}^{\prime }-\sum_{k=1}^{i-1}q_{k}^{\prime }-j\right)}\right)\\ \; & \overset{\left(a\right)}{\geq }\prod_{i=1}^{m}\left(1-\frac{4q_{i}^{\prime }\left(p_{1}^{\prime }+\sum_{k=1}^{i}q_{k}^{\prime }\right)\left(p_{2}^{\prime }+\sum_{k=1}^{i}q_{k}^{\prime }\right)}{2^{2n}}\right)\\ \; & \overset{\left(b\right)}{\geq }\prod_{i=1}^{m}\left(1-\frac{4q_{i}^{\prime }{\left(p_{1}+p_{2}+2q\right)}^{2}}{2^{2n}}\right)\\ \; & \overset{\left(c\right)}{\geq }\left(1-\frac{4q^{\prime }{\left(p_{1}+p_{2}+2q\right)}^{2}}{2^{2n}}\right)\\ \; & \geq \left(1-\frac{4q{\left(p_{1}+p_{2}+2q\right)}^{2}}{2^{2n}}\right), \end{array}$$

where (a) holds by Lemma 2 when one sets $A=q_{i}^{\prime }$, $B=p_{1}^{\prime }+\sum_{k=1}^{i-1}q_{k}^{\prime }$, and $C=p_{2}^{\prime }+\sum_{k=1}^{i-1}q_{k}^{\prime }$ such that $A+B\leq p_{1}^{\prime }+q^{\prime }=p_{1}+q+\alpha_{1,1}-\alpha_{1,2}-\alpha_{1}\leq p_{1}+q+\alpha_{1,1}\leq p_{1}+2q+p_{2}\leq 2^{n-1}$ and $A+C\leq p_{2}+q+\alpha_{2,2}\leq p_{1}+2q+p_{2}\leq 2^{n-1}$, (b) follows as $p_{1}^{\prime }+\sum_{k=1}^{i}q_{k}^{\prime }\leq p_{1}^{\prime }+q^{\prime }\leq p_{1}+q+\alpha_{1,1}\leq p_{1}+2q+p_{2}$ and $p_{2}^{\prime }+\sum_{k=1}^{i}q_{k}^{\prime }\leq p_{2}^{\prime }+q^{\prime }\leq p_{2}+q+\alpha_{2,2}\leq p_{1}+2q+p_{2}$, and (c) follows as $q^{\prime }=\sum_{k=1}^{m}q_{k}^{\prime }$.

We finally lower bound $\frac{\mathrm{Pr}[T_{\mathrm{re}}=\tau ]}{\mathrm{Pr}[T_{\mathrm{id}}=\tau ]}$, from (A9), (A10), (A11), and (A12), as

$$\begin{array}{c} \frac{\mathrm{Pr}[T_{\mathrm{re}}=\tau ]}{\mathrm{Pr}[T_{\mathrm{id}}=\tau ]}\geq 1-\frac{4q{\left(p_{1}+p_{2}+2q\right)}^{2}}{2^{2n}}-\frac{2\sqrt{q}\left(p_{1}+p_{2}\right)}{2^{n}}-\frac{10q}{2^{n}}. \end{array}$$

## Appendix C. Upper Bound on ${\mathrm{Bad}}_{M_{1}}$

In this part, we upper bound each term $\mathrm{Pr}[T_{\mathrm{id}}\in \Gamma_{i}^{\prime }]$ for i∈[15] one by one.

Bounding (C-1), (C-2), and (C-3): For any $\left(t_{i},x_{i},y_{i}\right)\in {\bar{Q}}_{F}$ and $\left(u_{j},v_{j}\right)$, $\left(u_{j^{\prime }},v_{j^{\prime }}\right)\in {\bar{Q}}_{P}$, by the $\epsilon_{1}$-regular property of $\left(f_{1},f_{2}\right)$, one has

$$\mathrm{Pr}\left[\left(f_{1}\left(t_{i}\right)=x_{i}⊕u_{j}\right)\wedge \left(f_{2}\left(t_{i}\right)=x_{i}⊕v_{j^{\prime }}\right)\right]\leq \epsilon_{1}^{2}.$$

Since the number of all possible tuples for $\left(\left(t_{i},x_{i},y_{i}\right),u_{j},v_{j^{\prime }}\right)$ is at most $qp^{2}$, by union bound, it holds that

$$\mathrm{Pr}\left[T_{\mathrm{id}}\in \Gamma_{1}^{\prime }\right]\leq qp^{2}\epsilon_{1}^{2}.$$

Similarly, we can bound the probabilities of (C-2) and (C-3) as

$$\mathrm{Pr}\left[T_{\mathrm{id}}\in \Gamma_{2}^{\prime }\right]\leq qp^{2}\epsilon_{1}^{2}\;\mathrm{and}\;\mathrm{Pr}\left[T_{\mathrm{id}}\in \Gamma_{3}^{\prime }\right]\leq qp^{2}\epsilon_{1}^{2}.$$

Bounding (C-4) and (C-5): For any fixed construction queries $(t_{i},x_{i},y_{i}),$$\left(t_{i^{\prime }},x_{i^{\prime }},y_{i^{\prime }}\right)\in {\bar{Q}}_{F}$, and $\left(u_{j},v_{j}\right)\in {\bar{Q}}_{P}$, by the same reason as above, we have

$$\mathrm{Pr}\left[\left(f_{1}\left(t_{i}\right)=x_{i}⊕u_{j}\right)\wedge \left(f_{2}\left(t_{i}\right)=v_{j}⊕f_{1}\left(t_{i}\right)⊕y_{i}⊕x_{i^{\prime }}⊕f_{1}\left(t_{i^{\prime }}\right)\right)\right]\leq \epsilon_{1}^{2}.$$

Since there are at most $q^{2}p$ possible unordered pairs for $\{\left(t_{i},x_{i},y_{i}\right),\left(t_{i^{\prime }},x_{i^{\prime }},y_{i^{\prime }}\right)$, $\left(u_{j},v_{j})\right\}$, by union bound, one obtains that

$$\mathrm{Pr}\left[T_{\mathrm{id}}\in \Gamma_{4}^{\prime }\right]\leq q^{2}p\epsilon_{1}^{2},\;\mathrm{and}\;\mathrm{similarly},\;\mathrm{Pr}\left[T_{\mathrm{id}}\in \Gamma_{5}^{\prime }\right]\leq q^{2}p\epsilon_{1}^{2}.$$

Bounding (C-6) and (C-7): For any fixed distinct construction queries $(t_{i},x_{i},y_{i}),$$\left(t_{i^{\prime }},x_{i^{\prime }},y_{i^{\prime }}\right)\in {\bar{Q}}_{F}$ and $u_{j}\in U$, from the $\epsilon_{1}$-regular and $\epsilon_{2}$-AXU properties of $\left(f_{1},f_{2}\right)$, one has

$$\mathrm{Pr}\left[\left(f_{1}\left(t_{i}\right)=x_{i}⊕u_{j}\right)\wedge \left(f_{2}\left(t_{i}\right)⊕f_{1}\left(t_{i^{\prime }}\right)=x_{i}⊕x_{i^{\prime }}\right)\right]\leq \epsilon_{1}\epsilon_{2}.$$

Since there are at most $q^{2}p$ possible unordered pairs for $\{\left(t_{i},x_{i},y_{i}\right),\left(t_{i^{\prime }},x_{i^{\prime }},y_{i^{\prime }}\right)$, $u_{j}\}$, by union bound, it holds that

$$\mathrm{Pr}\left[T_{\mathrm{id}}\in \Gamma_{6}^{\prime }\right]\leq q^{2}p\epsilon_{1}\epsilon_{2},\;\mathrm{and}\;\mathrm{similarly},\;\mathrm{Pr}\left[T_{\mathrm{id}}\in \Gamma_{7}^{\prime }\right]\leq q^{2}p\epsilon_{1}\epsilon_{2}.$$

Bounding (C-8) and (C-9): For any two distinct construction queries $(t_{i},x_{i},y_{i}),$$\left(t_{i^{\prime }},x_{i^{\prime }},y_{i^{\prime }}\right)\in {\bar{Q}}_{F}$, one can conclude

$$\mathrm{Pr}\left[\left(f_{1}\left(t_{i}\right)⊕f_{1}\left(t_{i^{\prime }}\right)=x_{i}⊕x_{i^{\prime }}\right)\wedge \left(f_{2}\left(t_{i}\right)⊕f_{2}\left(t_{i^{\prime }}\right)=f_{1}\left(t_{i}\right)⊕f_{1}\left(t_{i^{\prime }}\right)⊕y_{i}⊕y_{i^{\prime }}\right)\right]\leq \epsilon_{2}^{2}$$

from the $\epsilon_{2}$-AXU property of $\left(f_{1},f_{2}\right)$. In particular, when $t_{i}=t_{i^{\prime }}$, the above probability is in fact zero since in this case we have $f_{1}\left(t_{i}\right)⊕f_{1}\left(t_{i^{\prime }}\right)=0$ but $x_{i}\neq x_{i^{\prime }}$. Then by summing over all q2 possible unordered pairs $\left\{\left(t_{i},x_{i},y_{i}\right),\left(t_{i^{\prime }},x_{i^{\prime }},y_{i^{\prime }}\right)\right\}$, one has

Pr[Tid∈Γ8′]≤q2·ϵ22≤q2ϵ222,andsimilarly,Pr[Tid∈Γ9′]≤q2ϵ222.

Bounding (C-10): For any $\left(t_{i},x_{i},y_{i}\right),\left(t_{i^{\prime }},x_{i^{\prime }},y_{i^{\prime }}\right)$, and $\left(t_{i^{\prime \prime }},x_{i^{\prime \prime }},y_{i^{\prime \prime }}\right)\in {\bar{Q}}_{F}$ with $\left(t_{i},x_{i},y_{i}\right)$$\neq (t_{i^{\prime }},x_{i^{\prime }},y_{i^{\prime }})$ and $\left(t_{i},x_{i},y_{i}\right)\neq \left(t_{i^{\prime \prime }},x_{i^{\prime \prime }},y_{i^{\prime \prime }}\right)$, one can conclude, from the $\epsilon_{2}$-AXU property of $\left(f_{1},f_{2}\right)$, that

$$\mathrm{Pr}\left[\left(f_{1}\left(t_{i}\right)⊕f_{1}\left(t_{i^{\prime }}\right)=x_{i}⊕x_{i^{\prime }}\right)\wedge \left(f_{2}\left(t_{i}\right)⊕f_{2}\left(t_{i^{\prime \prime }}\right)=x_{i}⊕x_{i^{\prime \prime }}\right)\right]\leq \epsilon_{2}^{2}$$

Note that the number of all possible tuples $\left\{\left(t_{i},x_{i},y_{i}\right),\left(t_{i^{\prime }},x_{i^{\prime }},y_{i^{\prime }}\right),\left(t_{i^{\prime \prime }},x_{i^{\prime \prime }},y_{i^{\prime \prime }}\right)\right\}$ is at most $q^{3}$ so that one has

$$\mathrm{Pr}\left[T_{\mathrm{id}}\in \Gamma_{10}^{\prime }\right]\leq q^{3}\epsilon_{2}^{2}.$$

Bounding (C-11), (C-12), (C-13), and (C-14): We deal with bad conditions (C-11) and (C-13) together by using the fact that

$$\mathrm{Pr}\left[T_{\mathrm{id}}\in \Gamma_{11}^{\prime }\cup \Gamma_{13}^{\prime }\right]\leq \mathrm{Pr}\left[T_{\mathrm{id}}\in \Gamma_{13}^{\prime }\right]+\mathrm{Pr}\left[T_{\mathrm{id}}\in \Gamma_{11}^{\prime }\backslash \Gamma_{13}^{\prime }\right].$$

We first consider how to upper bound $\mathrm{Pr}[T_{\mathrm{id}}\in \Gamma_{13}^{\prime }]$. Recall that ${\bar{\alpha }}_{1}=\left|\left\{\left(t,x,y\right)\in {\bar{Q}}_{F}:x⊕f_{1}\left(t\right)\in U\right\}\right|$. Then the expectation value of ${\bar{\alpha }}_{1}$ can be computed as

E[α¯1]=∑(t,x,y)∈Q¯F:∑u∈U:Pr[x⊕f1(t)=u]≤pqϵ1

due to the $\epsilon_{1}$-regular property of $\left(f_{1},f_{2}\right)$. By Markov’s inequality, one has

$$\mathrm{Pr}\left[T_{\mathrm{id}}\in \Gamma_{13}^{\prime }\right]\leq \frac{E[{\bar{\alpha }}_{1}]}{\sqrt{q}}=p\sqrt{q}\epsilon_{1}.$$

Under the condition ${\bar{\alpha }}_{1}\leq \sqrt{q}$, there are at most q/2 pairs $\{\left(\left(t_{i},x_{i},y_{i}\right),u_{j}\right)$, $(\left(t_{i^{\prime }},x_{i^{\prime }},y_{i^{\prime }}\right),$$u_{j^{\prime }})\}$ such that $x_{i}⊕f_{1}\left(t_{i}\right)=u_{j}$ and $x_{i^{\prime }}⊕f_{1}\left(t_{i^{\prime }}\right)=u_{j^{\prime }}$ where $\left(t_{i},x_{i},y_{i}\right)\neq \left(t_{i^{\prime }},x_{i^{\prime }},y_{i^{\prime }}\right)\in {\bar{Q}}_{F}$ and $\left(u_{j},v_{j}\right),\left(u_{j^{\prime }},v_{j^{\prime }}\right)\in {\bar{Q}}_{P}$. In this case, since the random variables $y_{i}$ and $y_{i^{\prime }}$ are independently and uniformly distributed over ${\left\{0,1\right\}}^{n}$, one can conclude that

$$\mathrm{Pr}\left[v_{j}⊕f_{1}\left(t_{i}\right)⊕f_{2}\left(t_{i}\right)⊕y_{i}=v_{j^{\prime }}⊕f_{1}\left(t_{i^{\prime }}\right)⊕f_{2}\left(t_{i^{\prime }}\right)⊕y_{i^{\prime }}\right]\leq \frac{1}{2^{n}}.$$

By summing over all these q/2 possible pairs, we have

$$\mathrm{Pr}\left[T_{\mathrm{id}}\in \Gamma_{11}^{\prime }\backslash \Gamma_{13}^{\prime }\right]\leq \frac{q}{2^{n+1}}.$$

and so that it holds that

$$\mathrm{Pr}\left[T_{\mathrm{id}}\in \Gamma_{11}^{\prime }\cup \Gamma_{13}^{\prime }\right]\leq p\sqrt{q}\epsilon_{1}+\frac{q}{2^{n+1}}.$$

Similarly, we can obtain that

$$\mathrm{Pr}\left[T_{\mathrm{id}}\in \Gamma_{12}^{\prime }\cup \Gamma_{14}^{\prime }\right]\leq p\sqrt{q}\epsilon_{1}+\frac{q}{2^{n+1}}.$$

Bounding (C-15): To upper bound $\mathrm{Pr}[T_{\mathrm{id}}\in \Gamma_{15}^{\prime }]$, we first define the random variable ${\bar{T}}_{F}=\left|\left\{\left(\left(t,x,y\right),\left(t^{\prime },x^{\prime },y^{\prime }\right)\right)\in {\bar{Q}}_{F}\times {\bar{Q}}_{F}:\left(t,x,y\right)\neq \left(t^{\prime },x^{\prime },y^{\prime }\right),x⊕f_{1}\left(t\right)=x^{\prime }⊕f_{1}\left(t^{\prime }\right)\right\}\right|$. By definition of ${\bar{\beta }}_{1}$, it holds that

$${\bar{\beta }}_{1}=\left|\left\{\left(t,x,y\right)\in {\bar{Q}}_{F}:\exists \left(t,x,y\right)\neq \left(t^{\prime },x^{\prime },y^{\prime }\right),x⊕f_{1}\left(t\right)=x^{\prime }⊕f_{1}\left(t^{\prime }\right)\right\}\right|\leq {\bar{T}}_{F}.$$

Thus, $E\left[{\bar{\beta }}_{1}\right]\leq E\left[{\bar{T}}_{F}\right]$. Then the expectation value of ${\bar{T}}_{F}$ can be bounded as

E[T¯F]=∑(t,x,y)≠(t′,x′,y′)∈Q¯F2:Pr[x⊕f1(t)=x′⊕f1(t′)]≤q2ϵ22

from the $\epsilon_{2}$-AXU property of $\left(f_{1},f_{2}\right)$. By Markov’s inequality, we have

$$\mathrm{Pr}\left[{\bar{\beta }}_{1}\geq \sqrt{q}\right]\leq \frac{E[\beta_{1}]}{\sqrt{q}}\leq \frac{E[{\bar{T}}_{F}]}{\sqrt{q}}\leq \frac{q^{3/2}\epsilon_{2}}{2}.$$

Similarly, one has

$$\mathrm{Pr}\left[{\bar{\beta }}_{2}\geq \sqrt{q}\right]\leq \frac{q^{3/2}\epsilon_{2}}{2}.$$

Finally, by combining the above two facts, it holds that

$$\mathrm{Pr}\left[T_{\mathrm{id}}\in \Gamma_{15}^{\prime }\right]\leq \mathrm{Pr}\left[\left({\bar{\beta }}_{1}\geq \sqrt{q}\right)\vee \left({\bar{\beta }}_{2}\geq \sqrt{q}\right)\right]\leq q^{3/2}\epsilon_{2}.$$

## Appendix D. More Details in Proof of Lemma 6

First we have

(A13) $$\begin{array}{cc} R_{1}\left(\alpha \right) & \geq \frac{\prod_{k=0}^{\alpha_{3}-1}\left(2^{n}-\left(q-\alpha_{5}+\alpha_{6}+\alpha \right)-p-k-\left(p+\alpha +q+\alpha_{3}\right)\left|{\bar{X}}_{u_{3,k+1}}^{1}\right|\right)}{\prod_{k=0}^{\alpha_{3}-1}\left(2^{n}-p-k\right)}\\ \; & \geq \prod_{k=0}^{\alpha_{3}-1}\left(1-\frac{q-\alpha_{5}+\alpha_{6}+\alpha }{2^{n}-p-k}-\frac{\left(p+\alpha +q+\alpha_{3}\right)\left|{\bar{X}}_{u_{3,k+1}}^{1}\right|}{2^{n}-p-k}\right)\\ \; & \geq \prod_{k=0}^{\alpha_{3}-1}\left(1-\frac{q+\alpha_{6}+\alpha }{2^{n}-p-\alpha_{3}}-\frac{\left(p+\alpha +q+\alpha_{3}\right)\left|{\bar{X}}_{u_{3,k+1}}^{1}\right|}{2^{n}-p-\alpha_{3}}\right)\\ \; & \geq 1-\frac{\alpha_{3}\left(q+\alpha_{6}+\alpha \right)}{2^{n}-p-\alpha_{3}}-\frac{\left(p+\alpha +q+\alpha_{3}\right)\sum_{k=0}^{\alpha_{3}-1}\left|{\bar{X}}_{u_{3,k+1}}^{1}\right|}{2^{n}-p-\alpha_{3}}\\ \; & \overset{\left(d\right)}{\geq }1-\frac{2\alpha_{3}\left(q+\alpha_{6}+\alpha \right)}{2^{n}}-\frac{2\left(p+\alpha +q+\alpha_{3}\right)\alpha_{4}}{2^{n}}\\ \; & \overset{\left(e\right)}{\geq }1-\frac{2\sqrt{q}\left(q+\sqrt{q}+q/2^{\frac{n}{3}}\right)}{2^{n}}-\frac{2\left(p+q/2^{\frac{n}{3}}+q+\sqrt{q}\right)\sqrt{q}}{2^{n}}\\ \; & =1-\frac{4q^{3/2}}{2^{n}}-\frac{2p\sqrt{q}}{2^{n}}-\frac{4q}{2^{n}}-\frac{4q^{3/2}}{2^{\frac{4n}{3}}}\\ \; & \geq 1-\frac{8q^{3/2}}{2^{n}}-\frac{2p\sqrt{q}}{2^{n}}-\frac{4q}{2^{n}}, \end{array}$$

where (d) follows as $p+\alpha_{3}\leq p+\sqrt{q}\leq 2^{n-1}$ so that $2^{n}-p-\alpha_{3}>2^{n-1}$, and (e) follows as $\alpha_{3},\alpha_{4},\alpha_{6}\leq \sqrt{q}$ and $\alpha \leq M\leq q/2^{\frac{n}{3}}$.

Then, the item $R_{2}\left(\alpha \right)$ can be bounded as

(A14) $$\begin{array}{cc} R_{2}\left(\alpha \right) & \geq \frac{\prod_{k=0}^{\alpha_{6}-1}\left(2^{n}-\left(p+\alpha_{3}+q+\alpha \right)-k-\left(p+q+\alpha_{3}+\alpha_{6}+\alpha \right)\left|{\bar{X}}_{v_{6,k+1}}^{2}\right|\right)}{\prod_{k=0}^{\alpha_{6}-1}\left(2^{n}-p-\alpha_{3}-k\right)}\\ \; & \geq \prod_{k=0}^{\alpha_{6}-1}\left(1-\frac{q+\alpha }{2^{n}-p-\alpha_{3}-k}-\frac{\left(p+q+\alpha_{3}+\alpha_{6}+\alpha \right)\left|{\bar{X}}_{v_{6,k+1}}^{2}\right|}{2^{n}-p-\alpha_{3}-k}\right)\\ \; & \overset{\left(f\right)}{\geq }1-\frac{2\left(q+\alpha \right)\alpha_{6}}{2^{n}}-\frac{2\left(p+q+\alpha_{3}+\alpha_{6}+\alpha \right)\sum_{k=0}^{\alpha_{6}-1}\left|{\bar{X}}_{v_{6,k+1}}^{2}\right|}{2^{n}}\\ \; & \geq 1-\frac{2\left(q+\alpha \right)\alpha_{6}}{2^{n}}-\frac{2\left(p+q+\alpha_{3}+\alpha_{6}+\alpha \right)\alpha_{5}}{2^{n}}\\ \; & \overset{\left(g\right)}{\geq }1-\frac{2\left(q+q/2^{\frac{n}{3}}\right)\sqrt{q}}{2^{n}}-\frac{2\left(p+q+2\sqrt{q}+q/2^{\frac{n}{3}}\right)\sqrt{q}}{2^{n}}\\ \; & \geq 1-\frac{8q^{3/2}}{2^{n}}-\frac{2p\sqrt{q}}{2^{n}}-\frac{4q}{2^{n}}, \end{array}$$

where (f) follows as $p+\alpha_{3}+k\leq p+\alpha_{3}+\alpha_{6}\leq p+2\sqrt{q}\leq 2^{n-1}$ so that $2^{n}-p-k>2^{n-1}$ and (g) follows as $\alpha_{3},\alpha_{5},\alpha_{6}\leq \sqrt{q}$ and $\alpha \leq M\leq q/2^{\frac{n}{3}}$.

Finally, $R_{0}\left(\alpha \right)$ can be bounded in the following.

(A15) $$\begin{array}{cc} R_{0}\left(\alpha \right) & =\frac{2^{n{\bar{q}}^{\prime }}\cdot N_{S}\left(\alpha \right)\cdot N_{0}\left(\alpha \right)}{{\left(2^{n}-p^{\prime }\right)}_{2{\bar{q}}^{\prime \prime }+3\alpha }}\\ \; & \geq \frac{{\left({\bar{q}}^{\prime }\right)}_{2\alpha }}{\alpha !}\cdot \left(1-\epsilon_{0}\right)\cdot \frac{N_{0}\left(\alpha \right)\cdot 2^{n{\bar{q}}^{\prime }}}{{\left(2^{n}-p^{\prime }\right)}_{2{\bar{q}}^{\prime \prime }+3\alpha }}\\ \; & =\left(1-\epsilon_{0}\right)\cdot \frac{{\left({\bar{q}}^{\prime }\right)}_{2\alpha }}{\alpha !}\cdot \frac{2^{n{\bar{q}}^{\prime }}\cdot \prod_{i=1}^{m}\prod_{j=0}^{{\bar{q}}_{i}^{\prime \prime }-1}\left(2^{n}-2p^{\prime }-2{\bar{q}}^{\prime }-2\alpha -2\sum_{k=1}^{i-1}{\bar{q}}_{k}^{\prime \prime }-2j\right)}{{\left(2^{n}-p^{\prime }\right)}_{{\bar{q}}^{\prime \prime }+\alpha +{\bar{q}}^{\prime }}}\\ \; & =\left(1-\epsilon_{0}\right)\cdot \frac{{\left({\bar{q}}^{\prime }\right)}_{2\alpha }}{{\left({\bar{q}}^{\prime }\right)}_{\alpha }{\left({\bar{q}}^{\prime }\right)}_{\alpha }}\\ \; & \;\;\;\;\cdot \frac{2^{n{\bar{q}}^{\prime }}\cdot \prod_{i=1}^{m}\prod_{j=0}^{{\bar{q}}_{i}^{\prime \prime }-1}\left(2^{n}-2p^{\prime }-2{\bar{q}}^{\prime }-2\alpha -2\sum_{k=1}^{i-1}{\bar{q}}_{k}^{\prime \prime }-2j\right)}{{\left(2^{n}-p^{\prime }-{\bar{q}}^{\prime }\right)}_{{\bar{q}}^{\prime \prime }+\alpha }{\left(2^{n}-p^{\prime }-{\bar{q}}^{\prime }\right)}_{{\bar{q}}^{\prime \prime }+\alpha }}\\ \; & \;\;\;\;\cdot {\mathrm{Hyp}}_{2^{n}-p^{\prime },{\bar{q}}^{\prime },{\bar{q}}^{\prime }}\left(\alpha \right)\\ \; & =\left(1-\epsilon_{0}\right)\cdot \underset{B_{1}\left(\alpha \right)}{\underset{︸}{\frac{{\left({\bar{q}}^{\prime }\right)}_{2\alpha }}{{\left({\bar{q}}^{\prime }\right)}_{\alpha }{\left({\bar{q}}^{\prime }\right)}_{\alpha }}}}\cdot \underset{\geq 1}{\underset{︸}{\frac{{\left(2^{n}\right)}^{2\alpha }}{{\left(2^{n}-p^{\prime }-{\bar{q}}^{\prime }\right)}_{\alpha }^{2}}}}\cdot {\mathrm{Hyp}}_{2^{n}-p^{\prime },{\bar{q}}^{\prime },{\bar{q}}^{\prime }}\left(\alpha \right)\\ \; & \;\;\;\;\cdot \underset{B_{2}\left(\alpha \right)}{\underset{︸}{\frac{2^{n{\bar{q}}^{\prime \prime }}\cdot \prod_{i=1}^{m}\prod_{j=0}^{{\bar{q}}_{i}^{\prime \prime }-1}\left(2^{n}-2p^{\prime }-2{\bar{q}}^{\prime }-2\alpha -2\sum_{k=1}^{i-1}{\bar{q}}_{k}^{\prime \prime }-2j\right)}{{\left(2^{n}-p^{\prime }-{\bar{q}}^{\prime }-\alpha \right)}_{{\bar{q}}^{\prime \prime }}^{2}}}} \end{array}$$

For $B_{1}\left(\alpha \right)$, we have

(A16) $$\begin{array}{cc} B_{1}\left(\alpha \right) & \overset{\left(h\right)}{\geq }\frac{{\left({\bar{q}}^{\prime }-2M\right)}^{2\alpha }}{{\left({\bar{q}}^{\prime }\right)}^{2\alpha }}={\left(1-\frac{2M}{{\bar{q}}^{\prime }}\right)}^{2\alpha }\\ \; & \geq 1-\frac{4M\alpha }{{\bar{q}}^{\prime }}\geq 1-\frac{4\alpha }{2^{n/3}}\\ \; & \overset{\left(j\right)}{\geq }1-\frac{4q}{2^{2n/3}}, \end{array}$$

where (h) follows as q−i≥q−2M for 0≤i≤2α≤2M and ${\left({\bar{q}}^{\prime }\right)}_{\alpha }\leq {\left({\bar{q}}^{\prime }\right)}^{\alpha }$ and (j) follows as $\alpha \leq M\leq \frac{q}{2^{n/3}}$.

We then bound the $B_{2}\left(\alpha \right)$ as

(A17) $$\begin{array}{cc} B_{2}\left(\alpha \right) & =\frac{2^{n(\sum_{i=1}^{m}{\bar{q}}_{i}^{\prime \prime })}\prod_{i=1}^{m}\prod_{j=0}^{{\bar{q}}_{i}^{\prime \prime }-1}\left(2^{n}-2p^{\prime }-2{\bar{q}}^{\prime }-2\alpha -2\sum_{k=1}^{i-1}{\bar{q}}_{k}^{\prime \prime }-2j\right)}{\prod_{i=1}^{m}{\left(2^{n}-p^{\prime }-{\bar{q}}^{\prime }-\alpha -\sum_{k=1}^{i-1}{\bar{q}}_{k}^{\prime \prime }\right)}_{{\bar{q}}_{i}^{\prime \prime }}^{2}}\\ \; & =\prod_{i=1}^{m}\left(\frac{2^{n{\bar{q}}_{i}^{\prime \prime }}\prod_{j=0}^{{\bar{q}}_{i}^{\prime \prime }-1}\left(2^{n}-2p^{\prime }-2{\bar{q}}^{\prime }-2\alpha -2\sum_{k=1}^{i-1}{\bar{q}}_{k}^{\prime \prime }-2j\right)}{{\left(2^{n}-p^{\prime }-{\bar{q}}^{\prime }-\alpha -\sum_{k=1}^{i-1}{\bar{q}}_{k}^{\prime \prime }\right)}_{{\bar{q}}_{i}^{\prime \prime }}^{2}}\right)\\ \; & =\prod_{i=1}^{m}\prod_{j=0}^{{\bar{q}}_{i}^{\prime \prime }-1}\left(\frac{2^{n}\left(2^{n}-2p^{\prime }-2{\bar{q}}^{\prime }-2\alpha -2\sum_{k=1}^{i-1}{\bar{q}}_{k}^{\prime \prime }-2j\right)}{{\left(2^{n}-p^{\prime }-{\bar{q}}^{\prime }-\alpha -\sum_{k=1}^{i-1}{\bar{q}}_{k}^{\prime \prime }-j\right)}^{2}}\right)\\ \; & \overset{\left(k\right)}{\geq }\prod_{i=1}^{m}\left(1-\frac{4{\bar{q}}_{i}^{\prime \prime }{\left(p^{\prime }+{\bar{q}}^{\prime }+\alpha +\sum_{k=1}^{i}{\bar{q}}_{k}^{\prime \prime }\right)}^{2}}{2^{2n}}\right)\\ \; & \geq \prod_{i=1}^{m}\left(1-\frac{4{\bar{q}}_{i}^{\prime \prime }{\left(p^{\prime }+2{\bar{q}}^{\prime }\right)}^{2}}{2^{2n}}\right)\\ \; & \overset{\left(l\right)}{\geq }\left(1-\frac{4{\bar{q}}^{\prime \prime }{\left(p^{\prime }+2{\bar{q}}^{\prime }\right)}^{2}}{2^{2n}}\right)\\ \; & \geq \left(1-\frac{4q{\left(p+2q+6\sqrt{q}\right)}^{2}}{2^{2n}}\right), \end{array}$$

where (k) follows as Lemma 2 when we set $N=2^{n}$, $A={\bar{q}}_{i}^{\prime \prime }$ and $B=C=p^{\prime }+{\bar{q}}^{\prime }+\alpha +\sum_{k=1}^{i-1}{\bar{q}}_{k}^{\prime \prime }$ where it satisfies $A+B=A+C=p^{\prime }+{\bar{q}}^{\prime }+\alpha +\sum_{k=1}^{i}{\bar{q}}_{k}^{\prime \prime }\leq p^{\prime }+2{\bar{q}}^{\prime }\leq p+2q+6\sqrt{q}\leq 2^{n-1}$ from the assumption and (l) follows as ${\bar{q}}^{\prime \prime }=\sum_{i=1}^{m}{\bar{q}}_{i}^{\prime \prime }$.
